# Designed to Degrade: Tailoring Polyesters for Circularity

**DOI:** 10.1021/acs.chemrev.4c00032

**Published:** 2024-06-27

**Authors:** Celine V. Aarsen, Anna Liguori, Rebecca Mattsson, Mika H. Sipponen, Minna Hakkarainen

**Affiliations:** † Department of Fibre and Polymer Technology, KTH Royal Institute of Technology, Teknikringen 58, 100 44 Stockholm, Sweden; ‡ Department of Chemistry “G. Ciamician”, University of Bologna, Via Selmi 2, 40126 Bologna, Italy; § Department of Materials and Environmental Chemistry, Stockholm University, Svante Arrhenius väg 16C, 106 91 Stockholm, Sweden

## Abstract

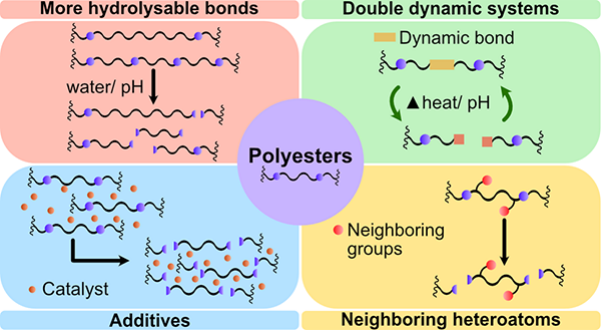

A powerful toolbox is needed to turn the linear plastic economy into circular. Development of materials designed for mechanical recycling, chemical recycling, and/or biodegradation in targeted end-of-life environment are all necessary puzzle pieces in this process. Polyesters, with reversible ester bonds, are already forerunners in plastic circularity: poly(ethylene terephthalate) (PET) is the most recycled plastic material suitable for mechanical and chemical recycling, while common aliphatic polyesters are biodegradable under favorable conditions, such as industrial compost. However, this circular design needs to be further tailored for different end-of-life options to enable chemical recycling under greener conditions and/or rapid enough biodegradation even under less favorable environmental conditions. Here, we discuss molecular design of the polyester chain targeting enhancement of circularity by incorporation of more easily hydrolyzable ester bonds, additional dynamic bonds, or degradation catalyzing functional groups as part of the polyester chain. The utilization of polyester circularity to design replacement materials for current volume plastics is also reviewed as well as embedment of green catalysts, such as enzymes in biodegradable polyester matrices to facilitate the degradation process.

## 1 Introduction

**Figure 1:**
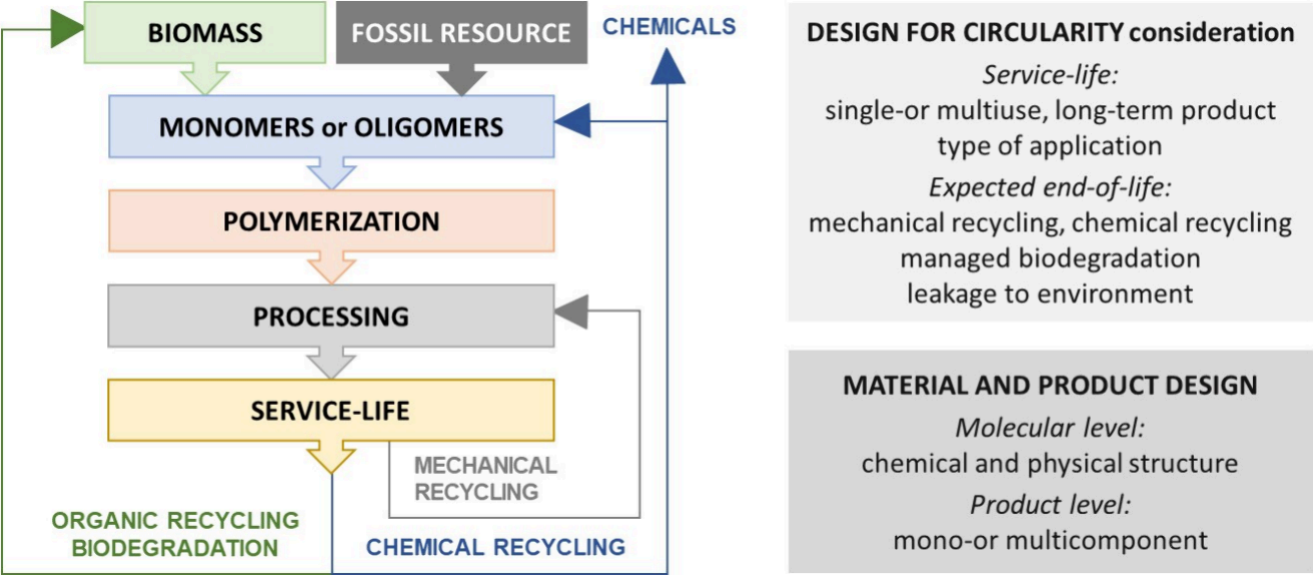
Simplified scheme over polymer circularity, where chemical recycling and biodegradation compliment mechanical recycling, and materials are designed for specific end-of-life scenarios.

The plastic waste problem, depletion of fossil-based resources, and intensifying climate change require concrete action to transform from linear to circular polymer materials.^1−3^ Within this paradigm shift, polyesters are expected to play a significant role. They have a large and tunable property window, and they are typically easy to process. In addition, the reversible ester bond can be utilized for chemical and organic (biodegradation) recycling processes.^4,5^ Still, the susceptibility of this bond to chemical hydrolysis and biodegradation varies considerably depending on the chemical and physical structure of the material and the end-of-life environment. To release the full potential of polyesters, their design can be tailored to fulfill the application specific requirements, as well as easy mechanical and chemical recyclability and/or biodegradability, depending on the specific application. Designing materials and products to circularity gives value to end-of-life plastics and reduces accumulation of waste, contributing to a more sustainable and circular economy.^6^ Mechanical recycling is currently the main commercial recycling route, however, not all products are suitable for mechanical recycling. Material degradation during use-phase, organic contamination, presence of harmful and/or unknown additives, product design (e.g., multicomponent materials, small formats), and small material volumes in case of less common materials are some limiting factors. There is also a limit to how many times a material can be mechanically recycled as significant property loss may occur after multiple rounds of mechanical recycling due to degradation phenomena. Chemical recycling and biodegradation are needed as complementary end-of-life pathways (Figure 1).

This review focuses on the molecular design of the polyester chain aiming at more facile circularity. How can polyesters be designed for faster (bio)degradation by introduction of ester bonds that are more susceptible to hydrolysis and biodegradation? How can chemical recycling under mild conditions or (bio)degradation under less favorable environmental conditions be facilitated by introduction of another type of reversible dynamic bond or degradation-catalyzing functional groups? Could embedding green catalysts, such as enzymes, in the polymer matrix be the route to (bio)degradation of the materials, even under less favorable environmental conditions? Last, the replacement of current commodity plastics, especially polyolefins, by more circular polyethylene-like polyesters is discussed.

### 1.1 Definitions

Some key terminologies are shortly defined here:

**Circular Economy**: In a circular economy, materials never become waste and nature is regenerated. As an example, polymer materials should be kept in circulation at their highest value through, e.g., reuse, mechanical and chemical recycling, and composting.

**Mechanical Recycling**: The most common commercial recycling process. Mechanical recycling turns plastic waste into secondary raw materials. The process typically consists of sorting of different plastic types, grinding, washing, formulation, extrusion to pellets, and finally reprocessing to new products, ideally without significantly change in chemical structure, molar mass, and properties. In practice, mechanical recycling is often downcycling to products with lower value compared to the original product.

**Chemical Recycling**: A general term for plastic recycling that includes changes in chemical structure to break the polymer chain into original monomers, oligomers, or other chemicals that can be used for manufacture of new polymers or other products. Several different technologies exist with significant differences in reaction conditions, such as temperature and the type of products obtained.

**Polymer Degradation**: Polymer degradation can be chemical, physical, mechanical, or biological, resulting in changes in the structure and properties of the material. Degradation is typically caused by external factors such as heat, light, water, chemicals, mechanical force, or microorganisms.

**Biodegradation**: Biodegradation is the breakdown of organic matter by microorganisms. This can be a multistep process, where the organic carbon is converted into humic substances, assimilated into the biomass or released as CO_2_, H_2_O, and/or CH_4_.

**Mineralization**: The last and ultimate step of biodegradation converts organic carbon into CO_2_ and H_2_O under aerobic conditions, and in addition, CH_4_ under anaerobic conditions. Biodegradation can be confirmed and the extent quantified by following the formation of CO_2_ or CH_4_.

**Surface and Bulk Erosion**: Surface erosion occurs at the surface of the material, allowing easy diffusion and release of the formed low molar mass compounds, while the remaining bulk material may retain its original molar mass for a long period of time. This is a common degradation process as microorganisms, enzymes, and even water might not be able to penetrate the bulk of the material. In bulk erosion, the degradation takes places throughout the whole material simultaneously, leading to faster molar mass decrease and possible entrapment of degradation products inside the material.

## 2 Chemical Recycling and Biodegradation

Industrial scale chemical recycling of plastics is still in its infancy. The interest is large, but breakthroughs are required to achieve more sustainable and commercially viable chemical recycling.^7^ The presence of ester bond in the main chain, has made the aromatic polyester, poly(ethylene terephthalate) (PET), the forerunner in this area. It was the first volume plastic to have pilot/semi-industrial scale processes, leading to chemicals that can be repolymerized to PET, theoretically enabling closed-loop recycling. The current commercial processes mainly utilize methanolysis or glycolysis,^8^ while hydrolysis catalyzed by alkaline or acidic conditions or enzymes^9^ is an additional option for closed-loop recycling. Furthermore, aminolysis and ammonolysis provide promising options for upcycling.^10^ The cost and environmental impact are still higher compared to the mechanical recycling of PET.^11^ At the same time, the processes are more favorable, and the recovered products have higher value compared with those from chemical recycling of other volume plastics thanks to the reversible ester bond in the main chain. The benefit of ester bond and similar processes are also expected to be viable for other polyesters.^4^ Ring-closing depolymerization (RcDP) is an attractive route for chemical recycling of polyesters produced by ring-opening polymerization (ROP).^12,13^ Here, interesting work has been performed by utilizing the thermodynamic equilibrium between ROP and RcDP to produce repeatedly recyclable polymer materials.^14,15^ This equilibrium can be influenced, e.g., by design of cyclic monomer structures that provide a suitable balance between polymerizability and chemical recyclability^16,17^ and by monomer–solvent interactions.^18,19^ It was also shown that transesterification can be utilized to upcycle aliphatic polyesters to value-added block copolymers.^20^ The chemical recyclability of polyesters could be further promoted, e.g., by introduction of a second reversible chemical bond or neighboring groups that can function as internal catalysts for the depolymerization process.^21,22^

**Figure 2:**
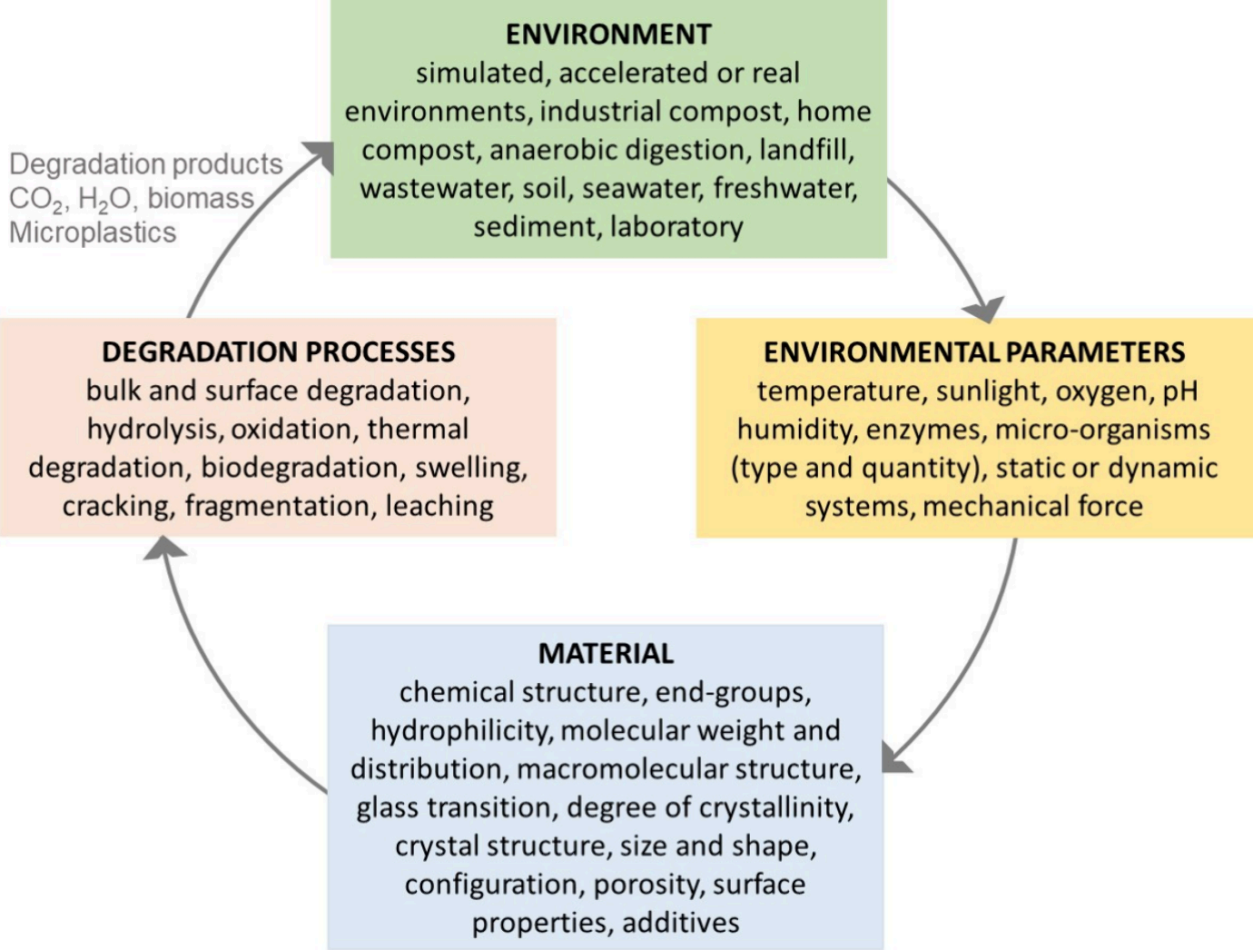
Complicated case of polymer degradation sensitive to small changes in chemical and physical structure of the polymer and degradation environment.

Organic recycling of plastics through biodegradation is an important puzzle piece and complementary in battling plastic pollution. Some plastics (e.g., agricultural and horticultural products and packaging that is contaminated by organic waste) are difficult to collect and recycle, leading currently to incineration, landfilling, or in worst case disposal in the environment.^23,24^ For these plastics, biodegradation is a valuable property, ideally leading to complete mineralization of the product under suitable environmental conditions.^25^ Current production of biodegradable or compostable polymer materials only correspond to less than 1% of total plastic production, and it is dominated by polyesters and thermoplastic starch.^26^ Ideally, the biodegradable plastic fulfills its function during service life and then rapidly degrades in predetermined environment through complete assimilation by microorganisms without any ecotoxicity or other negative impacts on the degradation environment. This is still a huge challenge; small changes in structure and composition of the plastic product or in the degradation environment can significantly influence the subsequent degradation rate.^27^ The different natural (e.g., marine, freshwater, forest soil) and man-made (e.g., industrial compost, home compost, agricultural soil) environments vary markedly with respect to conditions, such as temperature, humidity, sunlight, oxygen, and the type and concentration of microorganisms.^28^ It is also not easy to simulate natural environments under laboratory conditions to make reliable predictions of degradability.^29^ Last but not least, there is often a conflict between the application requirements and biodegradability, e.g., good water and oxygen barrier properties are wanted for packaging materials but counteract biodegradability and contribute to inadequate degradation rate. The complicated interplays of multiple material and environmental parameters influencing the degradation process are illustrated in Figure 2.

Biodegradation under aerobic conditions ultimately leads to CO_2_, H_2_O, and biomass. In the case of polyesters, the abiotic degradation of the high molar mass polymers and release of oligomeric products can further promote the mineralization by microorganisms.^30^ Introduction of specifically designed “weak” or reversible bonds in the polyester chain, in addition to the “regular” ester bonds, could provide a tool to maintain material performance while providing handles where this initial degradation can take place. The opening of these bonds then leads to oligomers that are more easily biodegraded. An alternative approach could be the introduction of internal catalysis in the form of heteroatom containing functional groups along the polymer chain or embedded enzymes that can catalyze the initial hydrolysis of the polymer chain when it comes in contact with humidity or aqueous environment. This kind of modification can provide a step forward in ensuring adequate degradation even under less ideal degradation conditions. Similar approaches could further provide a route to chemical recycling under milder conditions to recover and repolymerize the breakdown products ideally in a closed-loop.

## 3 Increasing Biodegradability by More Hydrolyzable Ester Bonds

### 3.1 Degradation of Commercial Biodegradable Polyesters

**Figure 3:**
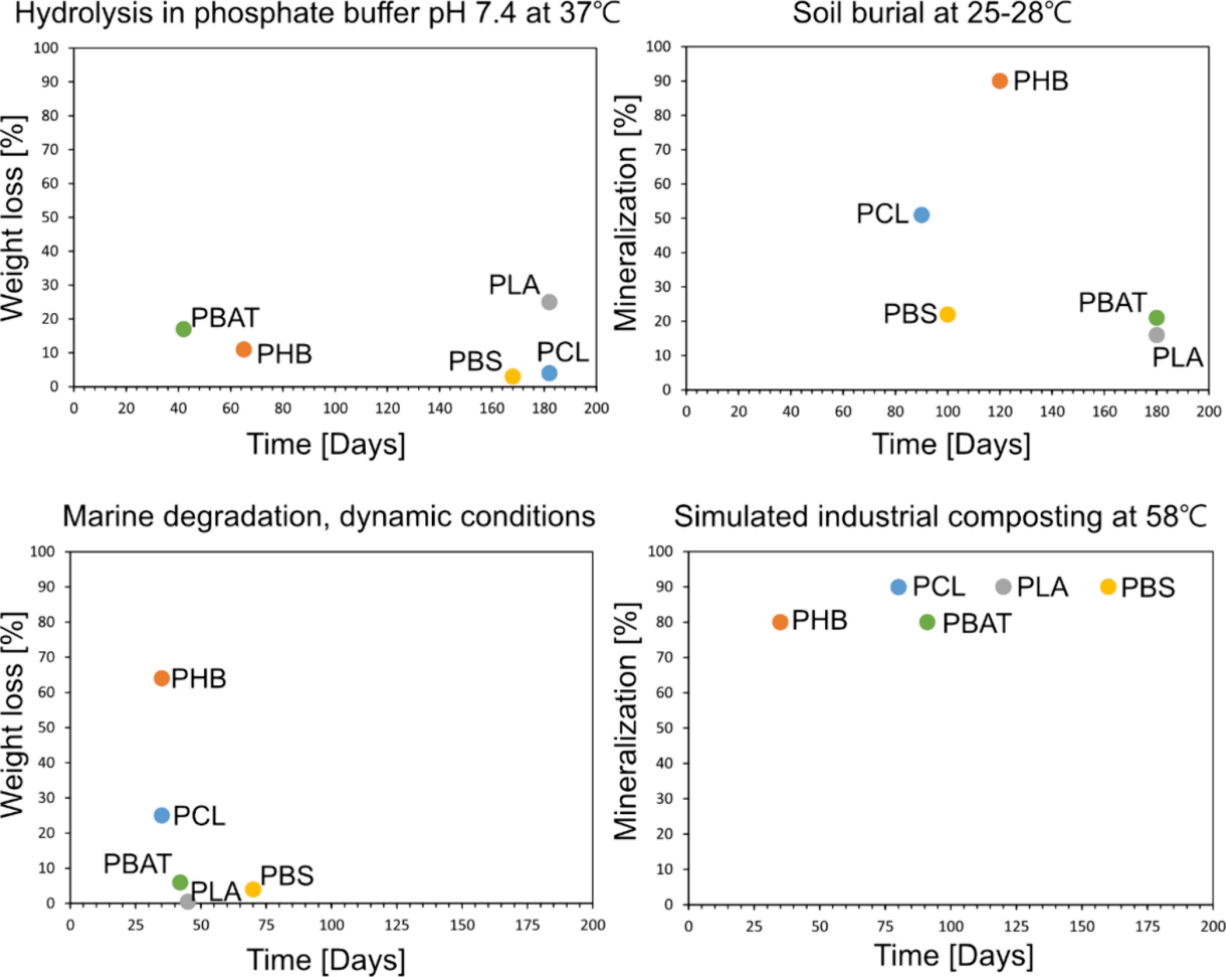
Examples of degradation rates of PLA, PHB, PCL, PBS, and PBAT in different environments (upper left) weight loss due to chemical hydrolysis in phosphate buffer (pH = 7.4) at 37 °C,^40−44^ (lower left) weight loss in dynamic marine environment,^44−46^ (upper right) mineralization during simulated soil burial at 25–28 °C,^47−50^ and (lower right) mineralization during simulated industrial composting at 58 °C.^32,51−54^

Degradation of aliphatic and aliphatic–aromatic polyesters in different environments has been widely studied in different laboratory and real environments and only a short overview is presented here.^31−33^ Some common representatives of this group are materials produced by polycondensation of diols and dicarboxylic acids, such as poly(butylene succinate) (PBS), poly(butylene succinate-*co*-adipate) (PBSA), and poly(butylene adipate-*co*-terephthalate) (PBAT) and materials typically produced by ring-opening polymerization, such as the polyhydroxy acids, polylactide (PLA), and polycaprolactone (PCL), and the microbial polyesters, poly(3-hydrohybutyrate) (PHB) and poly(3-hydroxybutyrate-*co*-3-hydroxyvalerate) (PHBV). However, even though the chemical structures of these biodegradable polyesters are relatively similar, this group of materials is not homogeneous in material properties, applications, and degradation behavior.^34,35^ As Figure 3 illustrates, the degradability and degradation rate can vary significantly depending on the specific plastic material and the specific environment.^36−38^ For example, the biodegradation rate of PHB and PCL in marine environment and under soil burial is typically significantly faster than the degradation rate of PLA, although they have longer aliphatic −CH_2_– segments and lower concentration of ester groups. This is likely explained by the better accessibility of the ester groups in PCL and PHB to enzymes, while the −CH_3_ substituent in PLA on the carbon next to the ester group causes steric hindrance. At the same time, the lower concentration of ester groups and longer aliphatic segments between ester groups reduces the chemical hydrolysis rate of PCL and PHB compared to PLA. In general, the chemical hydrolysis rates are relatively slow at 20–37 °C, while the hydrolysis rate is significantly accelerated if testing is performed at 50–60 °C.^31^ PBS is typically only certified to biodegrade in industrial compost, and PLA in industrial compost and during anaerobic digestion, while PHB is expected to biodegrade even in marine and fresh water, soil, and home compost.^39^ The biodegradation rate of PBAT is highly dependent on the aromatic content, but grades certified for biodegradation in industrial and home compost and under soil burial are available. In addition to chemical structure, many material parameters influence degradation rate (see Figure 2), such as molar mass, degree of crystallinity, glass transition temperature, mechanical properties, and size of the specimen to mention a few.

**Figure 4:**
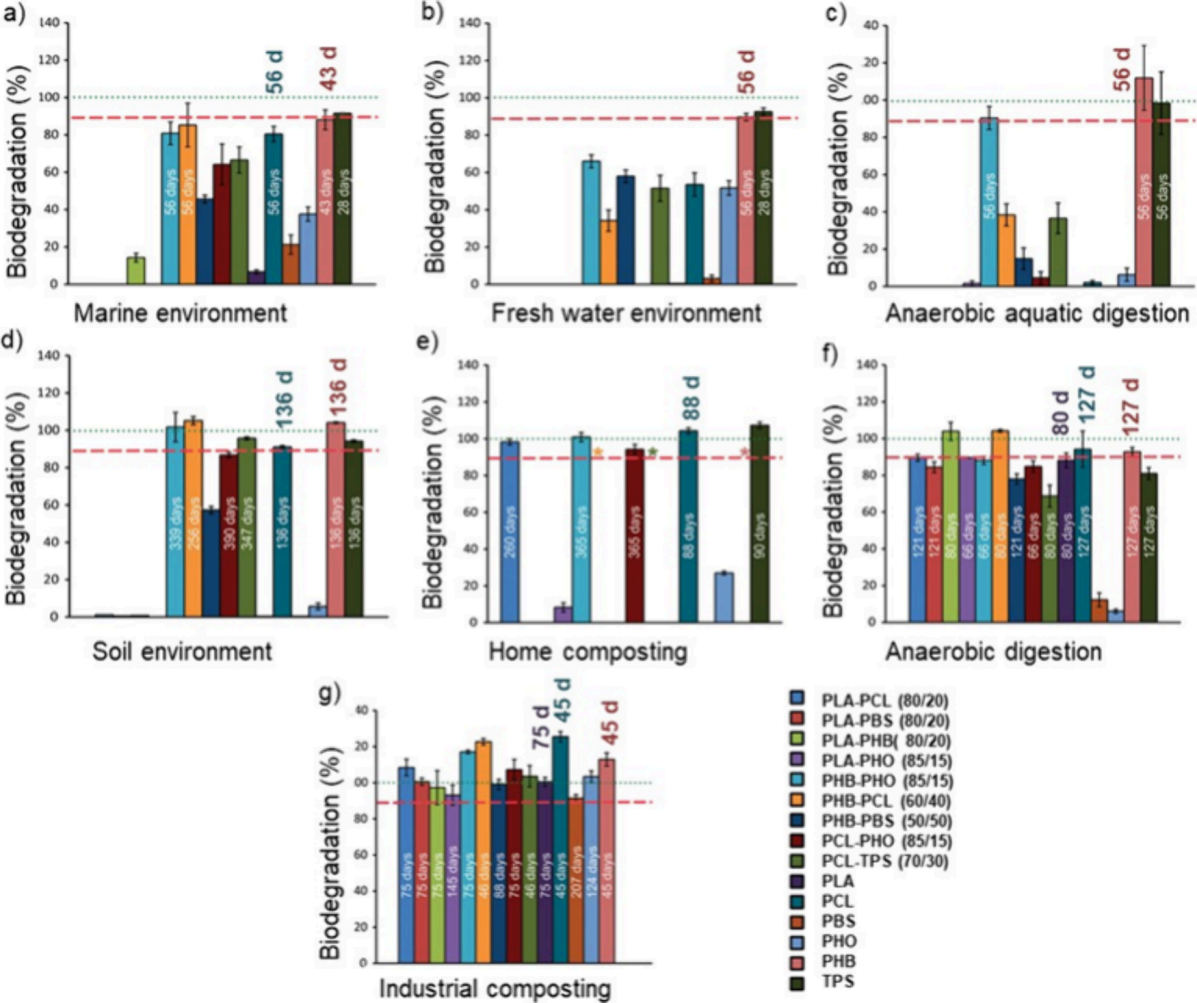
Biodegradation of aliphatic polyesters (PLA, PCL, PBS, PHB, and polyhydroxyoctanoate (PHO)), their blends and thermoplastic starch (TPS) in different managed and unmanaged environments. (a) Marine environment (ASTM D6691, 30 °C), (b) fresh water environment (ISO 14851, 21 °C), (c) anaerobic aquatic digestion (ISO 11734, 35 °C), (d) soil environment (ISO 17556), (e) home compost (ISO 14855, 28 °C), (f) anaerobic digestion (ISO 15985, 52 °C), and (g) industrial compost (ISO 14855, 58 °C). Biodegradation was calculated in relation to biodegradation of cellulose (green dotted line), and 90% biodegradation is marked with red dashed line. Adapted with permission from ref (32). Copyright 2018 American Chemical Society.

Figure 4 further demonstrates the large differences in degradation rates of common polyesters depending on the type of environment and the specific polyester.^32^ As an example, PHB reached the 90% biodegradation level in all tested environments. The >90–100% biodegradation was reached after approximately 43 days in marine, 56 days in freshwater and anaerobic aquatic digestion, 136 days in soil, 127 days in anaerobic digestion, and 45 days in industrial compost, showing the clear influence of degradation environment even for readily biodegradable material such as PHB. The differences in degradation rate were even more significant for more slowly degrading materials, such as PLA, which only degraded under anaerobic digestion and industrial composting conditions. Furthermore, the significant difference in degradation rate between the controlled waste management environments (industrial compost and anaerobic digestion) and the unmanaged natural and man-made environments is clearly shown. The biodegradation property thereby needs to be coupled to specific environmental conditions.

**Figure 5:**
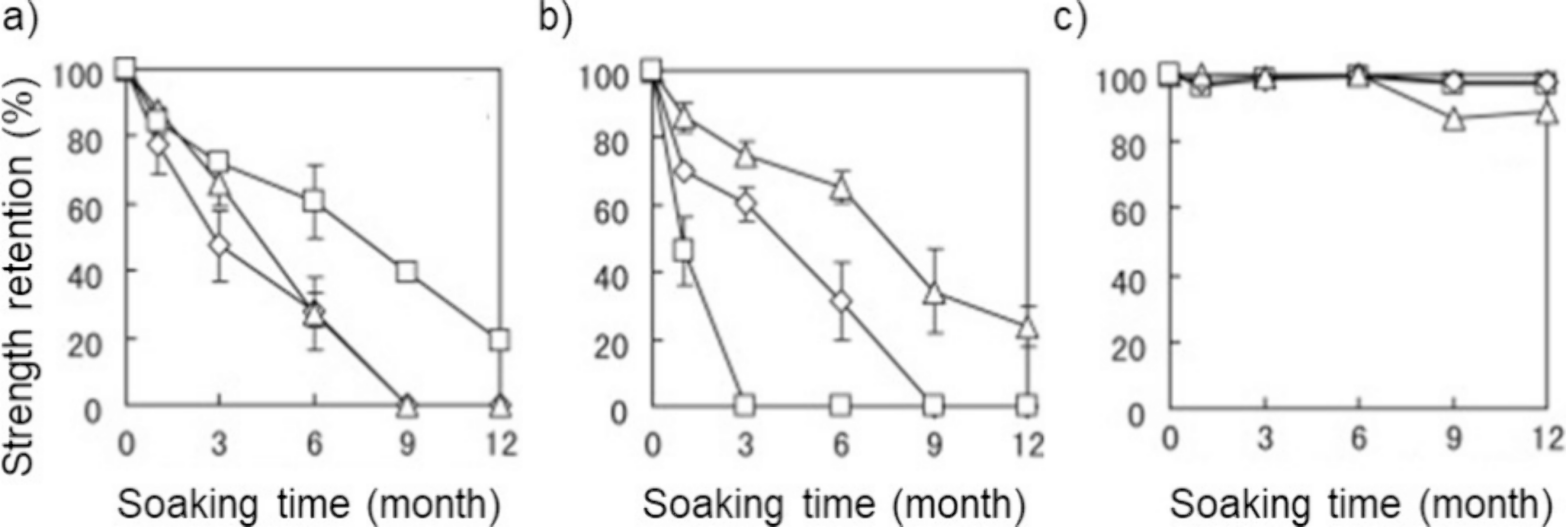
Degradation of (a) PCL, (b) PHBV, and (c) PBS monofilament fibers soaked in deep seawaters in three different locations near Japanese coast. Adapted with permission from ref (56). Copyright 2011 Elsevier.

The abiotic and biotic hydrolysis of polyesters can proceed through surface or bulk mechanisms, and it is typically assisted by initial abiotic processes, such as oxidation or hydrolysis, which decrease the molecular weight and/or increase the hydrophilicity of the material.^55^ It is easier to design materials that degrade under controlled and favorable conditions in industrial compost, but the conditions in natural environments, especially in seawater, are much less favorable for degradation, and even many biodegradable plastics can persist over long periods of time.^28^ The degradation rate can vary from weeks to years depending on the combination of specific material and environment. To make it even more complicated, large differences in the degradation process can be observed even in same type of environment. To illustrate this, Figure 5 presents the strength retention of three aliphatic polyesters that are considered as easily biodegradable, e.g., PCL, PHBV, and PBS. The polymers were soaked in deep sea at three different locations close to the Japanese coastline.^56^ Irrespective of the relatively similar environments, large differences in degradation rate were observed both between the locations and between the different aliphatic polyesters. Of the studied materials, PHBV and PCL demonstrated significant degradation, while the degradation rate still varied largely depending on the specific location. Interestingly, the average temperature at Toyama and Rause, where degradation of PCL proceeded faster was 2 and 5 °C, respectively, while it was slightly higher (10 °C) at Kume, the location where degradation proceeded more slowly. Thereby, temperature could not explain the differences. Unfortunately, pH of the water was not followed, but isolated from all three locations. The bacteria located from Kume had good activity at 25 °C, while bacteria active at 4 °C was found at Toyama and Rausu. This likely explains the differences observed in the degradation rate of PCL at the different locations. PHBV degrading bacteria were not investigated. PBS did not show significant degradation in any of the locations.

The negligible degradation rate of PLA and PBS in seawater, and the higher susceptibility of PCL, PHB, and PHBV to degrade was also confirmed by several other studies.^28,57^ These large differences in the degradation rates clearly demonstrate the sensitive interplay between the prevailing degradation environment and the specific polymer structure, and the sensitivity to small changes in either, making it difficult to guarantee degradation in natural environments. Furthermore, it is clear that structural modification of common biodegradable polyesters is required if reasonable degradation rate in natural environments is targeted.

### 3.2 Modification by More Easily Hydrolyzable Ester Bonds

As a general rule, chemical modifications that increase the hydrophilicity and water uptake of the materials typically also lead to increased hydrolytic degradation rate.^58^ Some ester bonds are also more susceptible to hydrolysis due to, e.g., higher electrophilicity or accessibility to water. Such bonds could be introduced to design rapidly hydrolyzable materials or to tune the degradation rate by providing “weak” points, where degradation can be initiated. Modification by copolymerization,^59^ cross-linking,^60,61^ and introduction of branching^62^ can also be utilized to decrease the degree of crystallinity, as amorphous materials have less tightly packed chains and higher susceptibility to hydrolysis. Many studies and reviews^31,33^ exist on modification of aliphatic polyesters and their susceptibility to degradation by copolymerization, blending, and surface modification with more hydrophilic components,^63−65^ only a few examples are presented here to illustrate different approaches.

#### 3.2.1 Aliphatic Polyesters

Poly(l-lactide) (PLLA) is known to degrade relatively slowly due to the sterical hindrance from the methyl group close to the ester group and the semicrystalline nature and/or crystallization during aging. Some early studies compared the hydrolytic degradation of several PLLA, poly(d,l-lactide) PDLLA and poly(glycolide-*co*-lactide) (PLGA) polymers in phosphate buffer at 37 °C showing surprisingly large differences.^66,67^ The half-life as determined by 50% weight loss decreased from 110 weeks for PLLA to 22, 10, and 10 weeks after introduction of 25% d-lactide units, 50% d-lactide units, and 25% glycolide units, respectively. A polymer containing both l-lactide and d-lactide units in combination with 25% glycolide units, had by far the shortest half-life of only 3 weeks. All of the polymers were initially amorphous, and these large differences were explained by the ability of the degrading polymers and oligomers to crystallize or not during aging. In the case of the PLGA copolymers, the introduction of more hydrophilic units with more accessible ester groups further increased the hydrolytic degradation rate.

**Figure 6:**
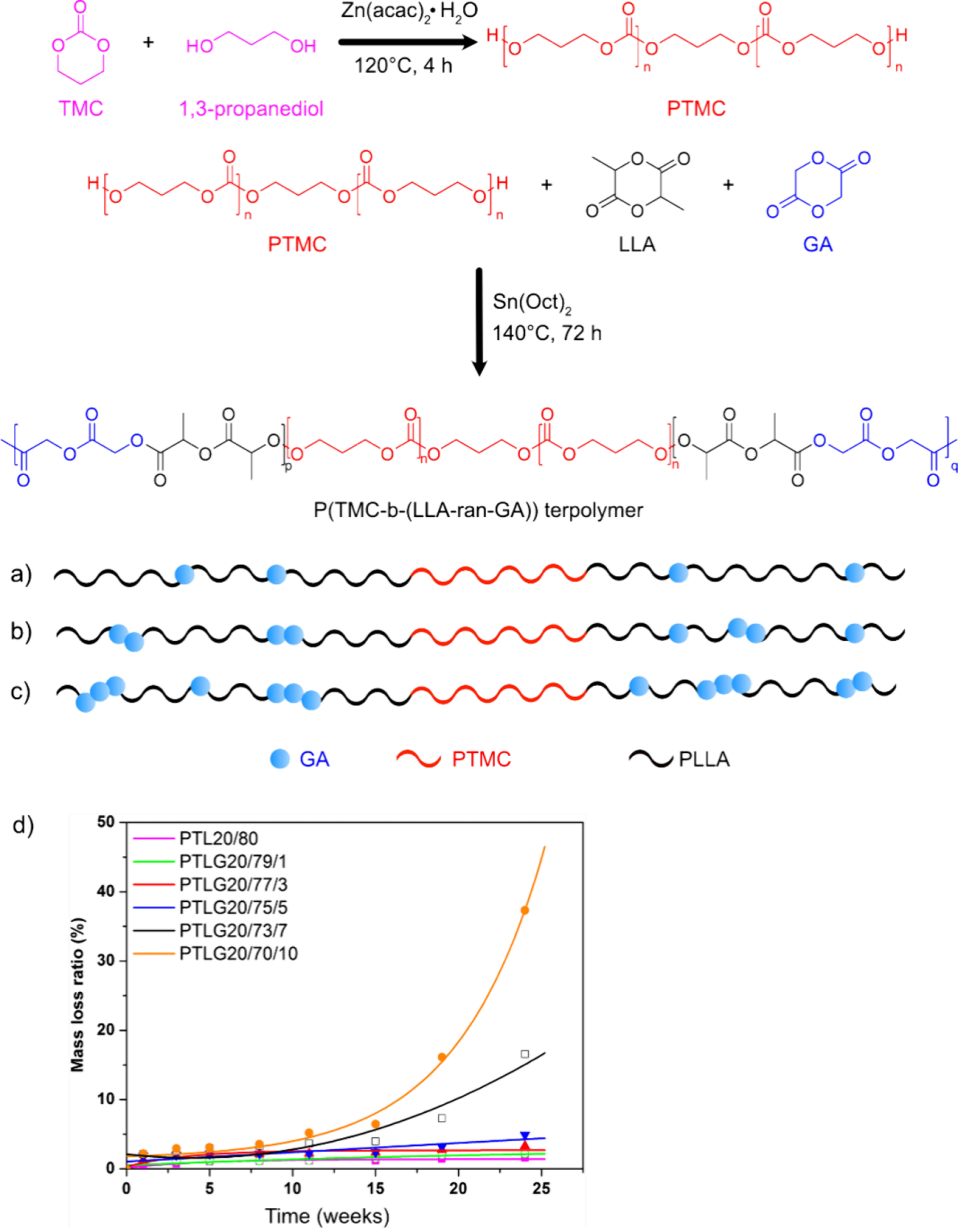
Introduction of different amounts of glycolide (GA) units in poly(trimethylene carbonate-*block*-(l-lactide) (PTL) copolymers to incorporate more hydrolyzable ester bonds in the PLLA blocks to tune and enhance the hydrolytic degradation rate. (a–c) illustrate schematically block copolymers with increasing amount of glycolide units (blue circles) in the PLLA blocks. (d) The weight loss as a function of hydrolytic degradation time for block copolymers with different glycolide contents. The copolymer composition can be read from the sample names, e.g., PTLG 20/70/10 contains 20% carbonate, 70% lactide, and 10% glycolide units. Adapted with permission from ref (73). Copyright 2018 John Wiley and Sons, Ltd.

While randomly incorporated D-and L-units in the polylactide chain rapidly reduce the degree of crystallinity and accelerate the hydrolytic degradation rate, the opposite has been observed for block copolymers, where longer D-and L-blocks in the copolymer chain allow formation of more hydrolytically stable stereocomplex crystals.^68^ The higher hydrolytic stability of the blends of PLLA and poly(d-lactide) (PDLA) due to formation of stereocomplex crystals is also well-known^69,70^ and correlates with the stronger secondary interactions in stereocomplex crystals and higher water barrier properties.^71^ In accordance, studies on polycaprolactone copolymers illustrated the large influence on hydrolytic degradation rate of both the copolymer composition and the arrangement of the comonomers to random, block or multiblock copolymers, contributing to the different distribution of the more easily hydrolyzable ester bonds in the materials.^41,72^ In this context, the triblock copolymer exhibited the largest weight loss and release of monomeric and oligomeric hydrolysis products due to the susceptibility of long hydrophilic 1,5-dioxepan-2-one blocks toward hydrolysis. Figure 6 further illustrates the concept of introducing “weak” more easily hydrolyzable ester bonds to tune the hydrolytic degradation rate of polyesters.^73^ The degradation experiments were performed in phosphate buffer at 37 °C during 24 weeks. The introduction of more easily hydrolyzable ester bonds facilitates weight loss and abiotic hydrolytic breakdown of the polymer and leads to formation of potentially more easily biodegradable low molar mass compounds. However, this should not be considered a proof of ultimate biodegradability, which should always be confirmed by biodegradation experiments under relevant conditions to confirm the mineralization without formation of persistent degradation products.

#### 3.2.2 Aliphatic–Aromatic and Aromatic Polyesters

**Figure 7:**
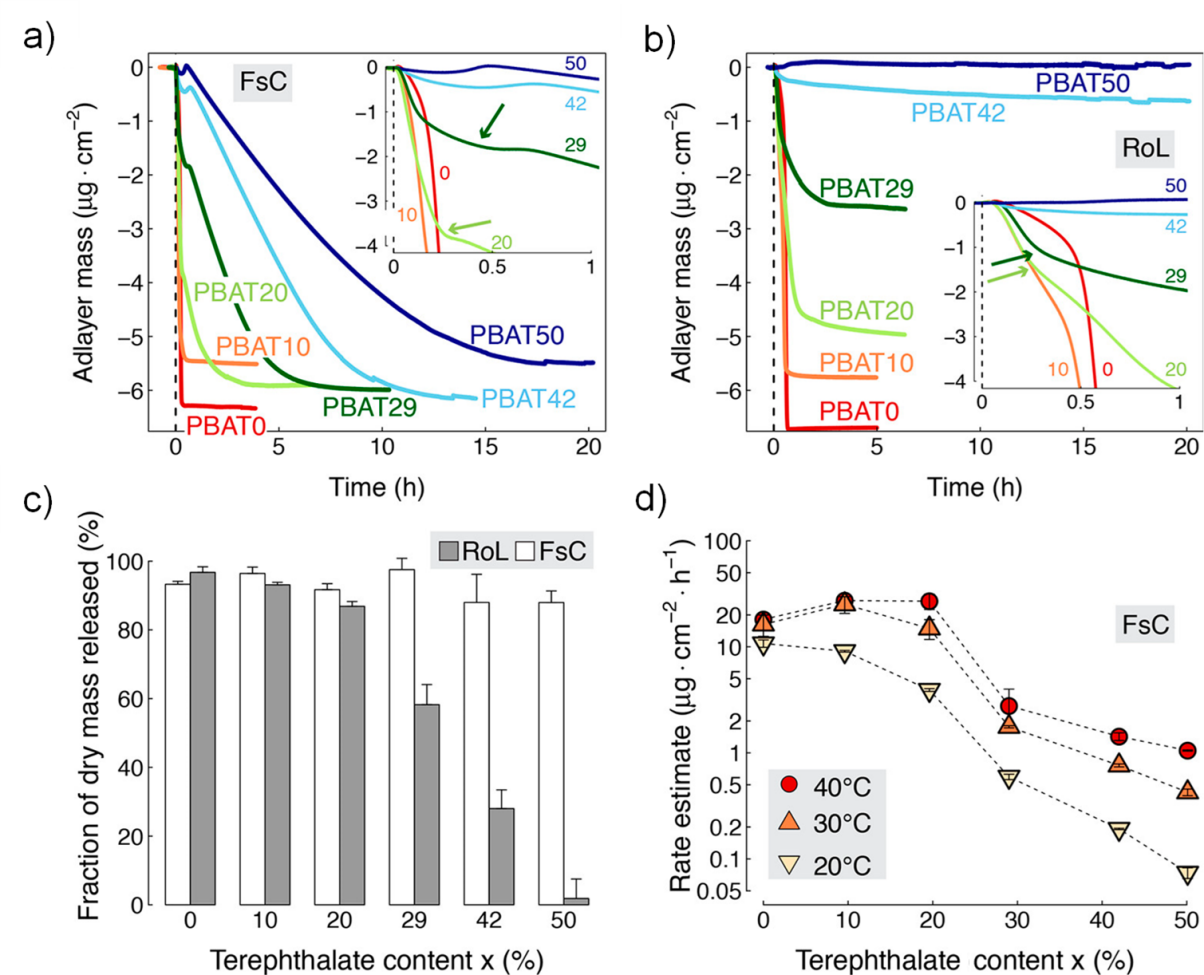
Enzymatic hydrolysis of PBAT thin films with different terephthalate contents by *Fusarium solani* cutinase (FsC) and *Rhizopus oryzae* lipase (RoL) at pH 6. The degradation was followed by quartz crystal microbalance with dissipation monitoring (QCM-D). Changes in the adlayer mass during the hydrolysis catalyzed by (a) FsC and (b) RoL at 30 °C. (c) Fraction (%) of dry polyester that was released during the hydrolysis experiments. (d) FsC catalyzed hydrolysis rate at three different temperatures. Reproduced with permission from ref (79). Copyright 2017 American Chemical Society.

Aromatic polyesters, such as PET, are typically not sensitive to low temperature hydrolysis or biodegradation without pretreatment to, e.g., reduce the molar mass.^74^ However, an interesting commercial and biodegradable aliphatic–aromatic polyester, PBAT, has been developed to bridge aliphatic and aromatic polyesters.^75^ In PBAT, the separation of the aromatic segments with aliphatic units provides regions where initial degradation can take place, releasing oligomers that, due to their low molecular size, are more easily accessible for further biodegradation, and more than 90% mineralization has been shown during simulated composting experiments.^76^ At the same time, a sufficiently high number of aromatic units were left to give the polymer good physical properties.^77,78^ Quartz crystal microbalance experiments clearly illustrated both the influence of terephthalate content and the specific enzyme on the degradation rate of PBAT polymers.^79^Figure 7 shows how the enzymatic hydrolysis rate of PBAT decreases as the terephthalate content in the copolymer increases. At the same time, the degradation rate is highly influenced by the type of enzyme and temperature. The degradation rate and properties can be further modulated by copolymerization with additional monomers. As an example, copolymerization with polyglycolide prepolymer led to materials with improved mechanical and barrier properties and faster degradation rate in water.^80^ The material properties of PBAT also depend on the aliphatic/aromatic ratio. Commercial PBAT is a flexible material with properties similar to low density polyethylene (LDPE). The biodegradability thus comes at the cost of some mechanical and barrier property reduction compared to aromatic polyesters, such as PET. PBAT can be used to replace LDPE in applications, where biodegradability is a favorable property, such as mulch films, compostable bags, and products contaminated by organic matter.

**Figure 8:**
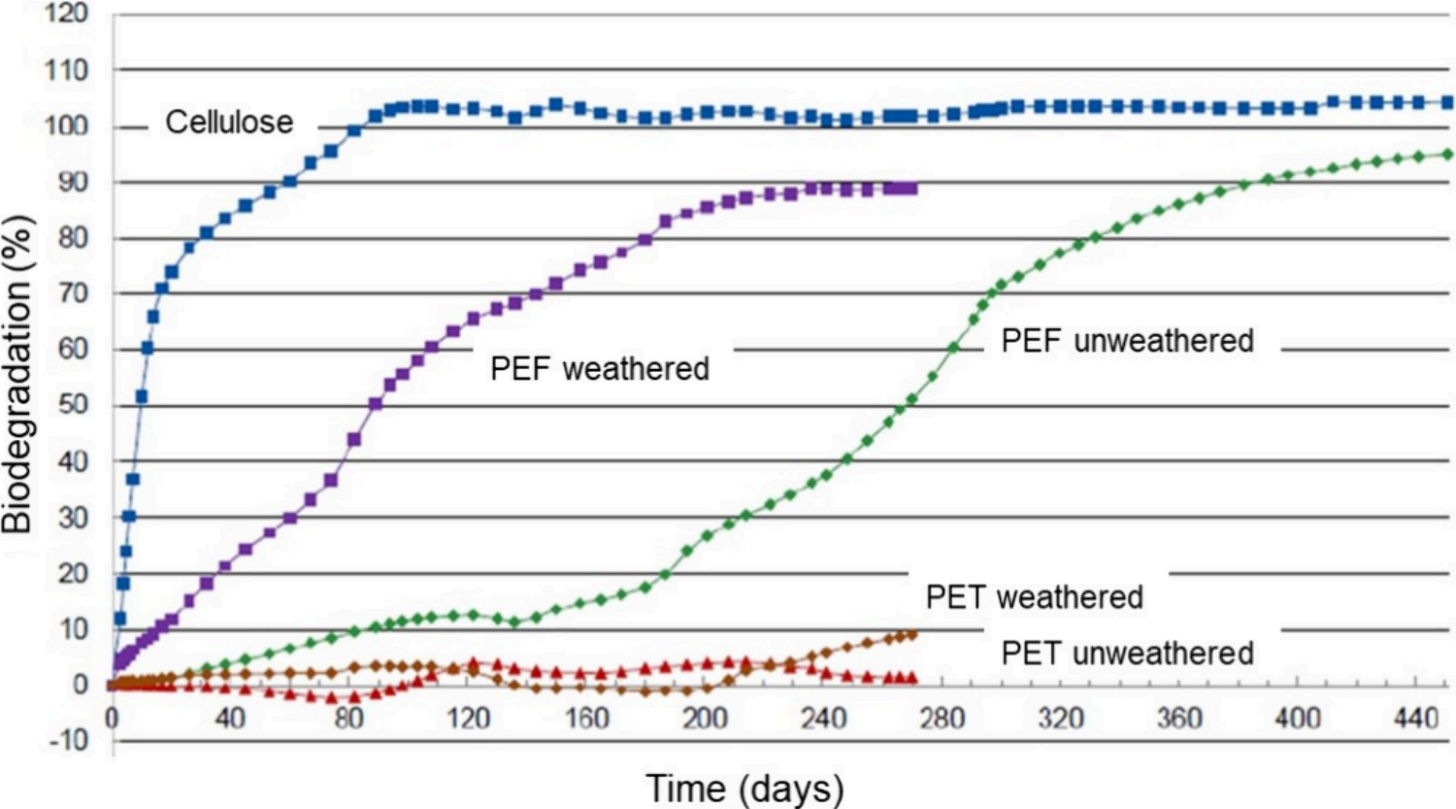
Biodegradation of weathered and unweathered PEF and PET under simulated composting conditions at 58 °C. Cellulose was included as biodegradable reference, and biodegradation was quantified by measuring the production of CO_2_. Adapted with permission from ref (83). Copyright 2022 The Authors. CC-BY 4.0. Published 2022 MPDI.

Poly(ethylene furanoate) (PEF) is an emerging commercial aromatic polyester. It is anticipated to have high potential as biobased replacement material for PET, as it has similar and even better mechanical and barrier properties and lower environmental impact in comparison to PET.^81^ PEF can also be chemically and mechanically recycled similar to PET. Furthermore, while recycling polymer blends typically leads to deterioration of properties, it was shown that low amounts of PEF can even improve the properties of mechanically recycled PET.^82^ However, PEF is not rapidly biodegradable and does not fulfill the requirements to be classified as industrially compostable plastic (requiring >90% biodegradation to CO_2_ during 180 days).^83^ Still replacing the terephthalate units in PET by furanoate unit in PEF significantly increases the susceptibility to biodegradation, as a recent study showed >90% conversion of PEF to CO_2_ after 385 days in simulated industrial compost (Figure 8). After weathering, the 90% mineralization was reached already after 240 days. After the same time period, the biodegradation degree of weathered PET was 10%, while biodegradation of PET remained negligible. Similar to PBAT, the biodegradability of PEF can be increased by copolymerization.^84,85^

Another approach was recently developed for obtainment of more readily degradable aromatic or aromatic–aliphatic polyesters by incorporation of salicylic acid, an aromatic hydroxyacid, into the polymer structure.^86,87^ While the aromatic ring contributed to attractive thermal, mechanical, and oxygen barrier properties, the more acidic carboxyl and hydroxyl groups contributed to significantly higher hydrolytic degradation rate in different aqueous environments. As an example, salicylic glycolide and salicylic lactide were ring-opening polymerized to corresponding polyesters, which were shown to completely degrade to water-soluble degradation products within 20–40 days in phosphate buffer (pH 7.4) and artificial seawater (pH 8.0) at 50 °C, while commercial PLA and PET showed no weight loss during 100 days in artificial seawater.^86^ In phosphate buffer, the weight loss of PLA started around 60 days. The tested spherical samples, with ∼2 mm diameter, were prepared by compression molding, followed by quenching with cold water. The results are interesting, but the temperature used for testing is significantly higher compared to 30 °C recommended in the standard test method for determining aerobic biodegradation of plastic materials in the marine environment (D6691-17) or the average temperature estimated for ocean surface water (17 °C) and sea floor (4 °C). The results presented in supporting information also show that the degradation rate in phosphate buffer decreases significantly when the temperature is decreased to 40 °C and almost no degradation takes place at room temperature (23–27 °C).

**Figure 9:**
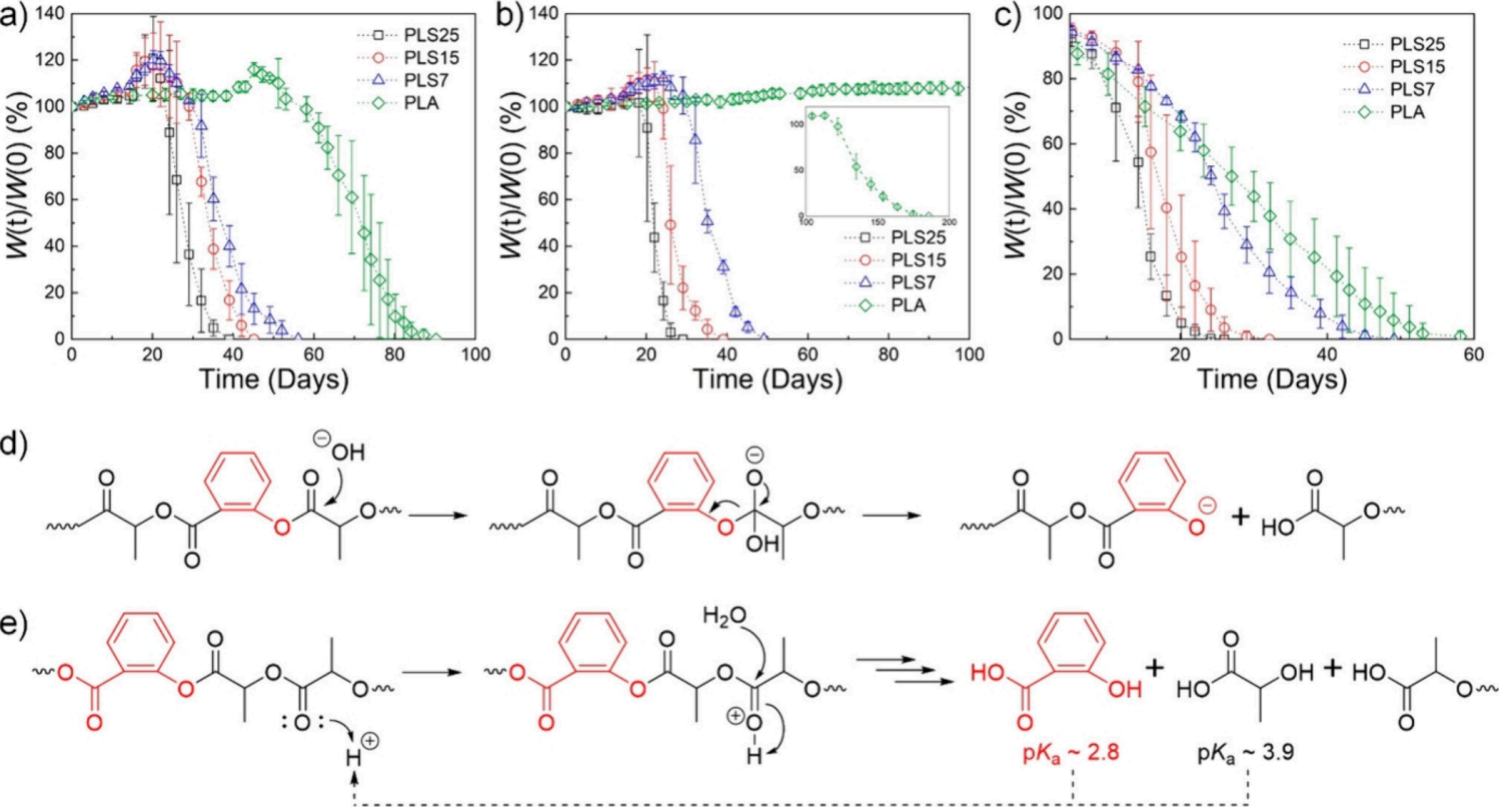
Weight loss of PLA, PLS7, PLS15, and PLS25 during hydrolytic aging at 50 °C in (a) 1 M pH 7.4 phosphate buffer, (b) pH 8.1 seawater, and (c) 0.1 M aqueous NaOH. Proposed mechanism for salicylate-facilitated degradation under (d) basic and (e) acidic conditions. Reproduced with permission from ref (88). Copyright 2021 American Chemical Society.

Industrially viable transesterification during melt extrusion was utilized for introduction of salicylate units as weak linkages in commercial polymers, such as PLA. Through this approach, original material properties (thermal, mechanical, and oxygen barrier) were retained, while significantly increased hydrolytic degradation rates were demonstrated.^88^ Furthermore, the degradation rate could be easily tuned by changing the amount of salicylate units incorporated in the PLA chain. As an example, 100% weight loss in phosphate buffer at 50 °C was recorded within 40–55 days for PLA modified with different amounts of salicylate units (PLS7, PLS15, and PLS25), while it took more than 90 days for PLA under similar conditions (Figure 9). Even larger differences were observed during aging in seawater. By performing experiments at different temperatures and by utilizing the Arrhenius equation, the authors estimated that it would take 2.8 years for PLS25 with the highest salicylate content to completely degrade in phosphate buffer under ambient conditions, while it would take 5.5 years for PLA. This was deduced to the easier cleavage of salicylate units and the catalytic effect of more acidic salicylic acid units with p*K*_a_ ∼ 2.8 compared to p*K*_a_ ∼ 3.9 for lactic acid.

## 4 Increasing Circularity by Neighboring Heteroatoms

While the ester bond is well-known for its reversible behavior and susceptibility to hydrolysis, the ester exchange and hydrolysis rates^89^ are typically significantly lower as compared to other dynamic bonds such as imines and disulfides. It could also require external catalysts such as Lewis acids^90^ and/or elevated temperatures.^91^ The hydrolysis accelerating effects of free hydroxyl and carboxyl groups are well-known from early hydrolysis experiments, where the presence of monomer residuals^92^ or large amounts of acidic degradation products^93^ was shown to have autocatalytic effect on the hydrolysis rate. In correlation, the end-capping of hydroxyl groups at the chain ends significantly reduced the susceptibility to hydrolysis.^94,95^ This is explained by lower water uptake as well as change in degradation mechanism from chain-end scission to merely random chain scission.^96^ In line with this, although thin or porous specimens could be expected to degrade faster due to larger surface area, in many cases the opposite has been observed due to the autocatalytic influence of the formed acidic hydrolysis products. This is especially significant in the case of large specimens, as the formed hydrolysis products are trapped inside and catalyze the hydrolysis process inside the specimen.^97^

**Figure 10:**
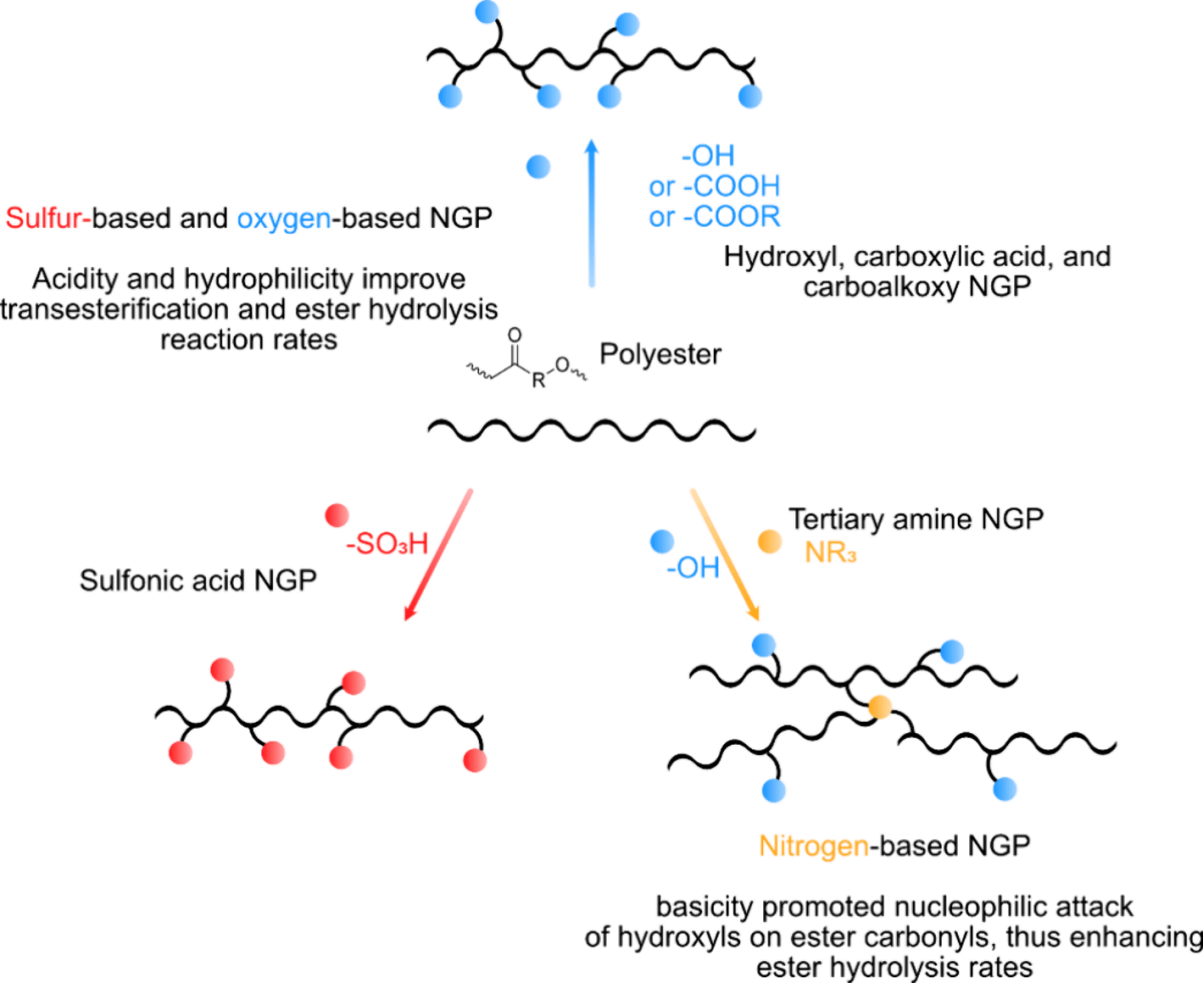
Schematic presentation of polyesters modified by neighboring oxygen, sulfur, and nitrogen containing groups and the potential circularity promoting influences.

Similar to this, the dynamic efficiency of the ester bond can be improved by nearby basic and nucleophilic heteroatoms such as oxygen, nitrogen, and sulfur.^98^ Such heteroatoms can be incorporated in polymer materials in the form of functional groups (e.g., carboxyl, hydroxyl, amine) and are thereby expected to act as internal catalysts both for chemical exchange reactions and hydrolysis (Figure 10). Depending on the type of modification, the monomer with extra functionality could be added already during the polymer synthesis or it could be incorporated to the polymer chain by, e.g., transesterification. Utilization of reactive extrusion could give an opportunity to tailor-make existing commercial materials to degradation in targeted end-of-life environment. The utilization of nearby functional groups for enhancing the rate of exchange reactions is also known as neighboring group participation (NGP).^98^

### 4.1 Polyesters with Neighboring Nitrogen Atoms

The placement of neighboring amines to enhance ester exchange reactions (i.e., transesterification) has mainly been used in epoxy-ester based networks for introducing mechanical recyclability. For example, transesterification of PET with polyol containing five hydroxyl groups and a tertiary amine broke the polyester chains and incorporated tertiary amine moieties and reactive hydroxyl groups.^99^ These hydroxyl groups were then available for reaction with epoxy groups to form an ether and new hydroxyl groups. Due to the synergetic catalyzing effect of hydroxyl and tertiary amine groups, the resulting networks were fully reprocessable by, e.g., hot-pressing and extrusion. In another approach, primary and secondary amines underwent reaction with two diepoxy molecules, to form diepoxy crosslinkers with a tertiary amine and hydroxyl group.^100^ The obtained crosslinkers were then further reacted with citric acid monohydride and succinic acid, yielding an epoxy-ester based network. Due to the neighboring nitrogen atoms the observed stress relaxation times were similar to those observed in the presence of external catalysts for the ester exchange reaction.

The presence of tertiary amines as pendant groups instead of inherent components of the polymer network also enhanced the transesterification reactions rates. The presence of both tertiary amines and hydroxyl pendent groups yielded polyester thermosets with stress relaxation times reduced by a factor of 20 in comparison to neat polyester thermosets, thus imparting good reprocessability.^101^ The effect of tertiary amines on transesterification reactions in phthalate monoester-based networks was also studied.^102^ Stress relaxation experiments at 160 °C showed that relaxation times decreased from 515 s for the network lacking tertiary amines down to 1.1 s for the network containing the highest amount of tertiary amines. This considerable decrease in stress relaxation time showed that the presence of tertiary amines alone significantly enhanced transesterification reactions. These results demonstrate that the presence of tertiary amines have a beneficial effect on transesterification rates, thus imparting thermal reprocessability.

Studies on the ability of tertiary amines to facilitate chemical recyclability have been limited. In this regard, incorporation of tertiary amines as internal catalysts in epoxy-ester networks was evaluated by utilizing the hydroxylamine-based compound triethanolamine (TEOA).^103^ During the formation of the thermoset, the hydroxyl groups of TEOA initially reacted with an anhydride to form a carboxylic acid group, which was then available to react with an epoxy group to form an ester and a β-hydroxyester linkage. The resulting network, containing ester, triamine, and hydroxyl groups, exhibited brittleness (elongation at break 8–9%) but good mechanical strength (85–94 MPa) and high *T*_g_ ∼ 135 °C. *T*_g_ was, thereby, comparable and tensile and impact strength even higher than those of conventional anhydride cured bisphenol A (BPA) epoxy thermosets, which could enable use in high temperature applications and as structural components. At temperatures ranging between 170 and 200 °C, moderate to fast stress relaxation times (19 460 to 4200 s) were observed depending on TEOA content, indicating the occurrence of exchange reactions such as transesterification reactions. With an increasing TEOA content, faster stress relaxation times were observed, which was attributed to the synergetic catalyzing effect of the hydroxyl and tertiary amine functional groups.

By focusing on the chemical recycling, the obtained resin was hydrolyzed in an aqueous solution of 1.5 wt % phosphotungstic acid at 190 °C for 5 h. After the reaction, a degraded oligomeric residue was obtained with a molecular weight of 2200 g/mol. The product was fully soluble in acetone, indicating significant degradation. It should be mentioned that without the use of phosphotungstic acid, no degradation was observed. Fourier transform infrared (FTIR) spectroscopy showed that the degraded residue contained abundant −OH and −COOH groups, suggesting that the degradation mainly occurred at the tertiary amine and ester moieties. This degradation pathway can be explained by the basic nature of the tertiary amine assisting the nucleophilic attack of hydroxyls/water on the carbonyl carbons (i.e., ester linkages) resulting in the cleavage of ester bonds.^104^ Due to the −OH and −COOH rich hydrolysis products, the degraded oligomers could be blended with fresh resin and recured to form a new thermoset through transesterification reactions, promoted by the presence of hydroxyl and tertiary amine groups. The storage modulus and glass transition temperature (*T*_g_) of the new thermoset were similar to those of the original thermoset, indicating successful chemical recyclability and reuse of the recycled chemicals in equal value application.

Above studies investigated systems with both hydroxyl and tertiary amine groups and reported synergistic catalyzing effects on transesterification reactions. However, comparisons with resins and thermosets containing only tertiary amines or hydroxyl groups were not mentioned. Nevertheless, the catalytic effect of hydroxyl and tertiary amine groups on enhancing both transesterification reaction rates and ester hydrolysis were clearly highlighted. There is high potential in this approach, but more research is required to establish the influence of nitrogen containing neighboring groups on chemical recyclability, hydrolytic degradation, and biodegradation of polyesters. The research should also be expanded to linear polyesters to evaluate the influence on neighboring groups on mechanical properties and potentials risks of premature degradation during, e.g., thermal processing.

### 4.2 Polyesters with Neighboring Oxygen Atoms

Recently, oxygen containing functional groups, like hydroxyl, carboxyl, and carboalkoxy, have been introduced into polyesters to enhance circularity. The weak acidic and hydrophilic nature of such oxygen-containing NGP is expected to promote the ester exchange reactions and ester cleavage. For example, a comparative study between conventional polyesters, PCL and poly(11-hydroxyundecanoate) (PHU), and a polyester containing hydroxyl pendant groups (PEUA) showed beneficial effects of hydroxyl pendant groups on the polyester degradability in both hydrolytic and enzymatic media.^105^ Under accelerated hydrolytic conditions at temperature significantly above *T*_g_ of the studied polymers (incubation in aqueous media with pH 2.0 at 45 °C for 10 weeks), the number-average molar mass (*M*_n_) of PEUA decreased from 8400 g/mol before hydrolytic incubation to 3700 g/mol after incubation. The weight loss after 80 days was only a few percent, indicating that the pendant hydroxyl groups could catalyze the hydrolytic degradation in bulk, but the molar mass reduction was still not enough to form water-soluble oligomers. It would have been interesting to follow the process over a prolonged time period to confirm the further hydrolysis of the remaining low molar mass polymer. Conversely, no decrease in molar mass was observed for PCL and PHU, while the observed weight loss was somewhat larger compared to PEAU. This in turn supports surface erosion, releasing water-soluble degradation products without significant influence on molar mass. Considering also that the original molar mass of PEAU was twice as high as that of PCL and PHU, this indicates that the presence of acidic and hydrophilic −OH groups in PEUA could enhance the susceptibility to hydrolysis. The introduced pendant groups also increased the hydrophilicity of the materials and decreased the degree of crystallinity, which further facilitates faster hydrolysis. Similar results were obtained for enzymatic degradation (incubation for 10 days in pH 7.4 at 37 °C with porcine pancreatic lipase). However, enzymatic degradation was more limited as compared to hydrolytic degradation due to the difficulty of the enzyme to penetrate the polymer system.

In another study, a series of hydroxy-functional copolyesters was synthesized from adipic acid, 1,8-octanediol, and glycerol, varying the hydroxyl content by increasing the 1,8-octanediol:glycerol ratio.^106^*In vitro* degradation was evaluated in phosphate buffer (pH ∼ 7.4 at 37 °C) for a predetermined time. After 7 days, the observed weight loss ranged from 20% for the polymers with adipic acid:1,8-octanediol:glycerol ratio of 1:0.8:0.2 to 55% for polymers with higher glycerol content, i.e., adipic acid:1,8-octanediol:glycerol ratio of 1:0.5:0.5. These results support that increasing the hydroxyl content leads to faster biodegradation rates. This can be attributed to the combined effect of increased hydrophilicity, decreased degree of crystallinity, as well as the catalyzing effect of free hydroxyl groups, all promoting the cleavage of ester bonds under hydrolytic conditions. Neither of the above studies investigated the influence of neighboring groups on the mechanical properties of the materials, which is crucial from an application perspective.

Another promising oxygen-based NGP is carboxylic acid. Early works showed that the introduction of neighboring carboxylic acid groups in phthalate monomethyl esters rapidly increased the hydrolysis rates up to 10-fold under mildly acidic to neutral conditions (pH 4–7), as compared to those of corresponding benzoate esters lacking neighboring carboxylic acid groups.^107,108^ The relative proximity of acidic carboxyl groups near ester bonds promotes ester bond cleavage as well as ester exchange reactions. More recently, this carboxylic acid catalyzed transesterification rate enhancement was utilized in the design of phthalate- and polyester-based covalent adaptable networks to avoid the need of external catalysts to initiate ester bond cleavage and transesterification reactions.^109^ The dynamic networks were recyclable for multiple cycles using a solvent-based recycling approach. Analysis showed that the obtained precipitate consisted of prepolymer network fragments, which could be recured by heating at 100 °C for 4 h under N_2_ atmosphere. Even after multiple recycling steps (i.e., dissolving and heating), chemical, thermal, and mechanical properties of the material were not affected, indicating promising closed-loop recyclability.

**Figure 11:**
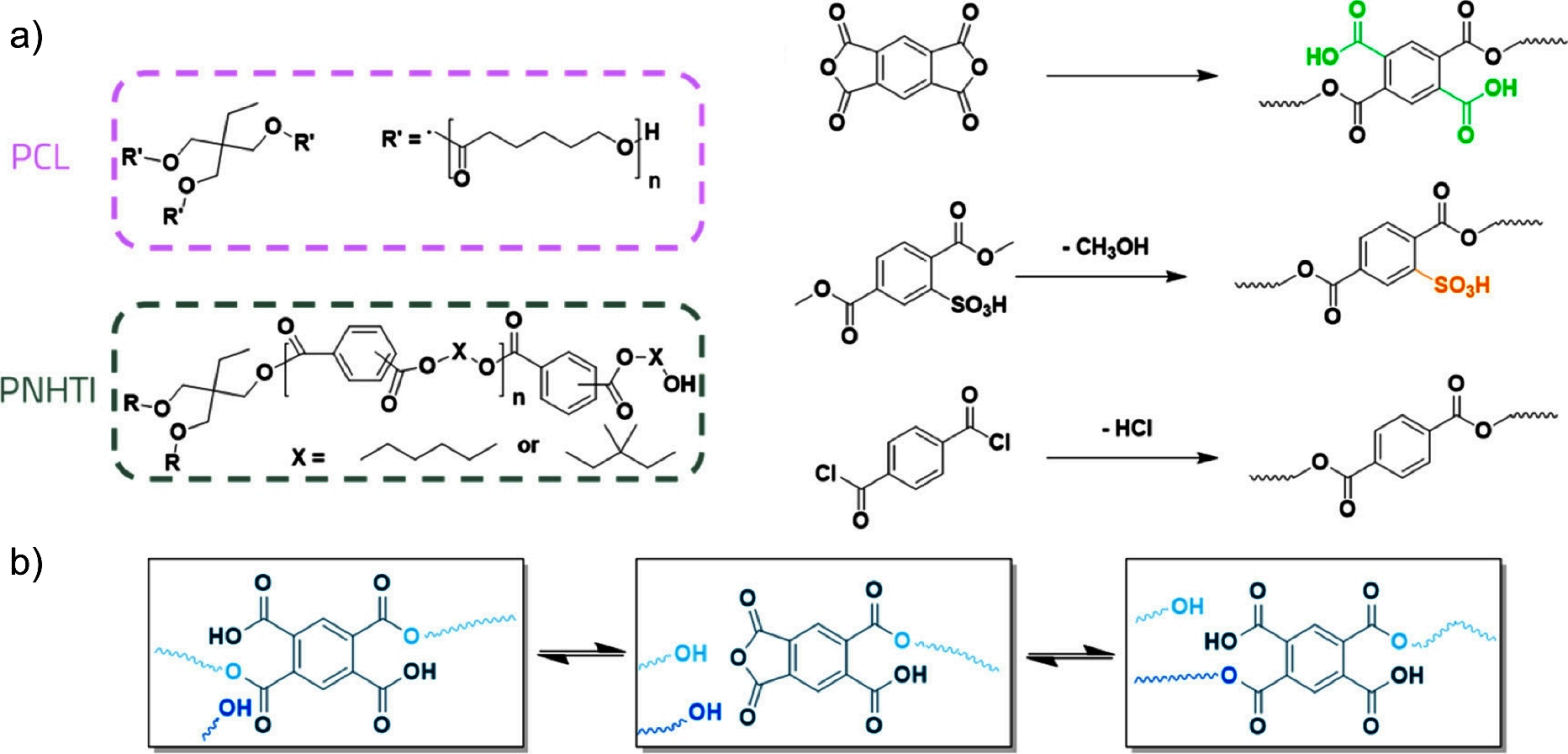
(a) Network formation with internal carboxylic and sulfonic acid groups and (b) bond rearrangements in networks with neighboring carboxylic acid group. Reproduced with permission from ref (110). Copyright 2020 American Chemical Society.

Despite the promising results of neighboring acid groups being able to enhance hydrolysis rates of polyesters, current carboxylic acid-ester NGP research has mainly focused on mechanical recycling. For example, dynamic polyester networks were prepared by a reaction between branched polyesters containing −OH end groups with pyromellitic dianhydride or 2,5-bis(methoxycarbonyl) benzenesulfonic acid.^110^ The resulting polyesters contained, respectively, −COOH or −SO_3_H functional neighboring groups in the *ortho* position to every formed ester linkage (Figure 11). The stress relaxation experiments showed that both networks containing −COOH or −SO_3_H groups exhibited significantly faster stress relaxation in comparison to a conventional reference network lacking any neighboring groups but containing 0.5 mol % of external transesterification catalyst Zn(Acac)_2_. This indicates that the presence of such neighboring groups significantly enhances the dynamic behavior of polyester based networks. Interestingly, observed stress relaxation of −SO_3_H containing polyester networks was 5 times faster than the stress relaxation of networks with −COOH groups. The presence of the sulfur groups may thus further enhance ester exchange reactions. This can be explained by the much higher acidity of sulfonic acids as compared to carboxylic acids. Nevertheless, the acidic nature of carboxylic acid contributes to faster ester exchange reactions, making them promising NGP in polyester-based systems by imparting these materials with potentially improved mechanical and chemical recyclability.

Interestingly, promising ester exchange enhancement was also achieved by replacing the hydrogen in the carboxylic −OH group with an alkyl chain to yield a carboalkoxy group despite the lower acidic nature. For example, chemically recyclable carbomethoxylated polyvalerolactone (PCMVL) was obtained via ring-opening transesterification polymerization of the renewable monomer 4-carbomethoxyvalerolactone (CMVL).^111^ The resulting semicrystalline polyester, containing a carbomethoxy group in its repeating unit, exhibited a *T*_g_ of −18 °C and two distinct melting temperatures at 68 and 86 °C, respectively. Chemical recyclability of PCMVL was tested by using depolymerization methods via two different pathways. First, when PCMVL was heated at 150 °C in the presence of tin octanoate (SnOct_2_) as a catalyst, near complete degradation via transesterification was achieved to fully recover the initial cyclic monomer, CMVL, with a yield of 87%. Alternatively, in the presence of 1,8-diazabicyclo[5.4.0]undec-7-ene, the polymer chain was cleaved through elimination processes into smaller oligomeric fragments.

Although these promising results indicate the potential chemical recyclability of carboxymethoxylated polyvalerolactone, no reference studies were performed with polyvalerolactone lacking carbomethoxy pendant groups. However, following the degradation process with ^1^H NMR analysis showed that the cleavage and elimination reactions along the main chain preferably occurred in the vicinity of a proximal carbomethoxy group, suggesting that the presence of the carboxylate ion does enhance chemical recyclability of polyvalerolactone. In another study, the influence of the alkyl side chain length on the hydrolytic degradation rates of carboalkoxylated polyvalerolactones was evaluated.^112^ Similar to the previous study, the hydrolytic degradability of a series of 4-carboxylated polyvalerolactones with methyl, ethyl, propyl, and butyl alkoxy side chains was evaluated in basic (0.1 M NaOH), acidic (0.1 M HCl), and neutral environments at 80 °C. After 13 days, all polyesters were fully degraded under both acidic and basic conditions. However, it was observed that the hydrolysis rates of the polyesters significantly depended on the alkyl side chain and the degradation rate decreased with increasing alkyl side chain length under both acidic and basic conditions. It was suspected that this was caused by the longer alkyl chains inducing increased hydrophobicity, thus reducing the water uptake and susceptibility of the polyesters to hydrolytic degradation.

In summary, oxygen based NGP in the form of hydroxyl, carboxyl, and carboalkoxy functional groups has the potential to impart polyesters with improved chemical recyclability and faster hydrolytic degradation. The presence of such functional groups increases hydrophilicity as well as acidity that can enhance ester bond cleavage and ester exchange reaction rates. This is further facilitated by typically lower degree of crystallinity after introduction of neighboring groups. Research should be expanded to establish that these accelerating effects persist under less accelerated hydrolysis and biodegradation conditions.

### 4.3 Polyesters with Neighboring Sulfur Atoms

Sulfur-atom based neighboring group participation, mainly in the form of sulfonic acid (−SO_3_H), to increase the rate of ester bond exchange and cleavage reactions has recently gained attention. As mentioned above, in comparison to oxygen-based −COOH carboxyl groups, the sulfur-containing −SO_3_H has significantly higher capability to accelerate ester exchange reactions.^110^ Such neighboring acid groups accelerate thermally induced transesterification reactions by reacting intermolecularly with ester bonds to form an anhydride intermediate, thus imparting polyesters with improved mechanical recyclability.^110^ However, due to the high acidity and hydrophilicity of sulfur (ions), sulfur-based neighboring group participation may also accelerate ester hydrolysis rates and impart polyesters with improved chemical recyclability. As an example, sulfonated PBS showed significantly increased hydrolysis rate in pH 12 aqueous alkali solution.^113^ The water uptake of the sulfonated PBS materials increased linearly with the concentration of ionic groups. The same trend was observed for the hydrolytic degradation rate as the approximately 15% weight loss for PBS after 30 days gradually increased to approximately 95% for the material with the highest concentration of ionic groups.

Similarly, a chemically recyclable polyethylene-like polyester containing low amounts of ionic sulfonate groups was synthesized via polycondensation reactions at 150 °C and under reduced pressure by using octadecane-1,18-dicarboxylic, octadecane-1,18-diol, and dimethyl sulfosuccinic acid (HMSS) as monomers.^114^ The presence of low amounts (0.8 mol %) of HMSS led to polyesters containing a low content of −SO_3_-H pendant groups. Interestingly, the presence of pendant sulfonic acid groups did not significantly influence the thermal properties. On the other hand, an increase in sulfonic acid content led to an increase in stiffness as compared to a polyester lacking any neighboring groups. The susceptibility of the sulfonic acid containing polyesters to hydrolysis was evaluated by immersing the polyester in water for 10–12 weeks. During this time period, the weight gain upon water absorption as well as the degree of polymerization (DP_*n*_) of sulfonic acid containing polyesters was compared to the nonfunctionalized polyester. The presence of sulfonic acid groups significantly increased the water uptake of the polyesters, indicating its increased hydrophilicity. As a result, the ester bonds were significantly more exposed to water, which is known to be important for the hydrolysis rate. This was further demonstrated by the observed decrease in DP_*n*_ for the sulfonic acid containing polyesters, showing a significant decrease in DP_*n*_ of 50–60%. Considering that no significant change in water uptake and DP_*n*_ was observed for the polyester without any sulfonic acid groups, it was concluded that the presence of sulfonic acid functionalities significantly increased the susceptibility to hydrolysis.

To test the chemical recyclability of these sulfonic acid-containing polyesters, depolymerization experiments via solvolysis in methanol were carried out at 150 °C. After cooling down to room temperature, a solid residue was obtained. The yield after purification was 80%, and the product consisted of a 1:0.99 mixture of the initial diol and diacid monomers. It was expected that the initial small amount (0.8 mol %) of the HMSS monomer is removed during the recrystallization process. Nevertheless, these results show that complete ester cleavage of the sulfonic acid containing polyesters can be achieved, yielding polyesters that are fully chemically recyclable. Although research is still limited, sulfonic acid neighboring group participation for the enhancement of transesterification rates and ester hydrolysis has proven to be very effective, yielding polyesters with higher susceptibility to hydrolytic degradation and improved mechanical and chemical recyclability.

The utilization of neighboring heteroatoms to facilitate the recyclability and especially the hydrolysis and biodegradation rate of polyesters is still in its infancy. Above studies indicate high potential for tuning the degradation rate for different end-of-life environments. However, the amount of research is scattered and limited and the experimental conditions, including degradation testing varied a lot with respect to pH, temperature, and time. More research is needed to establish structure–degradation relationships by more systematic variation of type and degree of neighboring groups, studies on hydrolytic degradation under less accelerated standardized condition and biodegradation studies under different environmental conditions by using standardized test protocols. Even more so, the influence of these modification on the thermal and mechanical properties of the materials and the stability during thermal processing needs to be investigated to ensure performance during service. Systematic evaluation of the influence of type and concentration of neighboring groups on processability, material properties, and degradation rate could release the full potential of this approach.

## 5 Increasing Circularity by Double Dynamic Structures

Esterification is a classical equilibrium reaction and ester-bond is well-known for its reversibility. However, as a concept the dynamic covalent chemistry (DCC) was first presented in 2002.^115^ Some years later, the terms covalent adaptative network (CAN)^116^ and vitrimer^117^ were introduced, both referring to crosslinked polymer networks exhibiting reversible bonds. Covalent bonds can be classified as dynamic if they can reversibly form and break under equilibrium control and under impact of a specific stimulus.^118,119^ A multitude of different bonds, such as esters, disulfides, imines, acetals, urethanes, and boronic esters, have been employed with or without additional catalyst for the design of more circular polymer materials.^120^ In addition to heat sensitive bonds for design of mechanically recyclable thermosets, e.g., photoreversible bonds and pH sensitive DCCs have been developed, potentially enabling chemical recycling of polymers under mild conditions.

**Figure 12:**
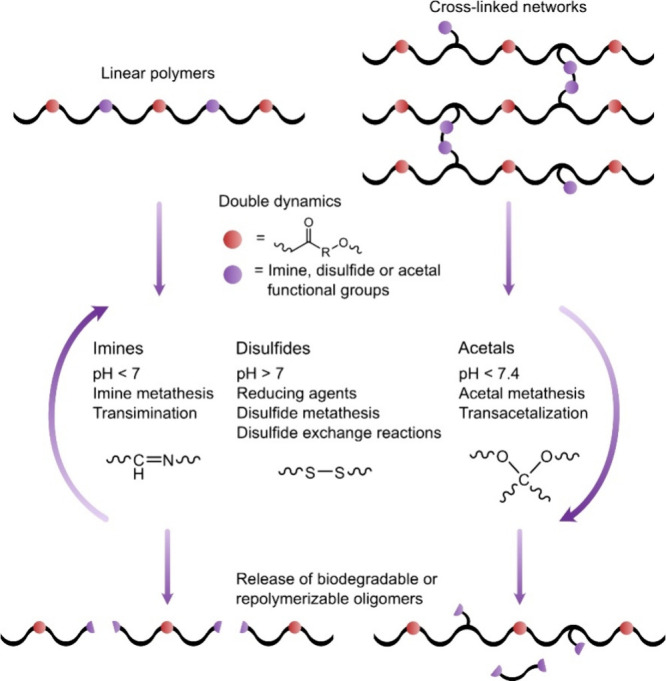
Schematic over linear and crosslinked double dynamic polyesters, where the second dynamic bond, such as imine, disulfide, or acetal, facilitates chemical recyclability and biodegradability by enabling release of oligomers under facile conditions.

An interesting option could also be the development of linear polymers that are sensitive to different environmental triggers to release oligomers that are more easily further biodegraded compared to the original high molar mass polymers. The release of oligomers that can be directly repolymerized to polymer materials with original properties is also of interest.^121^ Combining ester bonds with second more easily reversed dynamic bond to form double dynamics is an attractive possibility to tailor and facilitate the circularity of the materials under mild conditions (Figure 12). The second bond and its abundance can be selected to tune the properties for specific application and the reversibility in specific end-of-life environment. The double dynamics that could facilitate both chemical recycling and biodegradation include, e.g., polyester-imines, polyester-disulfides, polyester-acetals, and polyesters with photoreversible bonds.

### 5.1 Polyester-Imines

In the wide spectrum of the dynamic covalent bonds, imine-bond, also called Schiff base, is commonly employed to impart recyclability and self-healing properties to polymers.^122^ Imines are obtained through a click chemistry reaction in which an active carbonyl group is condensed with a primary amine or more seldom with a ketone to form C=N bonds.^123^ The often excellent recyclability of Schiff base polymers originates from the capacity of the imine bonds to participate in three distinct pathways under the action of specific stimuli. Indeed, the imine bond can be relatively stable under neutral aqueous conditions, while acidic conditions can promote the hydrolysis of the imine bonds with the consequent reformation of the original functionalities through the Schiff base dissociative pathway. Imines are also sensitive to two associative pathways, transimination and imine metathesis, both including exchange reactions taking place in the absence of water.^124^ Transimination refers to the reaction of an imine with a primary amine to form a new imine and a new primary amine. Conversely, the metathesis pathway consists of the reaction between two imines to generate two new imines.

A wide number of polyesters containing imine bonds in their structure have been designed. The combination of Schiff base linkages and ester functions is attractive for the development of biobased self-healable adhesives. As an example, ethyl cellulose was modified by functionalization of the backbone with vanillin methacrylate and lauryl methacrylate.^125^ The resulting polymers were crosslinked by inducing the formation of Schiff base bonds between the aldehyde functions in the pendant vanillin groups and a polyetheramine. Shear strengths around 0.81 MPa and a self-healing efficiency of ∼99% could be reached. Following the same approach, a lignin-based dynamic network crosslinked by inducing a Schiff base reaction between vanillin methacrylate and a diamine was realized.^126^ Besides the self-healing, the resulting polymer also presented UV-shielding and antifungal properties. Although still not investigated in terms of recyclability and/or biodegradability, poly(azomethine esters) have been reported to be promising for demanding aerospace and automotive applications in light of their high thermal resistance, low band gap, and semiconductive properties.^127−129^

#### 5.1.1 Chemical Recycling

Polyester-imine thermosets have been developed, aiming to meet the requirements of circular economy, including the possible obtainment from renewable resources and the facile recyclability at the end of service life. Among the large library of renewable resources, levulinic acid is considered an important biomass-derived molecule due to its functionality,^130^ utility as a solvent, and the possibility to derive it from lignocellulose waste.^131,132^ In the frame of chemically recyclable polyester-imine thermosets, levulinic acid was employed for the synthesis of ELA, a ketone-ester-epoxy precursor, subsequently cured with 2-(4-aminophenyl)-1*H*-benzimidazol-5-amine (BIA) or 4,4′-diaminodiphenylmethane (DDM) to synthesize dynamic covalent networks with *in situ* generated imines and multiple hydrogen bonds.^133^ The resulting ELA-BIA thermosets had high *T*_g_ and modulus up to 165 °C and 2422 MPa, respectively. DDM was selected for comparison to prepare ELA-DDM because it is an amine-based agent often employed for the curing of epoxy resins.^134,135^ The thermosets contained in their structure both imine bonds and noncovalent hydrogen bonds formed between the available amine and hydroxyl groups in the network. Both the imine and hydrogen bonds endowed the thermoset with malleability and rapid self-repairing properties in comparison to values commonly reported in literature.^103,136^ Although the *T*_g_ of ELA-BIA was 20 °C higher, its relaxation time (1838 s) was significantly faster than that of ELA-DDM (4588 s) at 170 °C. The chemical recyclability was proven, and the maximum degradation rate was observed in mixtures with an ethanol:acidic water ratio of 8:2. On the other hand, slow degradation rates were registered for mixtures with higher ethanol content, likely because the amount of water was not enough to induce the hydrolysis of imine bonds. ELA-DDM turned out to be more easily degradable than ELA-BIA; this was ascribed to its lower crosslink and hydrogen bond density, which likely makes the Schiff base bonds more accessible to the water molecules.

**Figure 13:**
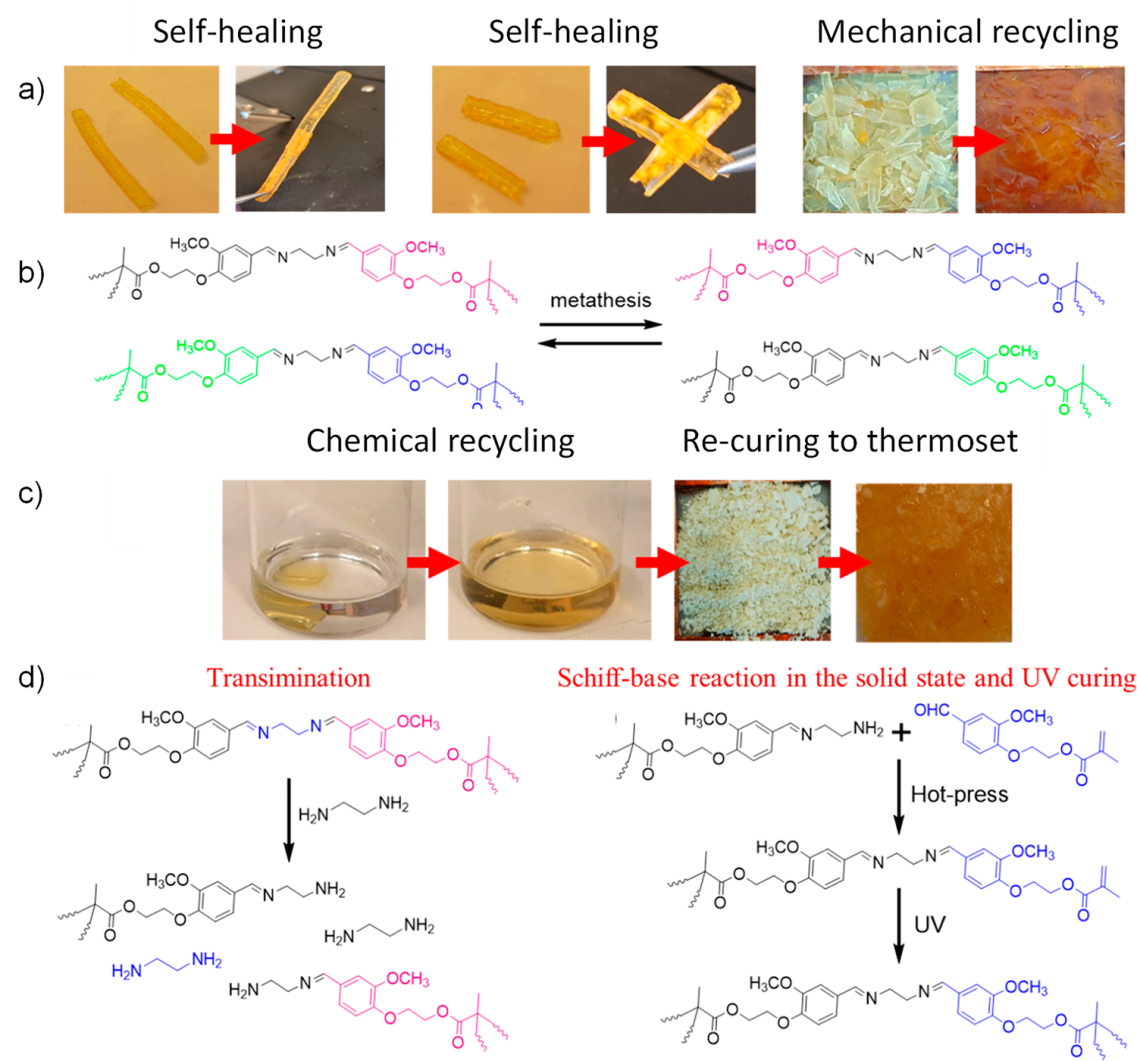
(a) Images of the samples before and after the self-healing and mechanical recycling by hot pressing. (b) Metathesis pathway enabling self-healing and mechanical recycling. (c) For the chemical recycling, the Schiff base thermoset was immersed in ethylene diamine at 60 °C. The recycled oligomeric product was recovered as powder and mixed with virgin methacrylated extended vanillin and photoinitiator before hot pressing and UV curing. (d) Possible transimination pathway to solubilize the cured thermoset in ethylene diamine and possible reactions occurring during hot-pressing and UV curing of the product recovered from chemical recycling and added fresh methacrylated extended vanillin monomer. Adapted with permission from ref (139). Copyright 2022 The Authors CC-BY 4.0. Published 2022 Elsevier Ltd.

In the framework of photopolymerized imine thermosets, vanillin and several other biobased aromatic aldehydes are particularly suitable. The phenolic hydroxy groups can be functionalized by (meth)acrylation, and the imine groups are introduced by the Schiff base reaction between the aldehyde group and di- or trifunctional amines. Recently, several (meth)acrylated vanillin Schiff base resins curable to thermosets by photopolymerization under a UV lamp or by digital light processing 3D printing were prepared.^137−139^ The physical properties of the resulting thermosets increased with higher crosslink density, which was tunable by selection of the amine (e.g., *T*_*g*_ could be varied from −26 to 83 °C and storage modulus at 20–25 °C from 3 to 3300 MPa). In terms of mechanical recyclability, the grinded thermosets turned out to be reprocessable to continuous films by hot-pressing, thanks to the activation of the imine metathesis pathway (Figure 13). The reprocessed thermosets typically exhibited somewhat lower mechanical properties in comparison to the original samples.

Probably due to the hydrophobic nature of the networks, imparted by the presence of the aromatic vanillin moieties, the networks did not dissolve in acidic aqueous solution. Therefore, chemical recycling by introduction of different amines was investigated to exploit the transimination pathway and imine exchange reactions. The first adopted approach with monoamine demonstrated the dissolution of network structures, but it had a limitation in the inability to reform a crosslinked thermoset structure.^137^ The replacement of the hexylamine with ethylene diamine resulted in complete dissolution after 4 h at 60 °C, suggesting the occurrence of transimination leading to the transformation of the thermoset into a noncrosslinked oligomeric product (Figure 13), which was further confirmed by ^1^H NMR and FTIR spectroscopy.^139^ The recovered product could be mixed and hot-pressed with fresh methacrylated vanillin to induce the formation of new Schiff base linkages between the amine-terminated oligomers and methacrylated vanillin. The chemically recycled thermosets showed an elastic modulus comparable to the mechanically recycled films (342 MPa for the chemically recycled thermoset vs 316 MPa for the mechanically recycled thermoset). Interestingly, the introduction of carbon dots (CD) in methacrylated Schiff base resins before the digital light processing 3D printing had a profound impact on the mechanical and chemical recyclability of the resulting thermosets.^140^ While a significant decrease in elastic modulus was observed for the Schiff base thermosets, the presence of CD importantly mitigated this phenomenon, resulting in an almost complete preservation of the original mechanical properties in both mechanically and chemically recycled thermosets. This outcome was attributed to the establishment of secondary interactions in the thermosets, highlighting the synergistic effect of dynamic covalent bonds and supramolecular chemistry in enhancing the recyclability.

Designing polymers for facile closed-loop chemical recyclability represents a step forward in the development of sustainable polymers and thermosets, in particular to reduce the need of virgin resources, whether nonrenewable or renewable, and to avoid waste accumulation. In this frame, an interesting class of polyester-imine thermosets was proposed.^141^ The process included first the synthesis of a polyester prepolymer by ring-opening copolymerization between cyclic anhydride, an epoxide comonomer, and vanillin as a crosslinker. In the second step, the network was crosslinked with a diamine to form covalent adaptive networks. Thanks to the activation of the imine metathesis pathway, the obtained thermosets demonstrated good mechanical recyclability, enabled reaching an almost complete recovery of the mechanical properties by performing a hot-press step at 100 °C for 30 min. Furthermore, from the FTIR analysis, no chemical changes were detected in the chemical structure of the thermosets after the mechanical recycling. Similar to the previously reported works, the dissociation of the imine bonds was triggered under acidic conditions. By inducing a precipitation in methanol, it was possible to recover the prepolymer with a yield of 91%. ^1^H and ^13^C NMR confirmed the retention of a structure similar to the original prepolymer. However, the presence of a small number of pendant amines and the occurrence of dimerization was also confirmed. A further step to definitely close the loop would be a demonstration of reuse of the prepolymer to reform the thermoset by a Schiff base reaction with diamine.

**Figure 14:**
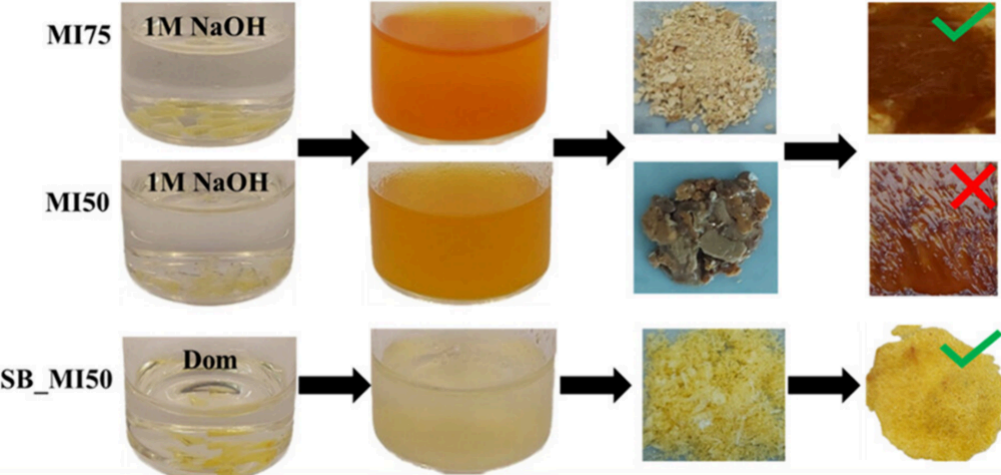
Chemical recycling scheme for polyester (MI75 and MI 50) and polyester-imine (SB_MI50) thermosets including images of the resulting recycled products and recured thermosets. Reproduced with permission from ref (127). Copyright 2023 The Authors CC-BY 4.0. Published 2023 American Chemical Society.

The synergistic effect of ester and imine functions to facilitate the chemical recyclability was recently demonstrated.^142^ A resin was composed of 50 wt % methacrylated isosorbide monomer and 50 wt % methacrylated vanillin Schiff base monomer. The chemical and mechanical recycling of the resulting thermoset, produced by means of digital light processing 3D printing, was compared with printed thermosets obtained from a resin composed of 75 or 50 wt % of methacrylated isosorbide and 25 or 50 wt % of methacrylated vanillin (i.e., two resins contained only ester groups and no imine groups). The 75/25 ester thermoset (MI75) proved to be chemically recyclable but exhibited a drastic decrease in the elastic modulus (2 GPa for the original thermoset vs 0.4 GPa for the recycled one). Conversely, the elastic modulus of the thermoset containing both ester and imine groups (SB_MI50) was preserved after the chemical recycling procedure (1.1 GPa for the original thermoset vs 1.0 GPa for the recycled one). An attempt to recycle the thermosets obtained from 50 wt % methacrylated isosorbide monomer and 50 wt % methacrylated vanillin (MI50) was not successful, only yielding a jelly-like sticky material, supporting the important role of the imine functionalities (Figure 14).

#### 5.1.2 Biodegradation and Hydrolysis

The potential biodegradation of polyester-imines has been minimally investigated so far. One paper, however, documented the biodegradation potential of linear polyester-imines. First, biodegradable PBS or poly(butylene adipate) (PBA) oligomers with two hydroxyl end groups and a diol compound incorporating imine bonds were coupled by reaction with hexamethylene diisocyanate.^143^ After the synthesis, polymer films were obtained by hot-pressing at 130 °C for 5 min. All the prepared films exhibited mechanical properties and *T*_m_ comparable to LDPE and they were stable in contact with moisture in air. However, they disintegrated in water, with degradation times spanning from 1 h to a few days. The degradation rate could be controlled by the concentration of imine bonds, and the films could be reformed by evaporation of the water and a drying step at 80 °C. In addition, biodegradation of the polymer containing PBS oligomers was tested under simulated industrial composting conditions at 58 ± 2 °C. The degree of biodegradation was quantified by following the amount of CO_2_ produced, indicating promising biodegradation degrees of 16% after 10 days and 66% after 38 days. It would have been interesting to continue the biodegradation testing to see if >90% biodegradation can be reached.

Linear polyester-imine polymers, such as oxime and/or imine linked PEG-like polymer, have also been developed.^144^ As a first step, a difunctional PEG-1k benzaldehyde monomer was prepared by exploiting a carbodiimide coupling reaction between dihydroxy PEG-1k and 4-formyl benzoic acid; as a consequence, ester groups were incorporated in the monomer structure. The two terminal aldehydes of the resulting monomer were then reacted with hydroxylamine or a difunctional amine to form oxime and imine groups, respectively. PEG-like polymers comprising of 100% oxime (PEGox), 100% imine (PEGim), or a mixed composition were prepared. The hydrolytic degradation of the polymers was evaluated in a phosphate buffer at pH 7.4. PEGox showed the slowest degradation rate, retaining 75% of its molar mass after 5 days of immersion. Conversely, PEGim dissolved completely after only 4 days. Concerning the polymers with both functional groups, they all showed fast initial weight loss correlating to the fraction of imine bonds in the polymer. As an example, a polymer with 25% imine bonds quickly lost 21% of its initial mass in few hours; then, the weight loss slowly increased to 36% over 5 days of immersion due to the higher hydrolytic stability of the oxime functions. These are interesting results, but further studies should confirm the potential biodegradability of the resulting water-soluble products.

The reported papers mainly exploited the presence of imine functionalities to confer chemical recyclability or degradability to the thermosets. Although ester groups were present, their role in the process was mainly not considered or utilized. A different approach was adopted for a series of linear Schiff base polyesters with different aliphatic/aromatic ratios.^145^ The polymers were synthesized by subjecting vanillin to a nucleophilic substitution reaction, followed by a Schiff base reaction and a two-step bulk polycondensation protocol (transesterification and polycondensation). Several polyester-imines were subjected to a PETase enzyme for 1 and 24 h at 30 °C. A control experiment was performed under the same conditions but in the absence of the enzyme. A comparison of the samples treated with and without enzymes showed a significant increase in the amount of the degradation products in the presence of enzymes. Furthermore, it was shown that both ester and imine bonds were cleaved during the enzyme-catalyzed hydrolysis. The analysis of the degradation products highlighted the role of the chemical structure on the enzymatic degradability. Aliphatic ester bonds turned out to be more easily cleavable than the aromatic counterpart despite the known ability of PETase to catalyze the hydrolysis of aromatic PET. Moreover, the replacement of aromatic diamines with long aliphatic diamines favored the degradation, probably due to an increase of the substrate flexibility and facilitation of the binding of the enzyme to the active site. In addition to enzyme-catalyzed green chemical recycling, these results give positive indications of potential biodegradability of the materials. Authors also investigated the hydrolytic degradation under acidic conditions at room temperature during 24 h. This approach enabled the obtainment of monomeric dialdehydes and diamines. The recovered monomers did not coincide with the original ones, because the ester bonds were not affected by this procedure. However, the obtained molecules are expected to repolymerizable back to the original polymer structures through Schiff base reaction between the aldehyde and amine functionalities, providing a closed loop.

In conclusion, the incorporation of imine bonds in polyesters is a promising route to circular polymers. Polyester-imines can be fully produced from renewable resources as many suitable aldehydes, ketones, and amines are derivable from biomass, and some already commercial examples include vanillin, levulinic acid, and fatty acid derived diamines. Imine bond provides circularity by both mechanical and chemical recycling routes. Furthermore, recent studies indicate that the imine bond could also be biodegradable, although more studies are required to confirm this and the structural requirements for biodegradation of polyester-imines. For some applications, the imine hydrolysis under acidic aqueous conditions can be a limiting factor. However, the stability of polyester-imines in neutral water is typically good and some are also resistant under moderate acidic conditions.

### 5.2 Polyester-Disulfide

Traditionally, sulfide bonds exist in vulcanized rubber. More recently, the disulfide bond (S–S), that can be formed through the oxidation reaction of thiols, has been widely explored as a covalent bond capable of introducing self-healing, reprocessability, recyclability, and even (bio)degradability. Disulfide bonds are able to cleave into their respective thiol counterparts under reductive conditions and reconnect again under oxidative conditions.^146^ Therefore, under controlled pH conditions (reductive or oxidative), the formation and cleavage of the disulfide bond can be manually controlled. Interestingly, some natural environments with a low oxidation reduction potential (i.e., seawater and sediment, river water, and sediment) can also facilitate the cleavage of the disulfide bond into their initial form (i.e., thiols). The reduced compounds can be further metabolized to inorganic compounds, such as CO_2_ and SO_3_, by microorganisms. The incorporation of disulfide bonds into a polymer backbone could also facilitate controlled chemical recycling under specific pH conditions, as well as impart the polymer with (bio)degradation in natural environments.^282^

#### 5.2.1 Chemical Recycling

**Figure 15:**
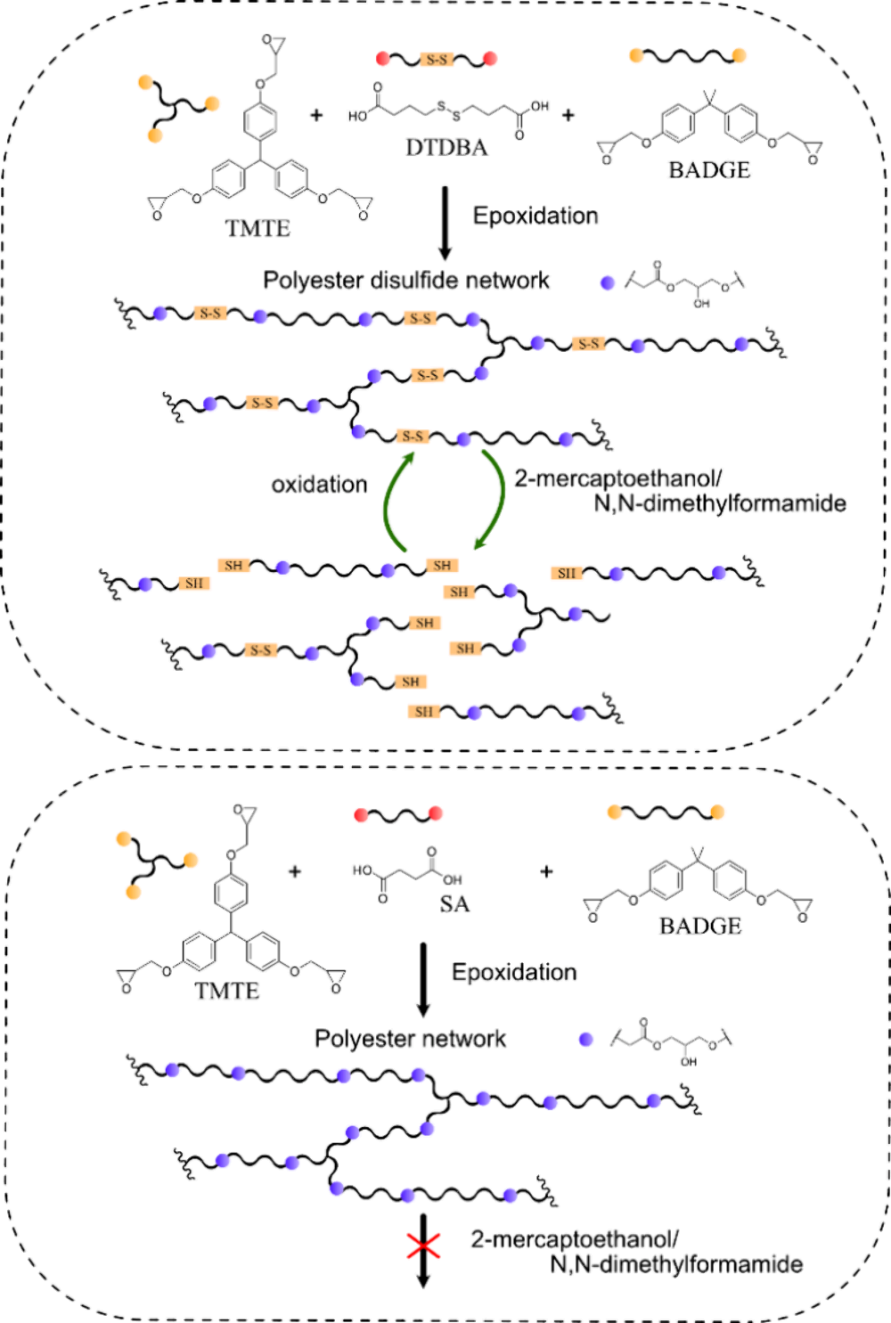
Synthesis of epoxy-thermosets with ester and disulfide or only ester linkages. The presence of disulfide linkages enabled chemical recycling under mild conditions in the presence of reducing agent 2-mercaptoethanol. The thermoset with only ester bonds remained stable under the same conditions.^147^

The potential of disulfide bond in imparting polyesters with (improved) chemical recyclability has so far been hardly explored. Research with regard to chemical recycling and degradation of disulfide containing polymers has mainly focused on the synthesis of recyclable thermosets. In this regard, a series of novel recyclable thermosets were designed to have ester and disulfide linkages, using simple condensation and epoxy chemistry.^147^ The epoxy ester thermosets were synthesized by reacting 4,4′-dithiodibutyric acid (DTDBA) with two epoxy monomers; difunctional bisphenol A diglycidyl ether (BADGE) and trifunctional triphenylolmethane triglycidyl ether (TMTE), to form thermosets with ester or ester and disulfide linkages, varying in crosslink density (Figure 15).

The resulting thermosets exhibited good mechanical strength (reaching 29.7 MPa with maximum crosslink density) and low to moderate flexibility (14–92%). Moreover, all thermosets were thermally stable up to 260 °C. At the same time, the materials showed good mechanical recyclability at temperatures 120–200 °C without significant loss in mechanical properties even after two reprocessing cycles. This thermal reprocessability was attributed to the dynamic behavior of the disulfide bonds. Chemical recycling of the thermosets was evaluated by immersing the thermosets in pure dimethylformamide (DMF) and a mixture of DMF with the reducing agent 2-mercaptoethanol, respectively. The thermosets retained their original shape after 36 h in pure DMF. However, in the presence of a reducing agent, the thermosets had been completely dissolved after 36 h. For comparison, a similar ester-based thermoset without disulfide bonds, synthesized by replacing DTDBA by succinic acid (SA), did not show any decomposition in the presence of the reducing agent. The chemical recycling was therefore facilitated through the reversible cleavage of the disulfide bond under reductive conditions.

A similar approach was taken to design a series of polyester-disulfide based thermosets from different multifunctional epoxidized vegetable oils (EVO) and 2,2′-dithiodibenzoic acid (DTBA).^148^ The thermosets were, again, synthesized via facile condensation and epoxy chemistry, where the epoxy groups in the vegetable oils reacted with the acid groups in 2,2′-dithiodibenzoic acid to form ester linkages. The formed thermosets, containing both disulfide and ester bonds, exhibited good thermal stability (260–290 °C) and varying mechanical strength (0.39–11.5 MPa) depending on the vegetable oil used. Full chemical recycling was obtained within 24 h at 50 °C through the reduction of the disulfide bonds in a solution of DMF with 5 wt % of the reducing agent dithiothreitol. A metathesis reaction between the disulfide groups in the thermoset and the thiol groups in the reducing agent resulted in complete cleavage of the disulfide bonds in the thermosets. No control experiments were performed, but similar results were obtained for other epoxy resins with exchangeable disulfide crosslinks and ester bonds.^149^

Partially biobased disulfide-containing vitrimers were synthesized using isosorbide-derived epoxy and aromatic diamines containing disulfide linkages, aiming to replace the fossil fuel derived and toxic BPA by biobased isosorbide, derived from carbohydrates.^150^ The obtained vitrimers exhibited reprocessability and self-healing at moderate to high temperatures (130–170 °C) due to the metathesis reactions of the disulfide bond above the *T*_g_ of the thermoset (>35 °C). Moreover, the isosorbide-disulfide based thermosets showed comparable mechanical properties to similar epoxy networks cured by traditional nondynamic curing agents and crosslinkers. The chemical recyclability/degradability of the thermosets were evaluated under reductive alkaline conditions. After being immersed in an aqueous solution of 5 wt % NaOH, initial degradation was observed after 1 h and the thermosets with higher content of isosorbide fully degraded after only 3 h. Thermosets with high isosorbide content are also expected to have lower negative effect on the environment compared to, e.g., BPA based thermosets.

These studies show that chemical recyclability of polyester can be improved and controlled by the incorporation of disulfide linkages by utilizing the dynamic behavior (i.e., cleavage) of the disulfide bond under reductive conditions. In some cases, the disulfide bond could cleave even without the presence of a reducing agent. This was, for example, observed for thermosets with a high sulfur content synthesized via two-step inverse vulcanization processes.^151^ For a first step, a trifunctional aliphatic monomer (span 80) containing an ester group, a carbon–carbon double bond, and three hydroxyl groups, was used to react with sulfur to form a linear prepolymer. During this step, the sulfur reacted with the carbon–carbon double bonds in the aliphatic monomers to form a linear saturated polymer chain with disulfide bonds, ester bonds, and free hydroxyl groups. During the second step BADGE, as a difunctional aromatic crosslinker with epoxy end groups, was added. The low reactivity between the hydroxyl groups on span 80 based prepolymer and the epoxy groups on BADGE resulted in a crosslinked network containing disulfide, ester, ether and hydroxyl groups.

The resulting polymer network exhibited combination of good mechanical strength (13 MPa), flexibility (elongation at break of 110%), and toughness (1.3 kJ/m^3^). Interestingly, while the crosslinked polymers showed good solvent tolerance in most organic solvents, they could have chemical recyclability in polar solvents such as DMF, dimethylacetamide (DMAc), and *N*-methyl-2-pyrrolidone (NMP). A careful evaluation of the soluble and partially soluble fractions showed that the molar mass of these fractions was significantly lower than before dissolution, which is expected to be caused by disulfide cleavage induced by the solvent. While no control experiments were carried out on polymers without disulfide linkages, other studies recently confirmed that polar solvents, such as pyridine, can chemically break disulfide bonds, which allows for the dissolution and chemical recycling of disulfide-based networks.^152^

Instead of using the reductive-labile nature of the disulfide bond, a different chemical recycling approach could utilize the metathesis exchange reaction of the disulfide bond. Some epoxy resins containing ester and reversible disulfide bonds were reported to degrade into oligomers by the metathesis exchange reaction of the disulfide bond in the presence of thiols.^153,154^ For example, self-healing malleable epoxy resins containing ester and disulfide bonds were synthesized from bisphenol A, 1,4,5-oxadithiepane-2,7-dione, and methylhexahydrophthalic anhydride.^155^ The resulting thermoset could be fully recycled by immersing it in a solution of di-*tert*-butyl disulfide in DMF at 140 °C for 0.5 h with 4-dimethylaminopyridine and triphenylphosphine as catalysts. The incorporation of disulfide bonds, thereby presents an interesting option for imparting polyesters with improved and controlled chemical recyclability. However, the research on the resulting low molar mass products has been limited, and it is still to be shown whether these products can also be utilized for synthesis of new thermosets. The studies highlighted the facile chemical recyclability of thermosets containing disulfide bonds in their structures. Given this high reactivity, it is imperative to strike a balance between chemical recyclability and durability while also identifying the most promising applications for these thermosets. This assessment should include the optimization of relative concentrations of disulfide and ester bonds on the chemical recyclability of the resulting materials.

#### 5.2.2 Biodegradation and Hydrolysis

The ability of the disulfide bond to readily cleave into thiols under reductive conditions can potentially facilitate biodegradation under environmental or composting conditions.^156−158^ The redox responsiveness of the disulfide bond was already utilized for fabrication of materials for controllable degradation in biomedical applications. For example, citrate-based polyester elastomer with regulatable and controllable degradation rates were synthesized by incorporating disulfide bonds in the polymer network.^159^ Degradation was evaluated in phosphate buffered saline alone or with either dithiotheitrol (DTT) or intercellular reducing agent glutathione (GSH). The networks lacking disulfide bonds showed no noticeable degradation, and no significant weight loss was observed. However, for networks containing disulfide bonds, noticeable degradation was observed and there was significant difference between degradation in phosphate buffer with or without DTT. The faster weight loss in the presence of DTT was attributed to the cleavage and exchange reactions between disulfide bonds of the network and thiol groups of DTT. Moreover, the degradation rate gradually increased with increasing disulfide content. These results clearly demonstrate that the accelerated degradation rate was related to the disulfide bonds in the polymer networks. In the presence of the reducing agent, GSH, the degradation rate was accelerated up to ∼30 times depending on disulfide content. Similarly, a disulfide-containing nanogel demonstrated fast degradation kinetics in the presence of 11 mM GSH, degrading into small, low molar mass dithiol-based molecules.^160^*In vitro* studies showed low toxicity of the nanogels and their degradation products.

**Figure 16:**
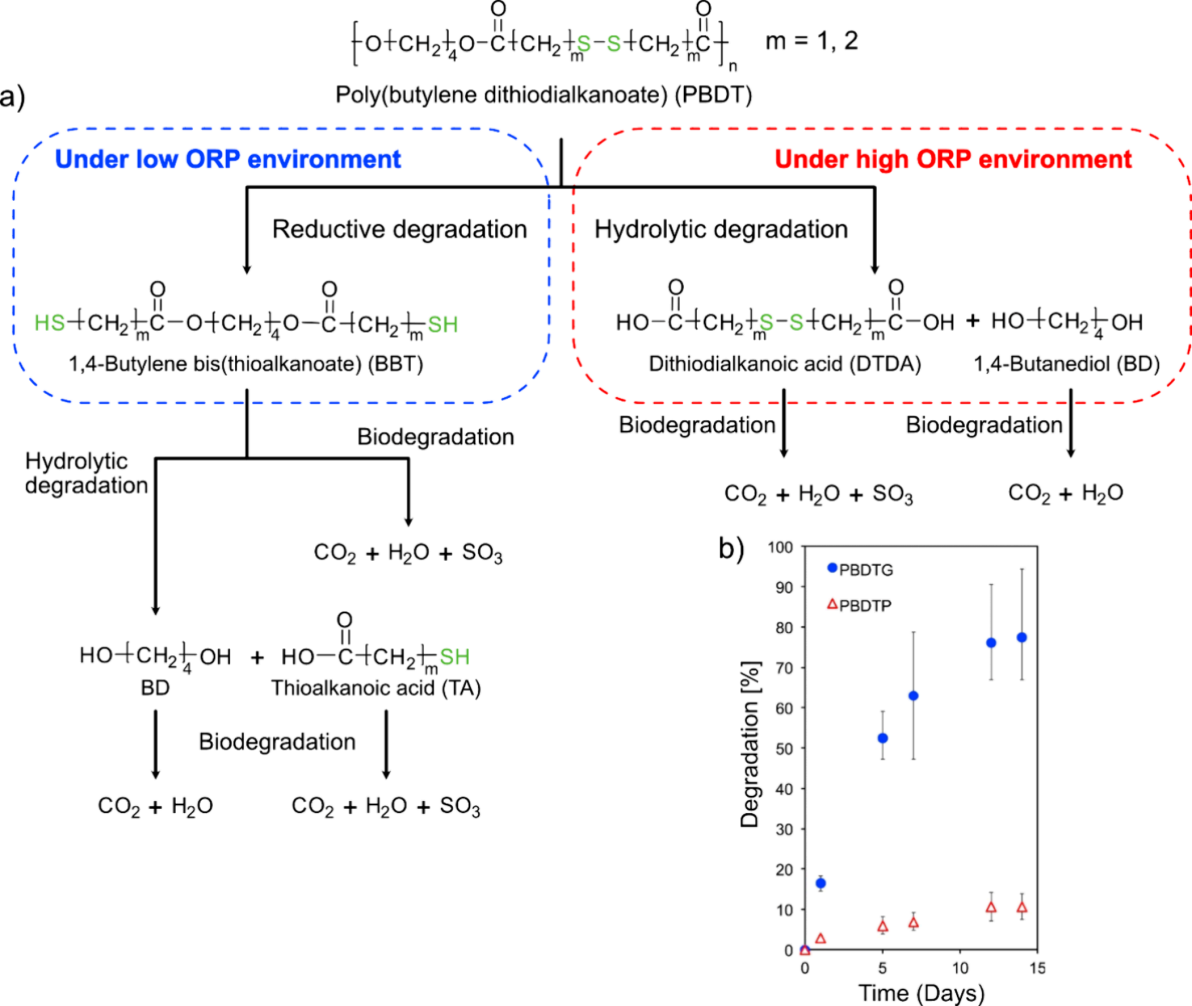
Proposed biodegradation route for disulfide containing poly(butylene dithioalkanoate) polyesters under low and high ORP environments (a) and the degradation of rate for the two disulfide containing polyesters (PBDTG and PBDTP) under abiotic reductive environment measured through the release rate of thiol-monomers (b). Adapted with permission from ref (146). Copyright 2017 Elsevier.

GSH is a strongly reducing, intercellular antioxidant with a low oxidation–reduction potential (ORP) of −240 to 289 mV. Its presence inside the cell leads to appropriate reductive conditions for triggering the cleavage of the disulfide bonds.^161,162^ Such reductive conditions can also be found in some natural environments, where the ORP can be sufficiently low to cleave disulfide bonds. For example, this concept was applied for the development of poly(butylene dithiodialkanoate) (PBDT) polymers with ester and disulfide bonds in the main chain.^146^ The disulfide-containing polyesters (poly(butylene dithiodiglycolate) (PBDTG) and poly(butylene dithiodipropionate) (PBDTP) were shown to be susceptible to abiotic reductive cleavage of the disulfide bonds in lower ORP conditions that were similar to that of seawater sediment (Figure 16). PBDTG released 76% of the thiol containing monomer within 14 days, while the more hydrophobic PBDTP only released 11%. Furthermore, the further biodegradation potential of the thiol-monomers released by reductive cleavage was shown by biological oxygen demand (BOD) test. Reductive cleavage of the disulfide bond was not observed in environments with high ORP, indicating their stability under normal conditions.

A similar approach has been applied through incorporation of disulfide bonds in PBS.^163,164^ PBS is a promising biobased polymer with good mechanical properties and biodegradability. However, the biodegradability within shorter time frame is generally limited to compost and soil. The incorporation of disulfide linkages in the main chain of PBS enhanced the degradation rate in simulated seawater.^163^ The series of disulfide containing PBS polymers, named as PBSDT, were synthesized via the polycondensation of 1,4-butanediol, succinic acid, and different amounts of dithioglycolic acid, with titanium tetraisopropoxide as a catalyst. By replacing certain amounts of succinic acid units by dithioglycolic acid, the disulfide content in the polymer backbone could be controlled and varied. The resulting semicrystalline polymers showed *T*_d5%_ and *T*_m_ between 254 and 320 °C and 68–117 °C, respectively. Both temperatures decreased with increasing content of disulfide linkages in the main chain.

**Figure 17:**
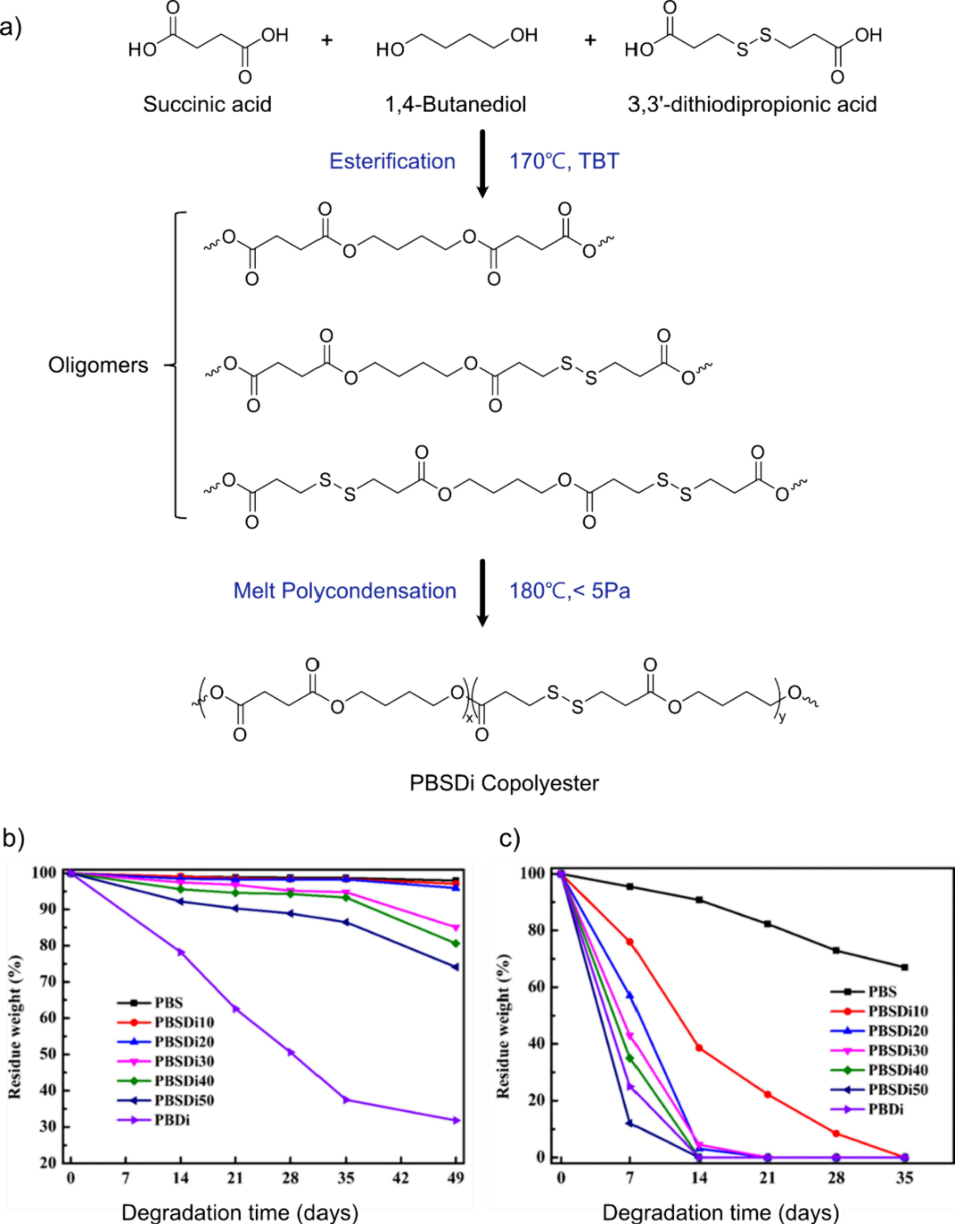
(a) Simplified scheme for the synthesis procedure of disulfide modified PBS denoted as PBSDi copolymers. (b) Abiotic hydrolysis and (c) CALB enzyme-catalyzed hydrolysis of PBS and PBDi homopolymers and PBSDi copolymers with different disulfide contents. Adapted with permission from ref (164). Copyright 2023 American Chemical Society.

Reductive degradation of the polymers was assessed by simulating low ORP conditions to facilitate the disulfide bond cleavage, using the reducing agent DTT to make a buffer solution with an ORP of −94 mV. Powdered PBSDT were placed in the buffer solution for 9 days at room temperature. After the reduction, only small amounts of residual polymer could be recovered. The *M*_n_ of the PBSDT significantly decreased under low ORP conditions, suggesting the formation of low molecular species by reductive disulfide bond cleavage. The ORP value of −94 mV is significantly higher compared to average values found in seawater (−150 and −200 mV).^165,166^ It is thus possible that degradation would also take place in seawater with the lower and more favorable ORP values. When the PBSDT was treated under high ORP conditions 214 mV, no molar mass decrease was observed. This suggests that no ester hydrolysis or disulfide bond cleavage took place, again indicating that the polymers are stable under normal conditions. In another study PBS was modified by replacing different percentage of the succinic acid units by 3,3′-dithiodipropionic acid.^164^ It was shown that both the abiotic hydrolytic rate in phosphate buffer, but even more clearly the enzyme-catalyzed hydrolysis rate, were accelerated and increased as a function of disulfide-bond content (Figure 17). The incorporation of disulfide bonds into PBS could thus potentially extend the biodegradability in soil and compost environments to even marine environments. Moreover, the low molar mass compounds, produced by degradation, are expected to be further metabolized by microorganisms into inorganic compounds.

These results show the potential of disulfide bonds to facilitate the degradation of polyesters under environmental conditions. This is fully attributed to the reversibility of the disulfide bond, able to cleave under low ORP conditions, which can be encountered in seawater and sediment with typical ORP values between −150 and −200 mV.^165,166^ At the same time, the disulfide bonds seem to be stable under high ORP conditions. Disulfide-containing polyesters are thus potentially promising sustainable plastics that are stable and durable when in use but potentially degradable into low molar mass compounds that can be further metabolized upon unwanted disposal to the environment. The relatively fast degradation of the disulfide bond in the presence of a reducing agent or small-molecule thiols/disulfides facilitates recyclability and (bio)degradability under facile conditions. However, more research on the chemical recycling and degradation products is needed. In particular, the possibility to use electrochemistry to facilitate recovery of the chemical reducing agents offers an interesting path forward.

#### 5.2.3 Mechanical Recycling

Compared to the scientific infancy in the utilization of disulfide bonds to enhance chemical recycling and (bio)degradation, the application of these bonds in mechanically recyclable thermosets is more mature. Self-healing properties and mechanical recyclability are introduced by the capability of disulfide bonds to undergo two associative dynamic bond-exchange mechanisms: disulfide metathesis and exchange reactions. Similar to the imine, disulfide metathesis refers to the reaction between two disulfide bonds to form two new disulfide bonds. Disulfide exchange reactions take place between a thiol and a disulfide bond to form a new thiol and new disulfide bonds. Such bond rearrangements can occur under different conditions such as UV-exposure,^167,168^ moderate to elevated temperatures^169−171^ even without the aid of a catalyst,^149,172^ and under mechanical stress,^173^ making them suitable candidates for inducing recyclability under different facile conditions. The incorporation of disulfide linkages in polymer materials such as epoxy resins^149,174^ and polyurethanes^175,176^ has been widely reported. Conversely, polyesters containing disulfide linkages have been explored less. This despite the fact that in comparison to transesterification reactions, the disulfide metathesis and exchange reactions can occur at lower temperatures and without the aid of a catalyst.^177^ The presence of disulfide based crosslinkers can impart polyester based networks with excellent self-healing^178,179^ and thermal reprocessability,^180^ providing promising opportunities for development of circular and sustainable thermosets.

A thermally recyclable polyester-based epoxy resin containing disulfide crosslinks was obtained by conventional epoxy chemistry between and epoxy (diglycidyl ether from bisphenol A) and a disulfide containing acid (4,4′-dithiodibutyric acid).^181^ The resulting dual-dynamic network exhibited fast thermal relaxation rates already at mild temperatures above 65 °C, enabling thermal reprocessability similar to that of thermoplastics. Interestingly, when the obtained relaxation times of the dual dynamic network were compared to networks containing only disulfide or only ester bonds, it was observed that the relaxation times of the dual-dynamic network were ∼28 and ∼122 times faster, respectively. Moreover, the dual dynamic network showed heating-induced malleability above 65 °C, while significantly higher temperatures were required for the single-disulfide network (105 °C) and single-ester network (150 °C). This indicates that the exchange reactions of the dual-dynamic networks are triggered much faster as compared to the single dynamic networks and the synergy between disulfide metathesis and carboxylate transesterification accelerates the rate of exchange reactions. As a result, the dual dynamic network could be reprocessed by hot press (100 °C, 1 h) for four cycles without significant loss of mechanical properties. In another study, fully biobased network containing ester, hydroxyl, and disulfide bonds were synthesized through the reaction of epoxidized starch amylopectin with diallyl disulfide and pentaerythritol tetrakis(3-mercaptopropionate).^170^ The presence of the last two components facilitated rapid disulfide exchange reaction at elevated temperatures (150–230 °C) inducing thermal reprocessability by hot-pressing at 7 MPa and 150 °C. Interestingly, after five reprocessing cycles, the mechanical and thermal properties improved. This self-strengthening effect was ascribed to homogenization of the disulfide groups and starch epoxy regions during the reprocessing, resulting in higher crosslink density.

A dynamic elastomer was realized through the facile polycondensation of biomass derived acids (i.e., succinic acid, adipic acid, and sebacic acid) and the diol-1,4-butanediol.^182^ Small amounts of 3,3′-dithiodipropionic acid and glycerol were added to enable dynamic exchange reactions and to provide crosslinking sites for the network formation and elastomeric behavior. The resulting polyester-based elastomer exhibited good mechanical properties and high flexibility (up to 1700%). Stress-relaxation, indicating disulfide exchange reactions, was observed at temperatures ranging from 120 to 180 °C. The reprocessability of the materials were assessed by cutting the materials into smaller pieces, followed by hot-pressing for 5 min at 180 °C under 10 MPa. No significant loss in mechanical properties was observed after 4 cycles of reprocessing. Another sulfide group containing polyester elastomer was synthesized from biobased diols and diacids.^183^ The double bond in itaconic acid and *a priori* synthesized inverse vulcanized sulfur-polymer were utilized for crosslinking. The resulting crosslinked biobased polyesters showed superior mechanical properties and the ability to be malleable and recyclable. Stress relaxation was observed at >120 °C for the thermoset containing S–S bonds, while this was not observed for the reference thermoset with no S–S bonds. Recyclability by hot-pressing was demonstrated.

**Figure 18:**
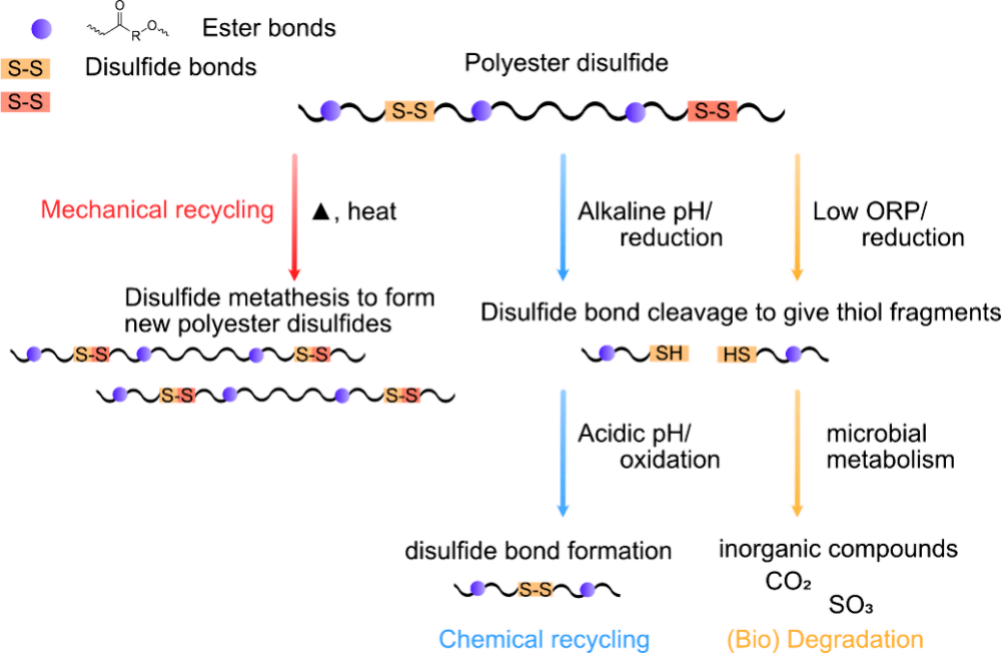
Summary of the disulfide chemistry and how it can enhance mechanical recycling, chemical recycling, and (bio)degradation.

Disulfide containing polyesters can thus be obtained through simple and facile epoxy or polycondensation chemistries. Moreover, there is a vast number of possible monomers from fossil and biobased resources that can be utilized for the design of disulfide containing polyesters. Disulfide exchange reactions are activated at moderate to elevated temperatures, giving the material inherent circularity promoting properties such as self-healing, thermal reprocessability, chemical recyclability, and potentially even higher susceptibility to biodegradation (Figure 18). The combination of disulfide and ester groups within the same network is a promising enabler of mechanical recyclability. However, additional research is warranted to explore the impact of this combination in both aliphatic and aromatic networks to establish both the mechanical recyclability, influence on material properties, and long-term performance of the materials. So far, most studies utilized the disulfide bond for development of thermally reprocessable thermoset, while the potential as enabler for closed-loop chemical recycling and biodegradation should be further investigated. A potential disadvantage with this approach is the unattractive odor of some disulfide compounds.

### 5.3 Polyester-Acetal

Acetal is a functional group with a general structure of R_2_C(OR′)_2_, where two distinct oxygen atoms are single bonded to a central carbon atom. A well-known example of a polyacetal is the commercially available polyoxymethylene (POM), an engineering thermoplastic with high crystallinity and great mechanical properties, as well as chemical stability against many common organic solvents.^184^ The acetal functionality also imparts POM with chemical recycling capabilities, which stem from the ability of acetals to undergo hydrolytic degradation under mildly acidic conditions, with pH < 7.4.^185,186^ Even other acetal-based polymers can potentially be hydrolyzed into alcohol and aldehyde/ketone-functional monomers under acidic conditions, making them promising candidates for the design of circular polymers that can undergo biodegradation and chemical recycling to monomers.^187,188^ Due to the ubiquitous presence of acetal linkages in biodegradable natural polysaccharides, such as cellulose and starch, the introduction of acetal linkages could also facilitate the biodegradation of aliphatic polyesters.

#### 5.3.1 Chemical and Mechanical Recycling

The reversible dynamic nature of the acetal functionality has been widely applied for fabrication of circular thermosetting materials.^189,190^ The recyclability is attributed to the acid-catalyzed dynamic behavior of acetal-bond, enabling exchange reactions such as acetal metathesis and transacetalization. Acetal metathesis is the exchange reaction between two separate acetal structures, while transacetalization is the reaction of an acetal with a hydroxyl group to form a new acetal group. Moreover, the depolymerization of polyacetals in the presence of a strong catalyst can result in the recovery of near quantitative yields of starting monomers.^187^ However, incorporation of acetal groups in polyesters to induce recyclability is still a largely unexplored opportunity.

A polyester-acetal designed for recyclability was synthesized from a biobased building block containing bifuran and glycerol acetal structures (BFG) and end-capped with hydroxyl groups.^191^ This monomer was further polymerized with succinic acid to yield the polyester-acetal (PBFGS), containing bifuran, ester and acetal groups in the main-chain. The thermal properties of the obtained polyester-acetals were evaluated and compared to the polyester poly(bifurfurylene succinate) (PBFS), in which the acetal structure was replaced by a −CH_2_– group. *T*_d5%_ increased from 214 °C for PBFS to 331 °C for PBRGS, and *T*_g_ from 43 to 61 °C, respectively. The presence of the acetal groups in the polymer backbone thus enhanced the thermal properties and stability.

Recyclability was evaluated by facilitating the acetal-exchange reaction in the presence of alcohol (i.e., glycerol). Heating the polyester-acetal in glycerol at 150 °C for 3 h promoted acetal-exchange reactions in the polymer backbone, leading to the cleavage of the chain. After extraction of the residues, BFG, the PBFGS monomer, could be quantitatively (99%) recovered. In addition, PBFGS was also recyclable via acid-catalyzed reaction by treating with trifluoroacetic acid, which resulted in the recovery of bifurfural (BFF), a well-known monomer that can be converted to BFG by acetalization with glycerol. No comparison between the recyclability of the polyester-acetal and polyester was performed. Another study demonstrated closed-loop recyclability of polyester-acetals produced from aromatic aldehydes containing different substitutions with halogen side groups and cyclic anhydrides.^192^ In these polymers two ester bonds were connected by a tertiary carbon atom which results in an integrated acetal-ester functionality. Without the presence of solvents or catalysts, the materials were sublimated under vacuum at 180 °C for 8 h to yield a mixture of the corresponding aromatic aldehydes and cyclic anhydride monomers. This nonpurified mixture could be repolymerized to obtain a polymer with the same structure and similar molar mass to the original nonrecycled polymer.

**Figure 19:**
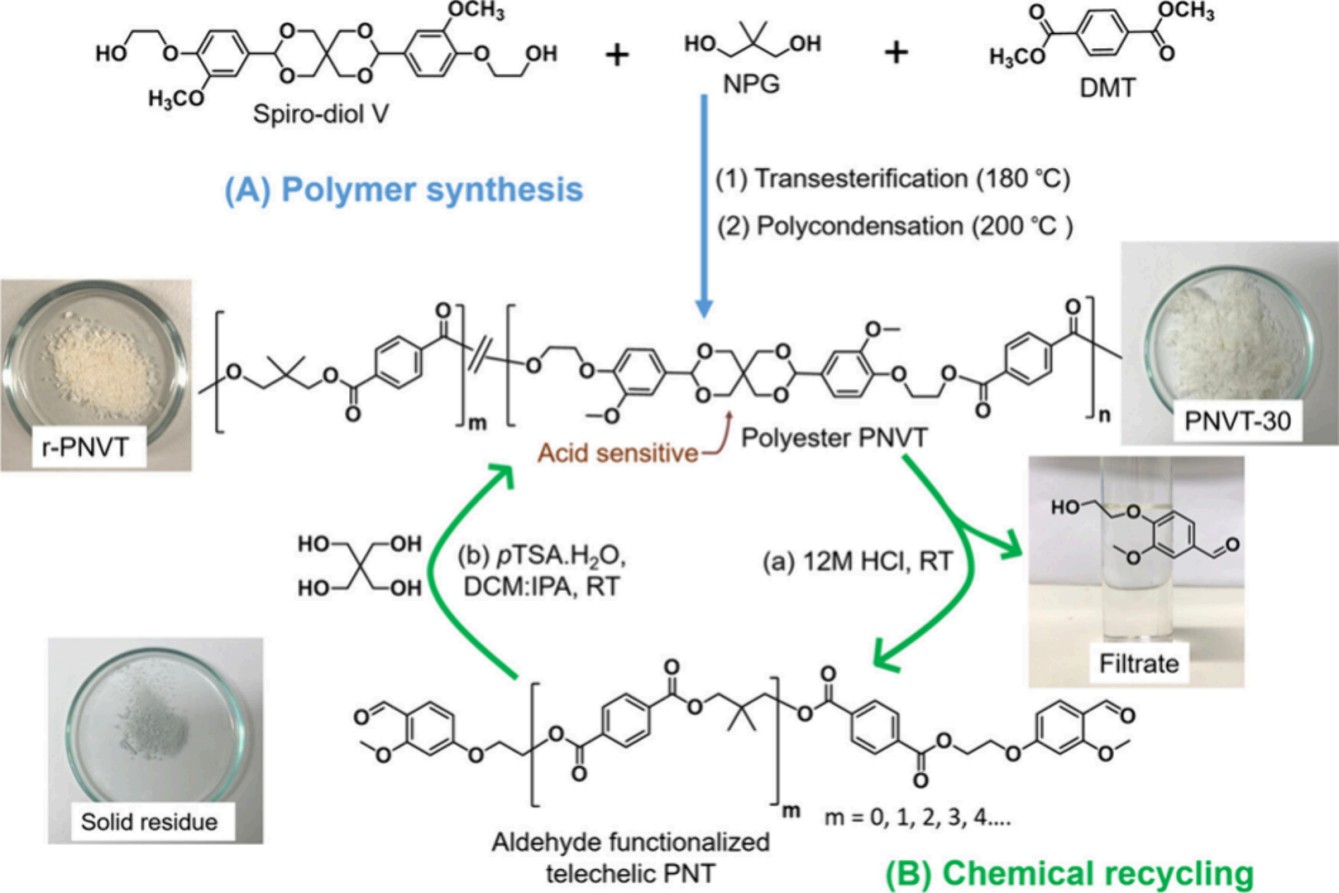
(A) Synthesis of copolyesters with spirocyclic acetal groups. (B) Closed loop chemical recycling of the synthesized polymers. Reproduced from ref (193). Copyright 2023 The Authors CC-BY 4.0. Published 2023 American Chemical Society.

A different approach to acid-catalyzed chemical recycling was taken by designing biobased polyesters with spirocyclic acetal units.^193^ The polyester-acetals were obtained by transesterification and polycondensation reactions of biobased diols (i.e., vanillin based spirocyclic acetal diol and neopentyl glycol) and dimethyl terephthalate at temperatures ranging from 180 to 200 °C. The obtained polyester-acetals exhibited good thermal stability up to 300 °C and relatively high *T*_g_ values between 70 and 105 °C depending on the content of spirocyclic acetal moieties. Chemical recyclability was demonstrated by selective acid-catalyzed hydrolysis of the acetal groups in the polymer backbone, yielding structurally defined telechelic oligoesters with terminal aldehyde groups. These aldehyde end-capped oligoesters could be conveniently converted back to the initial polyester-acetal structures by reaction with pentaerythritol (Figure 19). Apart from the chemical recycling, the dynamic behavior of the acetal groups was also utilized for design of dual-dynamic thermally recyclable polyester-based CANs.^194^ The networks were designed by using styrene as the main monomer and maleic anhydride and acetal diol as crosslinkers. The dual dynamic CAN exhibited relatively high *T*_g_ (<97 °C) and good thermal stability with *T*_d5%_ > 225 °C. Moreover, the presence of the styrene monomer and crosslinkers endowed the networks with high tensile modulus (1.5–1.8 GPa) and tensile strength (20–40 MPa). The dual-dynamic CAN exhibited faster stress relaxation capabilities (47 s at 200 °C) compared to the single-dynamic ester CAN (710 s at 200 °C), indicating synergy between ester and acetal groups to accelerate exchange reactions and dynamic behavior. The dual-dynamic CAN showed excellent reprocessability by remolding at mild to high temperatures.

Thus, the acid-labile nature of the acetal group can impart polyester-acetals with chemical recyclability under milder conditions in comparison to corresponding polyesters. More efforts should be devoted to reduce the number of steps required for the chemical recycling procedure and to avoid the use of organic solvents where possible. At the same time the exchange reactions of the acetal functionality triggered at elevated temperatures can improve the thermal reprocessability. The incorporation of acetal groups can also have positive influence on thermal properties, such as thermal stability and *T*_g_. Moreover, acetals can be produced from various biobased polyols (e.g., pentaerythritol and glycerol)^195,196^ with biobased ketones and aldehydes (e.g., levulinic acid, vanillin, furfural),^195,197,198^ or lignin,^199^ making them good candidates for the synthesis of circular biobased polymers.

#### 5.3.2 Biodegradation and Hydrolysis

The susceptibility of polyester-acetals to hydrolysis and biodegradation was demonstrated by the introduction of acetal units in the polymer backbone of a well-known biobased polymer, PBS.^200^ Using a rigid spirocyclic diacetal monomer, a series of poly(butylene succinate-*co-s*pirocyclic succinate) (PBSS) was synthesized via facile melt polycondensations with spiroacetal content ranging from 0 to 80%. Thermal analysis showed that with increasing content of acetal units, the *T*_g_ and thermal stability increased. The *T*_g_ values increased from −34 °C for PBS to 49 °C for PBSS_80_ (80 mol % acetal units). The *T*_d5%_ ranged from 340 to 359 °C for the different PBSS, and the values were appreciably higher compared to PBS (*T*_d5%_ = 320 °C). In addition to improved thermal properties the incorporation of acetal groups also improved the mechanical properties.

The degradability of the polyester acetals in acidic phosphate buffer with and without lipase enzymes (i.e., lipase from porcine pancreas and *Candida antarctica* lipase B, CALB) was monitored at 37 °C for 4 weeks. The hydrolysis rate of the polyester acetals with lower acetal content (<50%) was relatively low, with a detectable weight loss of 1–2.5% after 4 weeks. The addition of lipase enzymes significantly accelerated the hydrolysis rate of low acetal content PBBS, and after 4 weeks the weight loss ranged from 50% and 70% for PBBS_10_ (10% acetal) and PBBS_20_ (20% acetal), respectively. For the materials with higher acetal content (>50% acetal), the hydrolysis was completely inhibited even in the presence of lipase enzymes. Higher concentration of the rigid spirodiacetal moieties likely increased the steric hindrance as well as the crystallinity of the material, thus limiting the enzyme-catalyzed hydrolysis. Degradation was only observed in strong acidic media (1 M HCl), in which the acid-labile acetal groups were cleaved. Similarly, poly(butylene terephthalate*-co-*spirocyclic terephthalate) only degraded in strong acidic environment.^201^ Different exocyclic hemiacetal esters, synthesized from 7-methoxyoxepan-2-one (MOPO), hydrolyzed within minutes in acidic environment (1 M HCl).^202^ The hydrolysis product were low molar mass compounds with alcohol, aldehyde, and carboxylic acid end-groups. In a basic environment (1 M NaOH), complete degradation was not achieved and complex degradation products with higher molar mass were obtained.

**Figure 20:**
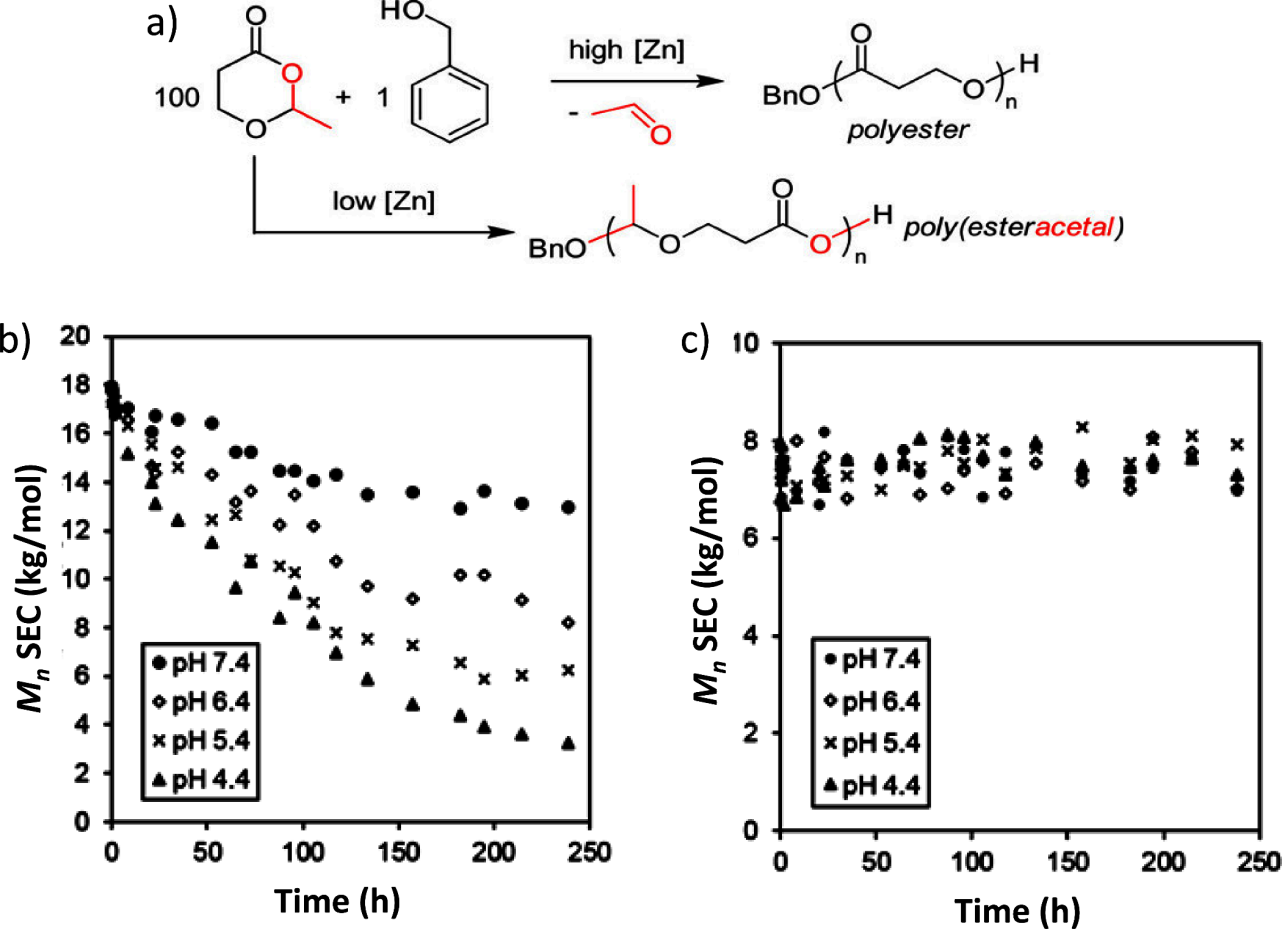
Comparison of the hydrolytic degradation rate of (a) a polyester-acetal (PMDO) and (b) a polyester (PHPA) in aqueous solutions at room temperature and pH range 4.4–7.4. (c) A simplifies scheme over the synthesis and chemical structures of the polyester and polyesteracetal. Adapted with permission from ref (203). Copyright 2014 American Chemical Society.

Another study showed that the adjacent placement of an ester group to the acetal in the polymer chain generates an activated acetal, thus enabling faster hydrolysis and degradation even under relatively mild acidic conditions (pH 7.4–4.4).^203^ During this study, an acid-sensitive degradable polyester acetal was synthesized using a single cyclic ester acetal 2-methyl-1,3-dioxane-4-one (MDO) as a monomer. The single monomer was polymerized in bulk using diethyl zinc as catalyst and benzyl alcohol as the alcohol. By varying the catalyst concentration, the corresponding polyester-acetal (PMDO) with adjacent ester-acetal groups and the aliphatic polyester poly(3-hydroxypropionic acid) (PHPA) were obtained via different polymerization mechanisms (Figure 20).

To assess the susceptibility to hydrolytic degradation, both polymers were subjected to neutral or slightly acidic environments (pH 7.4, 6.4, 5.4, and 4.4) at room temperature for 10 days. During the degradation test samples were taken periodically and the molar mass changes were followed by SEC. As anticipated, the polyester PHPA was stable at the tested pH range and did not degrade appreciably. In contrast, the *M*_n_ of the polyester-acetal, PMDO, decreased significantly with decreasing pH. At the most acidic pH (4.4) the *M*_n_ decreased from 18 kg/mol to only 3 kg/mol over 10 days. In neutral pH of 7.4, a much smaller decrease from 18 to 14 kg/mol was observed. Considering the short time scale of the experiment, the aliphatic polyester-acetal showed susceptibility to degradation even at neutral pH, which indicates promising potential for degradation in a natural environment. These results illustrate how the low hydrolytic stability of the acetal group facilitates the degradation in neutral to acidic environments. Although the degradation products and recyclability efficiency of the above-mentioned polyester-acetals were not further studied, the susceptibility of the acetal group to hydrolysis could likely also be used for facile chemical recycling of polyester-acetals.

It was also demonstrated that enzymes can further catalyze hydrolysis of polyester-acetals, enabling their degradation in natural environments with neutral pH values.^204^ A polyester-acetal was synthesized from a bicyclic carbohydrate-based monomer, dimethyl 2,3;4,5-di-*O*-methylene galactarate (Galx). This compound is the dimethyl ester derivative of galactaric acid with the hydroxyl groups acetalized with formaldehyde, and it is readily synthesized from the commercially available mucic acid. Mucic acid is in turn obtained from the carbohydrate galactose and can thus be traced to biobased resources. The linear carbohydrate-based polyester acetals were synthesized via facile melt polycondensation between Galx and different 1,*n*-alkanediols. The resulting semicrystalline polyester acetals were thermally stable up to 350 °C, and showed *T*_g_ values in the range of −17 to −6 °C, while *T*_m_ values were observed between 70 and 85 °C. To analyze the effect of acetal functions on the hydrolytic degradation and enzyme-catalyzed hydrolysis, a comparative degradation study was performed on the polyester-acetal and poly(alkylene adipate), in which the acetal-based monomer was replaced by adipic acid to form a polyester.

Samples of the two polymers were first subjected to aqueous media with respective pH values of 2.0, 7.4, and 10.5 at room temperature for 2 months. The hydrolytic degradation process was evaluated over time by measuring the weight loss and molar mass of the residual polymer. The molar mass and the residual weight of the polyester remained constant over time. In contrast, the polyester-acetal exhibited a weight loss of 4% at pH 7.4 and 15% at pH 2.0 over a 55-day time period. The *M*_n_ and *M*_w_ values also significantly decreased over time. The *M*_w_ of the polyester-acetal decreased from ∼40 to 20 kg/mol and ∼12 kg/mol at pH 7.4 and pH 2.0, respectively. This indicates that the hydrolytic degradation of the polyester-acetal was significantly accelerated due to the presence of acetal-groups. The polyester-acetals were also incubated for 21 days at 37 °C in aqueous medium with pH 7.4 containing the Amano lipase from *Pseudomonas fluorescens* or lipase from porcine pancreas. In this case, both the polyester and polyester-acetal degraded, but the weight loss and decrease in molar mass were more significant for polyester-acetal, indicating that the acetal groups attribute to a greater degree of degradation. Control experiments confirmed that the presence of enzymes accelerated the degradation process.

In a similar study, bicyclic acetalized carbohydrate-based monomers from galactaric acid were incorporated in the polymer backbone of poly(butylene terephthalate) (PBT) by replacing the diol (1,4-butanediol) or diacid (dimethyl terephthalate) monomers with the acetal functionalized monomers 2,3:4,5-di-*O*-methylene-galactitol and dimethyl 2,3:4,5-di-*O*-methylene-galactarate, respectively.^205^ The synthesis took place via facile melt polycondensation at temperatures between 160 and 240 °C. Hydrolytic degradation experiments were carried out by immersing samples in an aqueous solution at pH 2.0 and 80 °C for 55 days. For a copolymer where half of all diol units were replaced by an acetal-based monomer, a weight loss of 18% was observed together with an appreciable decrease in *M*_n_ and *M*_w_. These decreases in molar mass and residual weight became more apparent with the increasing acetalized galactaric unit content in the polymer main-chain, showing that the hydrolytic degradation is significantly enhanced by the presence of acetal functionalities. Interestingly, enzymatic degradation did not result in significant decrease in molar mass.

The ability of the acetal linkage to undergo hydrolysis under mild acidic conditions could potentially accelerate the susceptibility to degradation under different environmental conditions, for example, in seawater. This was demonstrated by the incorporation of acetal groups into the main-chain of polylactic acid (PLA) via ring-opening copolymerization of lactide and 1,3-dioxolan-4-one (DOX).^206^ The incorporation of DOX did not affect the *T*_g_ of PLA but it did decrease the *T*_m_ from 174 °C for PLA homopolymer to 116 °C for the polyester-acetal with the highest (36%) acetal incorporation. The degradation of PLA and polyester-acetal with 4% DOX content was assessed in aqueous media with pH values of 1.0, 5.0, 7.0 (distilled water) and 7.5 (seawater, with a reference to Atlantic Ocean, pH = 7.5–8.1) over a time period of 45 days. The PLA homopolymer showed no measurable changes under these conditions. For the polyester-acetals, the maximum weight loss was 2%, while the molar mass decreased by 35% at pH 1 and by 15% in simulated seawater. In addition, some surface erosion was observed. Even though the weight loss was insignificant, the clear reduction in molar mass indicates that the presence of acetal functionalities in the PLA backbone facilitates the water degradability. This also agrees with the typical hydrolytic degradation process starting by molar mass reduction, which is later followed by weight loss due to formation of water-soluble products.^207^

Some studies also showed that dithioacetal and dithioketal^208−210^ functionalities can facilitate (bio)degradation under oxidative conditions in the presence of reactive oxygen species (ROS). Since prevalence of ROS is increased in certain cells (i.e., cancer cells), ROS-mediated (bio)degradation of polythioacetals and polythioketals have been mainly used in biomedical applications such as controlled drug delivery systems. In this regard, a biodegradable and ROS-responsive poly(ester-thioacetal) was designed for antitumor drug delivery applications.^211^ The poly(ester-thioacetal) was synthesized by polycondensation reactions methoxy poly(ethylene glycol) (mPEG) and 1,6-hexanediol, together with a diacid linker containing a thioacetal functionality, obtained from the reaction between cinnamaldehyde and 3-mercaptopropionic acid. To investigate the ROS-mediated degradation of the obtained poly(ester-thioacetals), the polymers were incubated in aqueous H_2_O_2_ (250 mM) for 0, 6, and 24 h. ^1^H NMR results showed that characteristic thioacetal bonds slowly disappeared over time, indicating its oxidative cleavage of the thioacetal groups. The oxidative degradation of thioacetal and thioketals have not been further studied in terms of degradable polyesters, however, similar results were obtained for polyurethanes containing thioacetals and thioketal groups.^212^

Thus, when incorporated in the main chain of a polymer the acetal groups show great potential in imparting polymers with higher susceptibility to degradation under environmental conditions, while the polymers still maintain good thermal stability and mechanical properties. However, so far mainly hydrolytic degradation (including acid- and enzyme-catalyzed processes) was investigated, while studies on biodegradation and/or composting are lacking. More research is, therefore, needed to evaluate and define the potential of this approach for accelerating biodegradation under different simulated and real environmental conditions.

### 5.4 Polyesters with Photoreversible Bonds

**Figure 21:**
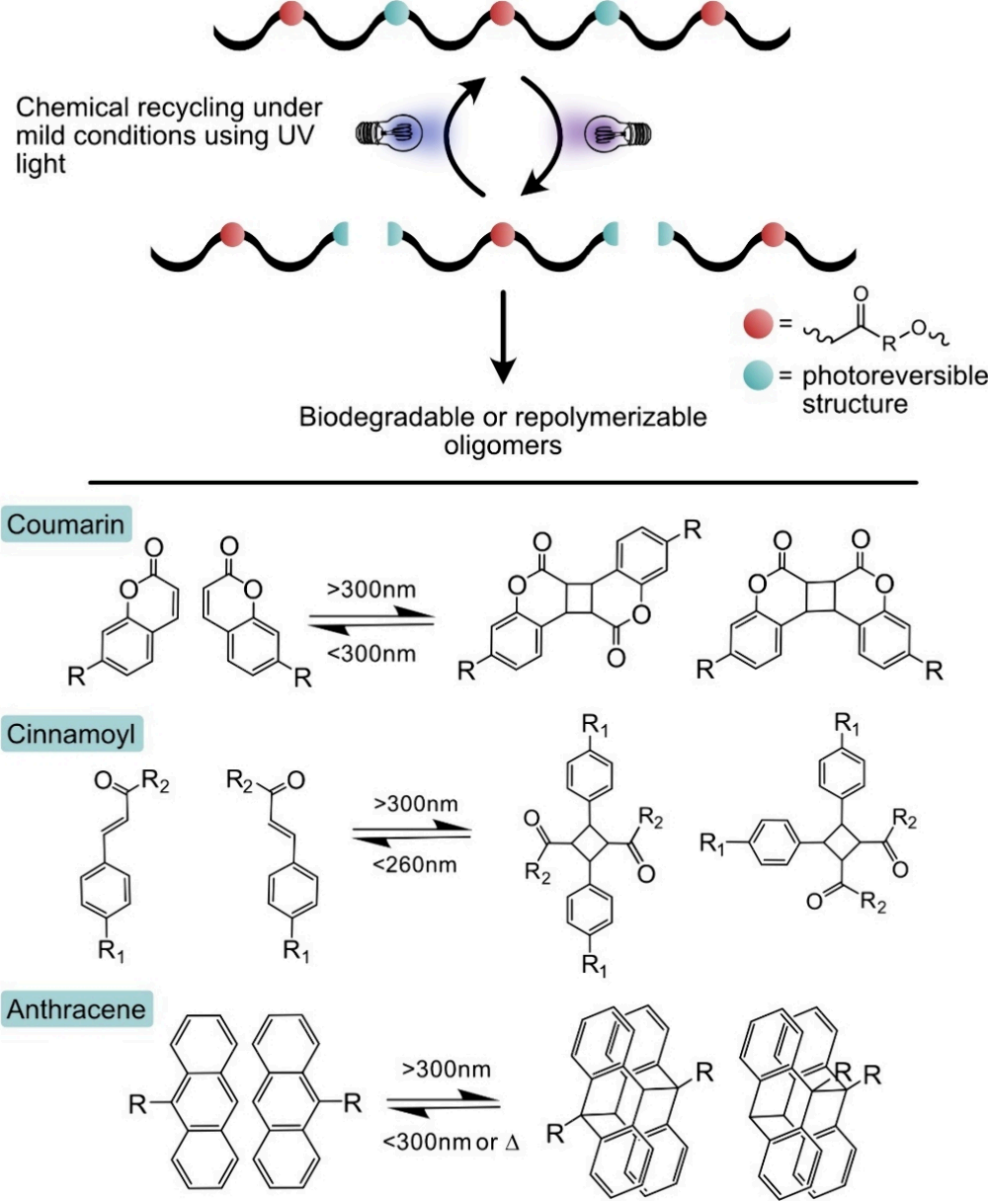
Utilization of photoreversible groups in the polymer chain to chemically recycle polymers to oligomeric products that can be repolymerized in closed-loop or potentially biodegraded. Examples of typical photodimerization reactions for coumarin, cinnamoyl, and anthracene, two common photoreversible groups.

Modification of polyesters with photoreversible structures provides potential for facile chemical recyclability and potentially also enhanced biodegradability through cleavage to monomeric or oligomeric structures. These structures can then either be reformed into new product or be more easily further biodegraded compared to high molar mass polymer. Photopolymerization, defined by De Schryver et al. in 1971 as a process where each propagation step results from a photochemical reaction,^213^ offers a way of implementing reversibility into a material under mild conditions. Some photodimerizable compounds offer photoreversibility through bonds that can be formed and cleaved back to the original structures by irradiation of light of specific wavelengths (Figure 21). Most commonly, reversible photodimerization is observed for conjugated molecules, such as coumarin, cinnamic acid, or anthracene, which are all capable of forming dimers through [4π + 4π] or [2π + 2π] cycloaddition reactions when irradiated with UV light.^214^ Here, we will give examples of utilization of coumarin, cinnamoyl, and anthracene, three common photoreversible compounds, to design more circular polymer materials.

Cinnamic acid is a biobased and biocompatible compound that can be isolated from cinnamon bark and other biomass resources. The conjugated vinyl structure of cinnamic acid allows the compound to dimerize under UV light, forming a cyclobutene structure in one of two possible conformations.^215^ By performing substitutions of the carboxylic acid unit, a range of different cinnamoyl derivatives can be achieved, the most common being cinnamate or cinnamamide. By changing the substituents, the light required for dimerization and cleavage will also change, but generally, light in the region of 260–300 nm is used. One of the possible derivatives of cinnamic acid is coumarin, another biobased and biocompatible structure that can photodimerize.^216^ Upon irradiation of light above 300 nm coumarin dimerizes through the formation of a cyclobutene structure in one of four possible conformations; *syn* head-to-head, *syn* head-to-tail, *anti* head-to-head, or *anti* head-to-tail.^217^ The reversible cleavage requires high energy light, most often a UV lamp at 254 nm is used, which can sometimes lead to damage to the surrounding material matrix as well as side reactions. Anthracene can go through a [4π + 4π] cycloaddition upon irradiation of light above 300 nm. Photocleavage of dimers can be achieved using either high-energy light with a wavelength below 300 nm or through thermal dissociation at temperatures in the range of 100–200 °C, depending on the substituents.^218^ Cleavage of the dimers through thermal dissociation generally allows for a more efficient reversal of the dimer structure, but it also puts limits on the thermal stability of materials. In addition, anthracene is sensitive to irreversible side reactions as it reacts to form stable endoperoxides, when irradiated with UV-light in the presence of molecular oxygen.^219^

With the use of UV lamps irradiating at highly specific wavelengths, a high level of control can be achieved for both dimerization and cleavage reactions. So far, this targeted reversibility was mainly implemented in biomedical^220^ and energy applications.^221^ Within polymer science, photoreversible structures have been used to create stimuli-responsive materials that can change properties upon changes in the environment.^222^ The use of photoreactive structures offers greener reaction conditions ideally without need to add initiators or catalysts. This could further offer a route to greener polymer circularity through de- and repolymerization at ambient temperature.

#### 5.4.1 Crosslinked Polyesters

Implementing photoreversible structures at the end of polymer branches could enable crosslinked networks that are reversible back to linear thermoplastic structures, i.e., recyclable thermosets. This concept has already been used for the development of self-healable thermosets with tunable material properties.^223,224^ As an example, photoreversibly crosslinked polyurethane networks based on PCL diols, triols and tetrols, 1,4-butanediol, and either hexamethylene diisocyanate (HDI) or isophorone diisocyanate (IPDI) were synthesized and functionalized with coumarin units.^225,226^ Irradiation of the films with 354 nm UV light led to approximately 90% dimerization yields after 30 min. This was determined to be the optimal light region, as shorter wavelengths (313 nm) led to irreversible side reactions and longer wavelengths (365 nm) required significantly longer reaction times. The reverse photocleavage reaction was performed using light at 254 nm and led to 75–80% conversions back to nondimerized structures. Repeating the cycle of photodimerization followed by photocleavage showed that the reversibility remained even after nine cycles, although with a decreasing yield for every cycle. Losses in reversibility were noted to occur mainly during photocleavage when irradiating with high energy light. In terms of mechanical properties, samples showed tensile stress values that increased from the initial values around 1–2 MPa to 2–6 MPa. In the same way, strain to failure was reported to increase significantly after irradiation, from initial values at 40–70% up to approximately 100–650%.

Pendant amidocoumarin groups were implemented into either soft (PCL) or hard (IPDI) regions of polyurethane networks using a short diol linker.^225^ It was shown that the position of pendant amidocoumarin groups impacted the photoreactivity and photoreversibility of the polyurethane networks. Having the amidocoumarin units in the soft segments led to slower dimerization kinetics compared to when they were incorporated in the hard segments (90 min versus 60 min), while the opposite was observed for the reversed reaction (90 s versus 6 min). Placement of the amidocoumarin units in hard regions also resulted in overall less photoreversible material, with lower recovery yields after each cycle of dimerization and cleavage, which was attributed to the reduced mobility of coumarin units.

Photoreversible groups have also been implemented to develop reusable and self-healing thermosetting adhesives.^227,228^ The utilization of photoreversible bonds instead of thermoreversible bonds offers advantages in terms of energy efficiency, targetability as well as wider useable temperature range.^229^ There is still limited work on combining polyester-based adhesives with photoreversibility. However, end-functionalization of epoxidized soybean oil (ESO) with coumarin units lead to self-healable and thermally stable adhesives.^230^ Incorporation of flexible ester linkers between the ESO backbone and coumarin units led to more efficient dimerization and photocleavage reactions with 75–80% yield compared to 36% yield for the sample without a linker. The reversibility of adhesive properties was tested by adhering two quartz slides together and by repeatedly irradiating the joint with either 365 or 254 nm light. The strength of the adhesive was determined to be greater than for previously reported similar UV-curable adhesives, with a maximum measurable lap-shear strength of 3.1 MPa. Cyclic cleavage and repolymerization of the material gave a gradually decreasing adhesion for each cycle (94% recovery for first cycle). This was explained by photochemical equilibrium and side reactions during photocleavage.

Other studies implemented anthracene moieties as the photoreactive structures for reusable adhesives,^231^ which enabled reversibility using either UV irradiation or heating. Anthracene has also been reported to have higher activity for cycloaddition compared with coumarin as well as a better processability.^232^ As an example thermosetting polymer network based on PCL-diol, 1,4-butanediol and 4,4′-methylene bis(cyclohexyl isocyanate) was functionalized with pendant anthracene units.^233^ The incorporation of PCL units led to a partially crystalline material with *T*_g_ values below room temperature. This led to higher adhesive strength, with a lap-shear strength up to 7.5 MPa. To design a system that was flexible enough for efficient crosslinking and decrosslinking, pendant anthracene units were implemented using flexible 2-mercaptoethanol linkers. However, it was concluded that more research is needed to achieve a more reversible polymer network.

Biocompatible polyesters with photoreversible groups have also been synthesized for use in drug delivery systems.^234,235^ Coumarin-containing polyesters were synthesized by utilizing 7-(hydroxypropoxy)-4-(hydroxymethyl)coumarin as a diol, stepwise polymerization was performed with adipic acid, sebacic acid, or Boc-l-glutamic acid. The resulting polyesters underwent reversible crosslinking and decrosslinking by irradiation at 350 and 254 nm light, respectively, similar to what has been shown for other coumarin-containing polymer networks. The coumarin units in the main polymer chain also led to irreversible chain scission at the 4-position, when polymers were irradiated with UV light. For irradiation at 254 nm, chain scission was highly efficient and led to an irreversibly degraded material. However, during irradiation at 350 nm, the efficiency of the chain scission was shown to be highly dependent on the state of the polymers. Solid-state films favored reaction through crosslinking and chain scission was favored when the polymers were dissolved in CHCl_3_.

Utilization of photoreversibility to facilitate biodegradation of materials is a field that has not yet been widely explored. Most often, photoreversible units are implemented as a way of achieving more control in smart materials, which are not designed for complete degradation. A few studies have, however, looked into functionalization of materials already known to be degradable, such as PCL or PLA, with photoreversible units.^236−238^ For example, one study used poly(ester urethanes) functionalized with cinnamoyl units to achieve shape memory materials with tunable degradability.^239^ Polyesters were synthesized through polycondensation with diester cinnamoyl derivatives together with diols and end-functionalized with methacrylate units through urethane linkages before thermal crosslinking. By altering the structure of either the cinnamoyl derivative or the diol used in polycondensation, a range of different thermal and mechanical properties could be achieved, with *T*_g_ ranging from −13 to 52 °C and Young’s modulus from 0.4 to 61 MPa. Degradation studies were performed in phosphate buffer saline at pH 7.4 and 37 °C. Mass losses of 30–40% were reported after 40 days of hydrolysis. Shape memory was programmed by irradiating the polymer films with 302 nm UV light, while stretching. The shape could then be reversed by irradiation with 254 nm light. Thermal shape memory could be programmed by heating and shaping networks at temperature above respective *T*_g_. This means that multishape memory networks were achieved, where the shape could be altered by two different triggers.

#### 5.4.2 Linear Polyesters

Incorporation of photoreversible groups within the main polymer chain allows for the synthesis of reversible structures that can be broken down to low molar mass compounds under controlled conditions. This could potentially enable efficient chemical recycling of plastics under mild conditions. Early work on linear photoreversible polymers often focused on implementing high concentrations of photoreversible groups, leading to highly photoreactive polymer structures.^213,240^ However, this generally resulted in high degree of degenerates (in some cases >50%) for each cycle of cleavage followed by redimerization.^241^ More recent work in this area therefore focused on chain scissions leading to oligomeric structures.

Different factors determining the structural features of dimers, including both competition between different conformations, as well as competition between *inter*molecular versus *intra*molecular dimerization, have been investigated.^240^ Short-chain polyester linkers of varying lengths were end-functionalized with coumarin derivatives to study their dimerization behavior.^228,232^ By incorporating a methyl substituent on coumarin, both the stereochemistry and photoreactivity could be controlled. Using unsubstituted structures was shown to lead to a preferential head-to-head conformation of dimers, while the methyl substituent led to exclusive formation of only head-to-tail conformation. A similar study using short-chain polyethers also showed that the unsubstituted dimerization reaction gave a higher yield compared to substituted structures (79% versus 66%). However, as the linker of the substituted dimers was slightly weakened due to steric hindrance, the resulting material was shown to be more easily photoreversible.^241^ In terms of the length of the polyester linkers, dimerization tests showed that using shorter linkers favored intramolecular cyclization, while longer linkers instead favored polymerization. This allows for a straightforward way of designing photoreactive materials for different applications. How to favor the intramolecular reactions during photodimerization has been further investigated in several studies since then.^242−245^ As an example, PCL was end-functionalized with anthracene units to synthesize reversibly cyclic PCL (cPCL) of high molecular weights and variable ring sizes.^246^ By varying the concentration of end-functionalized PCL in solution during irradiation, rings of different sizes could be synthesized. Reversibility of the cPCLs was tested through thermal dissociation of anthracene units at 160 °C, showing a clear decrease of molecular weights. The degree of dissociation was however reported to be low, leading to yields of approximately 50% of cleaved anthracene structures.

Alternating associative polymers (AAP) containing anthracene units were developed to synthesize ultrahigh molecular weight photoreversible polymers.^247^ A hydrophilic PEG backbone was end-functionalized with hydrophobic chain-ends using HDI and ester-linked anthracene to form telechelic associative polymers (TAP) with a molecular weight of 23.5 kg/mol. Dissolving TAP in water led to the formation of micelles where anthracene units were closely packed together in the hydrophobic core. Irradiation with 365 nm light led to very efficient dimerization conditions, and polymers with molecular weight over 850 kg/mol were achieved. For polymer solutions containing 2 wt % TAP, UV irradiation led to a clear phase transition, from a low viscosity system to a gel. This was reported to be due to bridging of some polymer chains across several micelles. This created a physical network of AAP, which was unable to relax in finite time. The photoreversibility of this system was also shown to be high, with yields close to 100% after five cycles of irradiation followed by heating at 150 °C. A similar system and gel formation was also reported starting from coumarin.^248^ However, because of the limiting reactivity of coumarin with dimerization plateauing at approximately 80% compared to 99% for anthracene, the system was unable to reach the same high molecular weight.

The mild reaction conditions needed for photodimerization, requiring no catalysts, additives, or heating, can be utilized for synthesis of novel circular polymers. In this context, polyesters containing pendant perfluorophenyl were synthesized.^249^ A perfluorophenyl diol was difunctionalized with 4-(anthracen-9-ylmethoxy)-4-oxobutanoic acid and photopolymerized under 365 nm light to form polymers with number-average molecular weight in the range of about 8000 Da. Through a postfunctionalization step, the perfluorophenyl unit could then be exchanged with a variety of different amines, such as furfuryl amine, benzyl amine, and propargyl amine, under mild reaction conditions. The reaction resulted in good yields of up to 100%. The photoreversibility of the system was, however, not evaluated.

The noncomplete reversibility after photocleavage and photodimerization is a continuous challenge for the development of photoreversible materials. This is often attributed to a combination of side reactions, resulting in irreversible structures as well as equilibrium between dimerized and cleaved structures.^230^ To increase the reversibility, several studies investigated the relationship between substituents, substituent positions, and resulting photoreactions. As an example, the effect of using different substituents as linkers at the 9-position of anthracene^250^ respective 2,6-substitutions was evaluated.^251^ Changing the linker was, however, reported to have modest effect on the photodimerization kinetics while changing the concentration and light intensity had stronger impact.

For thermal cleavage of dimers, the choice of linker had a more significant impact. Linkers consisting of methyl esters led to highly stable dimers which needed temperatures in the range of 160–180 °C to thermally cleave, while dimers with linkers attached through ether bonds only needed temperatures in the range of 100–120 °C to cleave. Other linkers, such as longer alkane chains and esters formed dimers of intermediate stability, being placed between these two extremes. Regarding substitution position, an electron-withdrawing ester was synthesized from an anthracene derivative containing a 2,6-substitution of an electron-donating methoxy group. This structure showed faster dimerization kinetics under 365 nm light, with 98% conversion after 1 h versus 43–82% conversion for anthracene dimers substituted at the 9-position. The resulting dimers from 2,6-substitution had higher thermal stability compared with 9-substituted ones and a temperature of at least 180 °C was needed to cleave the structures efficiently. For coumarin, the problem of noncomplete reversibility is even more prevalent. Especially during photocleavage, when high energy UV light is needed, irreversible bonding and equilibrium reactions affect the final yield achieved. A recent study using intramolecular dimerization of pendant coumarin units showed that the reversibility of the system could be increased by irradiating the material at lower temperatures while also using a rigid polymer matrix.^252^ By using a rigid PMMA backbone and irradiating at 10 °C, a high degree of reversibility was shown even after seven cycles. However, losses of around 20% were still seen after the first cycle, which were attributed to depletion of internal material strains as well as possible side reactions.

There are still many knowledge gaps in understanding the photoreversible reactions and optimization of photoreversible materials. Ideally, photoreversibility could offer an efficient way of making more environmentally friendly thermoplastics and thermosets that are recyclable under mild conditions. Combining photoreversible bonds with dynamic ester bonds could be a promising route to materials with facile chemical recyclability and biodegradability. The main limiting factor for photoreversible material is still the noncomplete reversibility and different side reactions. More work is required to understand and optimize the reversibility by design of linkers, substituents, and reaction conditions.

## 6 Additives to Catalyze Degradation of Polyesters

As an alternative strategy to tailor the end-of-life behavior and fate of materials, additives can be incorporated in the polyester matrix to trigger the degradation under specific conditions. Ideally, these additives do not change the material properties and processability of the product, while they can effectively initiate and ensure the degradation of the material after service. The possibility to accelerate the biodegradation of polyesters by inclusion of plant fibers was recently reviewed and will not be further discussed here.^253^ Instead, we will briefly discuss chemical or biological catalysts as additives that can accelerate the hydrolytic degradation rate when the material comes in contact with aqueous environments, soil, or compost. We will start by visiting photocatalytic additives that can trigger the degradation when the material is subjected to sunlight or UV irradiation.

### 6.1 Chemical Catalysts

Photocatalytic particles have been utilized to modulate and accelerate polyester degradation. As an example, nanocomposites comprising of 5 wt % of titanium dioxide (TiO_2_) particles and biodegradable PBS were produced using high-shear extrusion.^254^ TiO_2_ particles were well-dispersed within the PBS matrix, and the material maintained its mechanical strength compared to neat PBS. When subjected to simultaneous UV irradiation and Amano Lipase PS from *Burkholderia cepacia*, the TiO_2_/PBS composites were more readily degraded compared to TiO_2_/PBS or PBS subjected to photocatalytic oxidation or enzymatic hydrolysis alone. Indeed, the enzymatic degradation rate of TiO_2_/PBS was lower than what was observed for pure PBS. This was explained by inhibition of enzyme adsorption onto PBS surface caused by the TiO_2_ particles. In this context, the weight loss of the TiO_2_ composites subjected to a 3-day treatment by both UV irradiation and enzymes was ∼44% higher compared to the weight loss of neat PBS under same conditions and over a similar time period, showing that the photocatalyst accelerated the degradation rate. In the same frame, the degradation of PHB films containing immobilized nanosized TiO_2_ was assessed.^255^ The photocatalytic activity of TiO_2_ was preserved in the PHB–TiO_2_ composite, as demonstrated by efficient photocatalytic decolorization of methylene blue and sterilization of *Escherichia coli*. The effect of TiO_2_ on biodegradation of PHB was found to depend on the soil microbial activity and submission to UV irradiation. The composite films showed faster degradation over a duration of 43 days when exposed to direct solar illumination at soil surface, indicating photocatalytic influence, while buried films exhibited slower degradation rates. The photoactivity of the composite might, however, reduce the viability of the microorganisms on the material surface, which may present a trade-off between photocatalytic degradation and biodegradation. In a recent study, poly(3-hydroxybutyrate-*co*-3-hydroxyhexanoate) (PHBH) was coated with cellulose triacetate (CTA) to prevent degradation of PHBH during use.^256^ Interestingly, deacetylation of CTA could take place under slightly alkaline conditions in seawater, which at the point of collection had pH = 7.7 and temperature ∼15 °C, and the released acetic acid could catalyze the hydrolytic degradation of PHBH.

Ferric chloride is a fairly strong Lewis acid, conventionally used as a catalyst in organic synthesis.^257^ Recently, the scope of embedded chemical catalysts was expanded by studies on PLA composites with embedded ferric chloride (FeCl_3_).^258^ This modified PLA with 3% FeCl_3_ (expressed as parts per hundred resin) exhibited a degradation rate over 10 times faster than pure PLA in alkaline (10% sodium hydroxide) aqueous solution. The suggested mechanism of action of FeCl_3_ involved formation of iron(III) complexes with the C=O of the ester group, weakening the ester bond and accelerating the PLA degradation. The presence of FeCl_3_ also led to a marked reduction of crystallinity and a drop in the thermal stability during processing, leading to reduced molar mass of PLA. In a follow-up work, the same group used incorporated FeCl_3_ in PLA/PBAT by melt blending to overcome slow degradation rate of PLA alone.^259^ This resulted in materials with good mechanical properties and higher susceptibility to degradation. For instance, when subjected to a hydrolysis test under alkaline conditions, the 9-day weight loss from PLA with FeCl_3_ was ∼45%, while this value was ∼90% from PLA–PBAT blends with various FeCl_3_ contents. However, the suitability of polyesters with added inorganic substances to recycling or their potential ecological impacts have been poorly validated to date.

### 6.2 Biological Catalysts: Polyesters with Embedded Enzymes

Microbial enzymes play a crucial role in the depolymerization of polyesters under environmental conditions. Microbial enzymes have an ability to catalyze a wide range of reactions, making them useful biotechnological agents in technical formulations. In order to enhance the biodegradation rate of slowly biodegradable polyesters hydrolytic enzymes derived from microorganisms have been integrated into the polyester matrix.^260^ Furthermore, the embedded enzymes could offer the possibility to ensure degradation of materials even when they unwantedly end up in environments that are not favorable for (bio)degradation. This integration has been achieved through solution casting or melt processing of, e.g., surfactant-coated or immobilized enzymes. It is evident that the incorporation of enzymes into polyesters presents opportunities, but there are also still many challenges involved as briefly summarized in Table 1.

**Table 1:** SWOT Analysis of Enzyme Embedded, Designed-to-Degrade Polyesters

strengths	weaknesses
– Enhanced degradation of polyesters disposed in the environment to prevent accumulation of plastic waste	– Requires protection of enzymes from denaturation caused by temperature, organic solvents, and polymer additives; some enzyme activity still typically lost
– Degradation can be initiated even in less favorable environments	
Enzyme Type, Loading, And Distribution Influence Degradation Process	
– Tailored degradation under specific conditions matching enzyme’s pH and temperature optima, which could be utilized for green recovery of chemicals	– Hydrophobic nature and crystallinity can impede enzyme-catalyzed hydrolysis
	– Possible steric and ionic hindrances

opportunities	threats
– Progress in incorporation of enzymes, i.e., new green solvents	– High loading and costs of enzymes can impede commercialization
– Discovery of new biotic enzymes	– Properties do not meet performance criteria
– Enzyme engineering and artificial enzyme mimics	– Degradation starts already during service-life
	– Enzymes are released from the degrading material and degradation stops or slows down

Most polyesters are rather hydrophobic in nature.^105^ While this hydrophobicity is usually a favorable property during the service life, it typically has an opposite influence on the susceptibility to both chemical hydrolysis and biodegradation.^27^ The term embedded enzymatic degradation was coined by Ganesh and co-workers who solution-casted films of surfactant coated CALB and PCL to enhance its biodegradation rate.^260^ While a complete enzymatic hydrolysis of PCL with 1.6 wt % of embedded CALB took nearly 17 days, the reaction run to completion within 24 h when the PCL contained 6.5 wt % of embedded CALB. Authors proposed that this approach increased the enzyme–polymer interactions and changed the degradation mechanism from surface to bulk degradation. In contrast, when the enzymes were added externally, it was found that the hydrolysis was restricted to outer surfaces of the polymer material. These results were promising but also induced the need for further development as the high concentration of enzyme required would be both costly and influence the material properties.

Following this approach, polyesters with 2 wt % of nanodispersed enzymes were made by solution casting with dissolved PCL and PLA.^261^ Authors proposed that when enzymes are restricted to a nanoscale environment, polymer chain ends become accessible as the main pathway for processive depolymerization. The enzyme-loaded PCL and PLA underwent near-complete depolymerization in soil, compost, and household tap water. Once the enzyme-embedded polymer reached a level of disintegration so that the particle size was within the microplastic domain, the embedded enzymes continued to catalyze the hydrolytic degradation, achieving polymer-to-small molecule conversion of up to 98% within 24 h in aqueous buffer solution (pH 7.2) at 40 °C. The authors showed that the maximum degradation rate is achieved close to the melting point of PCL, pointing out the well-known phenomenon of preferential degradation of amorphous areas over the crystalline domains and the beneficial influence of increased chain mobility.^262^ Polymeric enzyme protectants enabled maintaining the activity of hydrolytic enzymes at 60 °C. This temperature is, however, well below typical *T*_m_ of common polyesters such as PLA, posing hurdles to processing of materials with embedded enzymes by commercially viable melt-processing methods.

**Figure 22:**
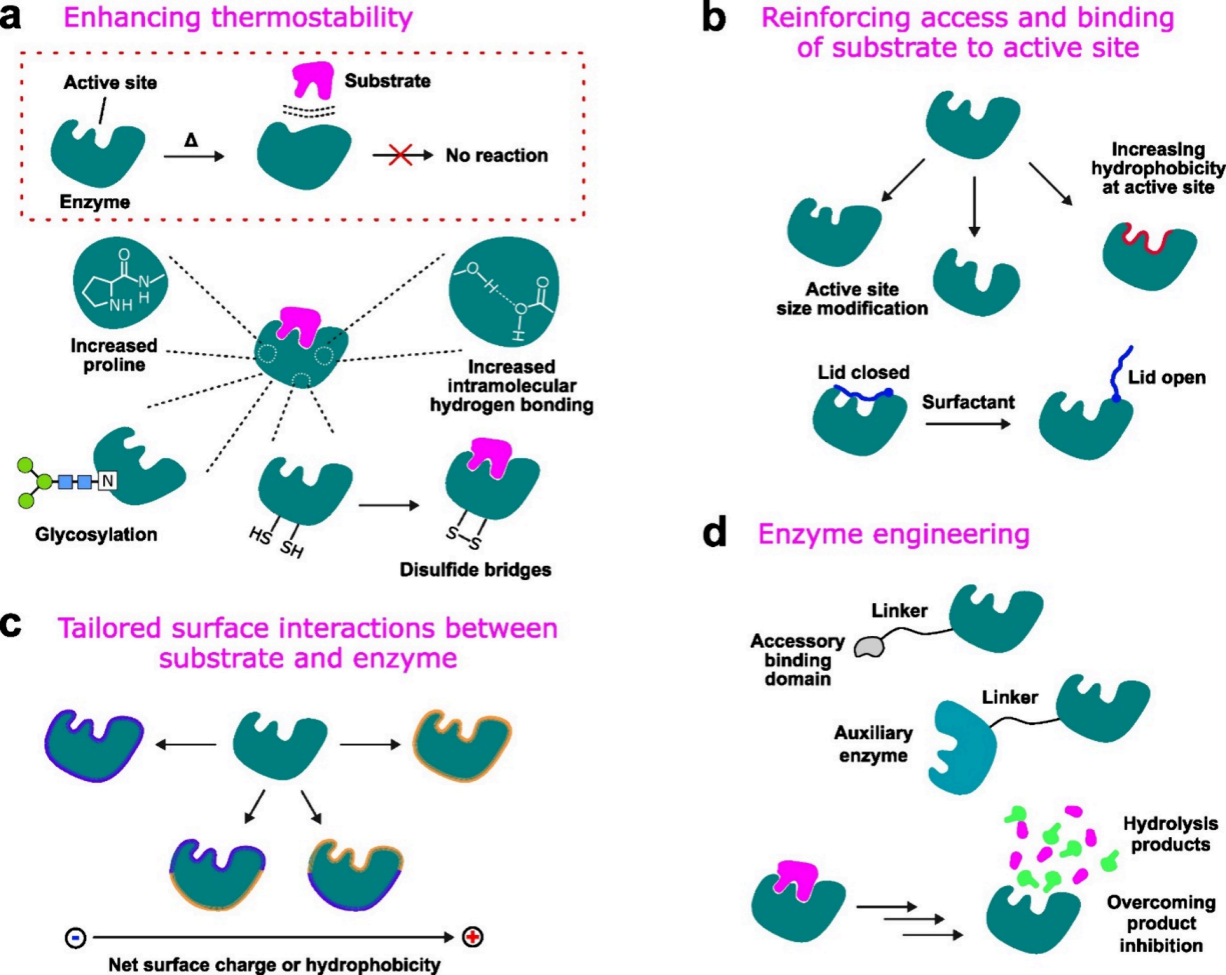
Strategies to improve enzymatic hydrolysis of polyesters. (a) Enhanced thermostability, (b) facilitated access to active site, (c) surface modifications toward improved interactions between E–S, and (d) enzyme engineering via attachment of binding domains, auxiliary enzymes, or generation of variants that mitigate product inhibition.

Incorporating heat sensitive enzymes into polyesters through more industrial solvent-free melt processing poses a significant hurdle, particularly as most polyesters have melting points exceeding 100 °C. Overcoming this challenge is, however, crucial for the success of this approach considering the widespread commercial application of melt-processing techniques in polymer manufacturing. Enhancing thermostability of enzymes is thus a logical starting point in this endeavor (Figure 22). As an alternative, an attempt was made to physically protect lipase by entrapping it in alginate beads prior to embedding the enzyme in PBS films by hot-pressing. However, the enzyme concentration and activity achieved were too low resulting in merely 5% weight loss after 78 days.^263^ In another study, polyacrylamide-immobilized proteinase K was extruded with PLLA at 200 °C to produce enzyme embedded films.^264^ This elevated temperature caused extensive denaturation of the enzyme, and only a moderate weight loss of 6% was observed after 21 days of immersion in 50 mM Tris-HCl buffer solution at pH 8.5. However, this was still a significant improvement in comparison to the weight loss of the film without proteinase K, which was practically zero during the same time interval. These results indicate partial retention of the enzyme activity during the extrusion process. In comparison, considerably higher catalytic efficiency was achieved with solvent-casted films, reaching a weight loss of 78% after 96 h with 0.5 wt % embedded proteinase K. More studies are, therefore, required to improve the thermal stability of the enzymes, either by more effective immobilization or by enzyme engineering.^265^ Furthermore, replacing poorly biodegradable immobilization matrixes, such as polyacrylamide as the encapsulant, by more readily biodegradable ones would further prevent the formation of persistent residues.

**Figure 23:**
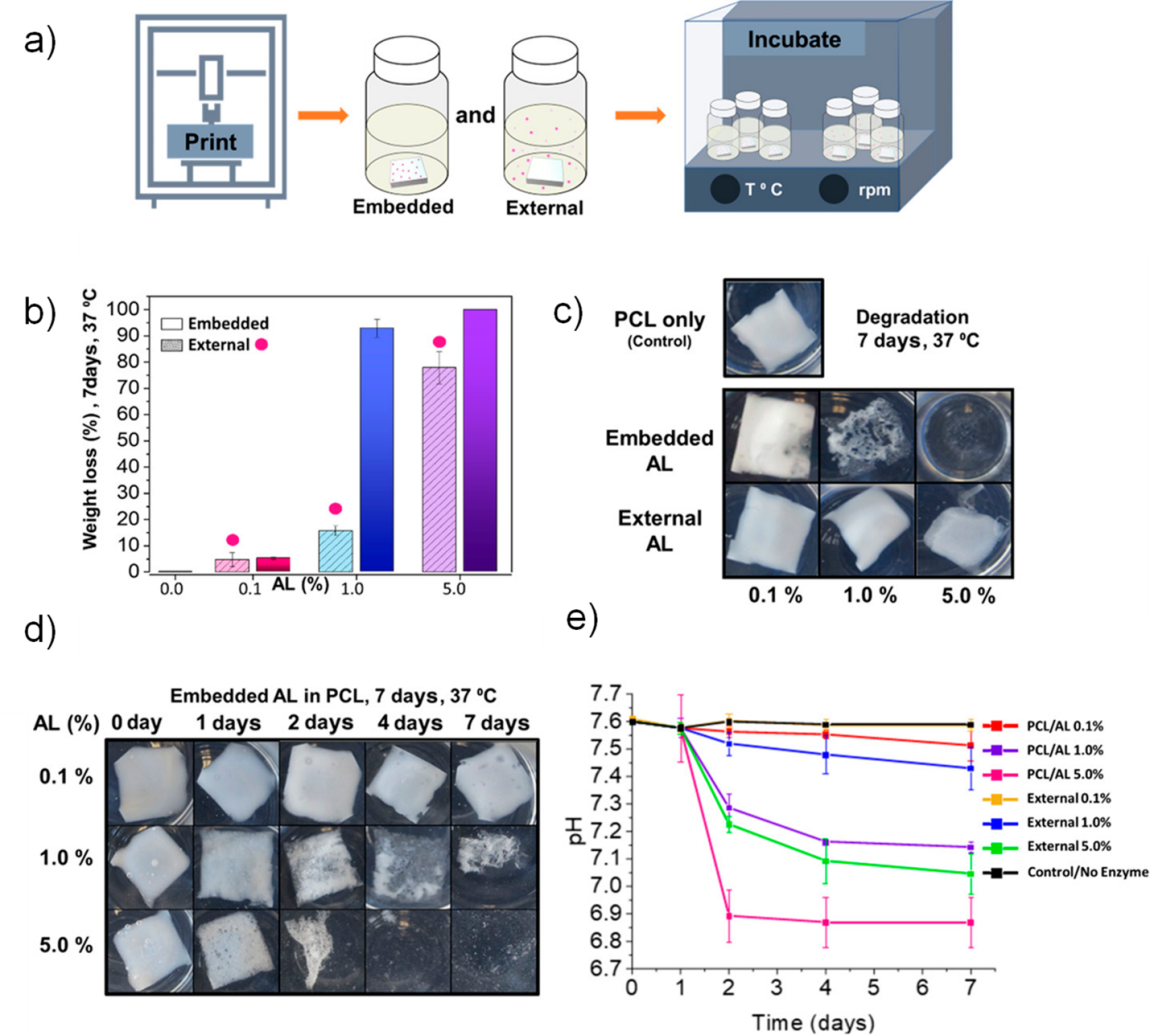
(a) Schematic over the experimental aging setup for PCL with embedded and external enzymes. (b) Comparison of the weight loss of the enzyme embedded films and the films aged with external enzymes after 7 days at 37 °C with different enzyme loadings. (c) Photographs of PCL films after 7 days of degradation without enzymes (plain PCL), with embedded or external enzymes. (d) Photographs of the PCL films with different loadings of embedded enzymes after different aging times. (e) pH of the aging medium as a function of degradation time at 37 °C. Reproduced with permission from ref (266). Copyright 2021 American Chemical Society.

3D printing based on melt extrusion technique has also been utilized to prepare PCL with embedded Amano lipase (Figure 23).^266^ Amano lipase, in the form of dry powder, was mixed with the polymer, and the resulting dry mixture was thoroughly mixed until the enzyme powder was evenly distributed throughout the polymer matrix. Interestingly, the enzyme withstood processing temperatures up to 130 °C without significant loss in activity. The 3D printed specimens with 5 wt % embedded Amano lipase were nearly completely hydrolyzed in 8 days when placed in phosphate buffer at pH ∼ 7.5. The degradation of PCL specimens by the embedded enzymes occurred mainly in the amorphous domains. This was confirmed by DSC analysis, showing 7.0% increase in crystallinity after 7 days of degradation. The SEM imaging documented the formation of holes on the surface of the specimens, and the dimensions of these holes increased over time, confirming proceeding degradation. This “internal” enzymatic hydrolysis was more efficient compared to external enzymes, i.e., when PCL was placed in aqueous solution with enzymes.

Melt extrusion process was also used to prepare PBS, PBSA, and PCL films with different lipases and the hydrolytic degradation of these films with embedded enzymes was compared to the hydrolysis of the same films with external enzymes.^267^ The influence of processing temperature on the enzyme activity and the ranking order of the three polymers based on their melt processing temperatures were investigated. PCL can be processed at temperatures as low as 90 °C, and the hydrolysis kinetics remained unaffected by the processing temperature when CALB enzyme was incorporated in PCL. In the case of PBSA processed at 100 °C, only CALB exhibited significant activity, leading to 100% weight loss within 4 days. The other evaluated enzymes experienced a drastic loss of activity, likely due to thermal denaturation during the extrusion process. Lastly, PBS required the highest processing temperature, and it was the most difficult substrate to hydrolyze with only 18% weight loss when CALB was embedded into the films at 130 °C. This efficiency was actually lower compared to the degradation with external CALB that lead to approximately 40% weight loss within 21 days. All other lipases completely lost their activities during extrusion at 130 °C. Embedded enzymes could also provide a potential solution for slow degradation of polyesters in seawater, as recently shown with thermally embedded *Humicola insolens* cutinase that accelerated the degradation of polyesters in seawater.^268^ In another recent study, the embedded enzyme approach was found effective in inducing deacetylation of cellulose acetate, which could help to overcome the bottleneck of efficient biodegradation of chemically modified cellulose esters.^269^

The aforementioned pioneering studies have pushed the boundaries of melt-processing enzymes within the temperature ranges applicable for processing of PBSA, PBS, PBAT, and PLA, with melting points ranging from 100 to 160 °C. However, other commodity polyesters such as PET, PBT, and poly(cyclohexylene dimethylene terephthalate) (PCT) still pose significant challenges due to the substantially higher melting points exceeding 200 °C. Another challenge with aromatic polyesters is their significantly lower susceptibility to enzymatic hydrolysis, meaning that chemical pretreatments and enzyme engineering are typically required to increase the efficiency.^9,270^ Here, directed evolution coupled with machine learning could provide a new tool for engineering robust and effective enzymes or artificial enzyme mimics capable of catalyzing the breakdown of different polymer structures.^271^ Improving the enzyme immobilization and enhancing the thermal denaturation resistance are pivotal in making progress in this frontier. Moreover, in nature, the biodegradation of polymers such as cellulose involves a complex interplay of microbial enzymes that exhibit synergistic processive activity. Within this domain, the benefits of incorporating enzymes working with both processive and random chain scission mechanisms was demonstrated.^272^*Candida Antarctica* (CA) lipase B and *Burkholderia cepacia* (BC), two enzymes known to degraded PCL via random chain scission and progressive depolymerization, respectively,^261^ were embedded in PCL films and demonstrated to synergistically induce a random scission in the amorphous domains and processive depolymerization. This conclusion was supported by reference samples and by means of molar mass analysis and relaxation studies by NMR. First, BC alone was immobilized on high or low molar mass support and embedded in PCL. Interestingly, these systems had completely different degradation behaviors. In the materials with low molar mass immobilization matrix depolymerization stopped after 3 h at 65% weight loss, while the other system continued to depolymerize until almost complete weight loss (98%). Since the progressive depolymerization by BC enzyme takes place by binding of the enzyme to the chain end, this difference was explained by availability of chain-ends in the two systems. Solid-state NMR analysis showed significantly slower T2 relaxation in the rigid-amorphous regions of the material where BC was immobilized on low molar mass polymer. This was explained by attachment of chain ends at the crystal–amorphous interface, which would make them unavailable for enzyme binding. However, the combination of CA and BC lipase overcame this problem as random chain scission of PCL chains in the amorphous regions provided accessible chain ends for the BC enzyme and progressive depolymerization. This process was further supported by molar mass analysis. More studies are required to fully utilize the potential of polyesters engineered for multienzyme catalyzed degradation.

In conclusion, modification of polyesters with chemical or biological catalyst is an attractive route to modulate the end-of-life fate and degradation behavior. This modification can be performed on existing commercial materials, ideally during the processing step, omitting the challenges in development and commercialization of new polymer materials. The possibility to choose both type and concentration of additives ideally gives tools to initiate the degradation of materials by specific triggers (e.g., UV-light, water) to tune and control the degradation rate in a targeted end-of-life environment and even under less favorable degradation conditions. Many challenges still exist, such as the potential influence of the additive on the degree of crystallinity and mechanical properties and risks of premature failure and degradation during processing or service. There can also be compatibility issues and difficulties in even distribution of the additives in the polymer matrix. In the case of embedded enzymes, the thermal stability of the enzymes to maintain activity after thermal processing is still one of the largest challenges, together with the additional cost of enzymes. The used additives itself should not cause any negative environmental impacts, and care should be taken not to mix these materials with those aimed for mechanical recycling.

## 7 Polyethylene-Like Polyesters As More Circular Alternatives to Polyethylene

Polyethylene (PE), with simple hydrocarbon structure, is a versatile material divided into different types, such as LDPE and high density polyethylene (HDPE), depending on the production route and degree of branching. PE materials have huge commercial, practical, and theoretical importance, but they are also among the largest contributors to plastic waste due to the cheap price, large production volumes, short-term applications, low recycling rates, and relative inertness. Biobased PE is already a commercial material but faces the same recycling problems than petroleum-based PE. Polyethylene-like aliphatic polyesters have appeared as attractive PE-alternatives. These polymers typically contain 10 or more methylene groups between the ester groups, providing the synthesized polyesters PE-like properties, tunable from flexible LDPE to more rigid HDPE by choice of starting monomers.^273^ These polyesters could become ideal circular materials of the future with mechanical and chemical recyclability and potentially also enhanced biodegradability, although it is somewhat more difficult to achieve. The synthesis and materials properties of polyethylene-like polyesters were quite extensively studied during the last years. There are also many possible biobased starting materials such as long-chain dicarboxylic acids (e.g., plant-oil fatty acids)^274^ or biobased macrolactones^275,276^ that can be utilized for the production.

### 7.1 Hydrolysis and Biodegradation of Polyethylene-Like Polyesters

After 50 years of studies, evaluating many different approaches, an easily biodegradable carbon–carbon main chain polyethylene still does not exist. Attempts to make “environmentally degradable” or biodegradable polyethylene has been made since the 1970s, including several commercial materials.^277^ These were mainly based on existing polyethylene materials modified with pro-oxidants or copolymers, where carbonyl groups were introduced into the polymer backbone or side groups. The aim was to enhance the photo-oxidative degradation of the material to break it down to smaller fragments which then would biodegrade. However, there were several problems with these materials, and the photo-oxidative degradation under environmentally relevant conditions did not continue to a degree that would allow subsequent biodegradation. The early work of Bailey introduced another approach, where weak linkages in the form of ester bonds were introduced into the polyethylene chain by copolymerizing ethylene with a ketene acetal.^278^ Biodegradation experiments indicated slow biodegradation rates for a polymer with 2 mol % of ester bonds, while the rate significantly increased for a material with 10 mol % of ester linkages. It is, however, difficult to obtain a quantitative idea of what these rates meant in % biodegradation. Also, the tested polymers had relatively low molar mass, sometimes as low as ∼5000 g/mol, which has a positive influence on the biodegradation rate but renders the material with poor material properties.

Although the synthesis of polyethylene-like polyesters has been quite extensively studied, very limited studies have been performed on the hydrolytic degradation or biodegradation of these materials.^279^ It is surprising, as they are often presented as potentially biodegradable alternatives to PE. At the same time, it can be anticipated that as the −COO–/–CH_2_– ratio decreases, the hydrolytic degradation and biodegradation rate slows down. This was clearly shown by an investigation on the hydrolytic and enzymatic degradability of polyesters prepared from pentadecalactone (C15) and hexadecalactone (C16).^280^ The hydrolytic degradation was studied during a two-year period in phosphate buffer at 37 °C, and basically no degradation was detected during this time period based on weight loss and molar mass changes. Similar neglectable degradation was observed during 100 days of enzymatic degradation in the presence of lipase from *Pseudomonas cepacia*.

Clever structural design could possibly retain the PE-like properties while increasing the susceptibility to hydrolysis and (bio)degradation. One possibility could be combining long and short monomers to introduce some more easily hydrolyzable “weak” segments. This approach was investigated by synthesizing a library of “short–long” diol–diacid polyesters.^281^ The hydrolytic degradation of five polyesters, PE2,12, PE2,14, PE2,16, PE4,14, and PE4,16 was investigated, where the first number indicated the number of carbons in the diol (C2 as ethylene glycol or C4 as 1,4-butanediol), and the second number is the number of carbons in the diacid (C12 as dodecanedioic acid, C14 as tetradecanedioic acid, or C16 as hexadecanedioic acid). The influence of the chemical structure, i.e., the length of the diol and diacid, on the hydrolysis under aqueous conditions at pH 7 and 30 or 60 °C and during soil burial was clearly observable. All the studied polyesters hydrolyzed very slowly at 30 °C, showing maximum weight loss of 2 wt % after 90 weeks. When the temperature was increased to 60 °C, the weight losses were still under 3 wt % after 48 days. However, when the hydrolysis time was increased to 72 days the influence of chemical structure became clear. Approximately 64 and 27 wt % weight losses were measured for PE2,12 and PE2,14, respectively, i.e., the materials with the shortest diol (C2) in combination with the two shortest diacids (C12, C14). At the same time, the weight loss of PE2,16, PE4,14, and PE4,16 still remained neglectable. The intrinsic viscosity, reflecting the molar mass of the materials, decreased somewhat faster compared to the weight loss, which is a typical observation during the hydrolytic degradation of aliphatic polyesters as certain molar mass decrease is required before water-soluble products are formed.^207^ The weight losses after 2 months of soil burial in moistened horticultural soil at room temperature remained under 1 wt % for all the materials. When the time was increased to 17 months, PE2,10 and PE2,12 exhibited clear weight losses of 36 and 13 wt %, respectively, while the remaining materials with longer diacids still exhibited weight losses of only 1–3 wt %. This again supports that degradation rate drastically decreases when the length of the diacid increases from C10/C12 to C14 and the short diol (C2) clearly benefitted the degradation rate. This large influence from the C2-diol can be explained by considering that most C2-diol units are expected to be located in the amorphous regions and not included in the crystalline lamella, which will significantly increase the concentration of hydrolyzable groups in the more hydrolysis sensitive amorphous regions.

**Figure 24:**
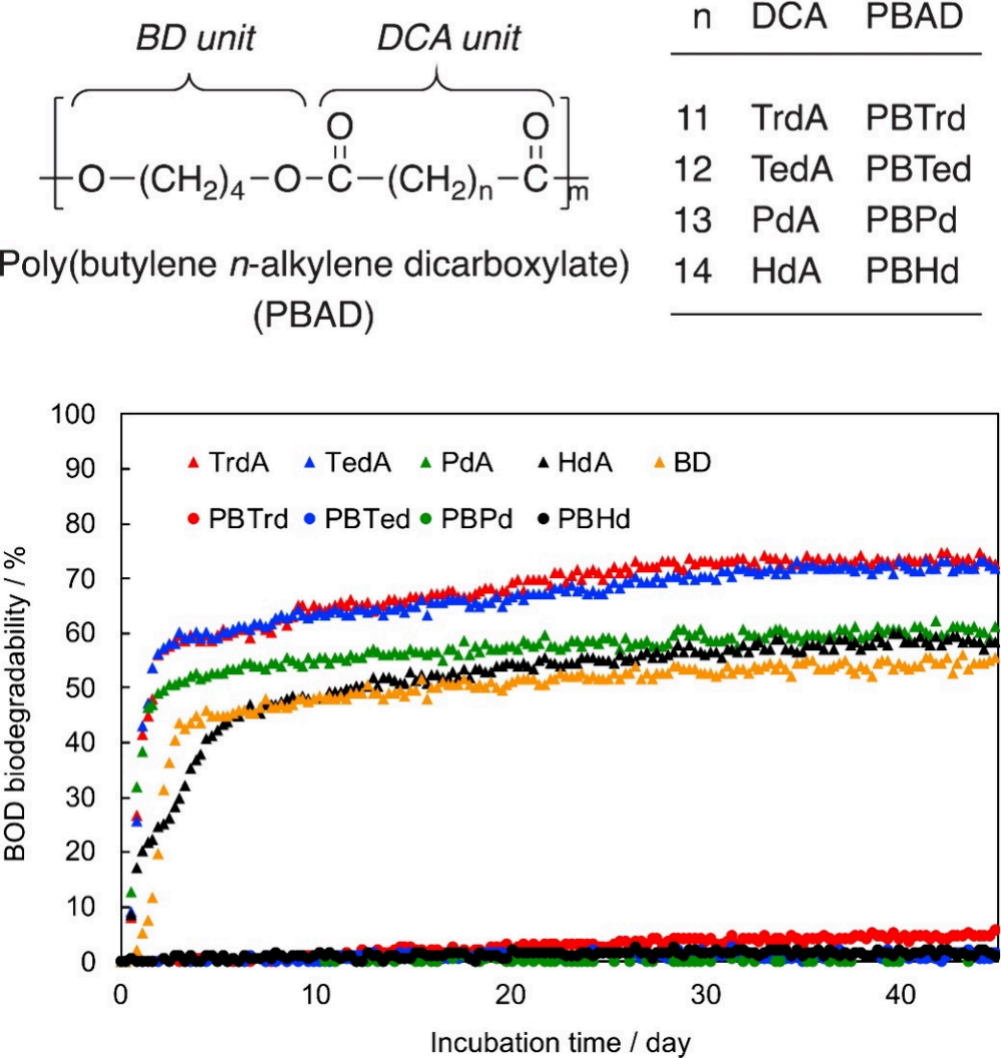
BOD biodegradability of four poly(butylene-*n*-alkylene dicarboxylates) and their corresponding monomers. Four different polyesters were synthesized by utilizing four different dicarboxylic acids [tridecanoic diacid (*n* = 11; TrdA), tetradecanoic diacid (*n* = 12; TedA), pentadecanoic diacid (*n* = 13; PdA), and hexadecanoic diacid (*n* = 14; HdA)]. In all cases, the diol was 1,4.butanediol (BD). The corresponding polymers were named as PBTrd, PBTed, PBPd, and PBHd, respectively. Adapted with permission from ref (282). Copyright 2021 Elsevier.

The crucial role of the incorporated short diol was further demonstrated, as the PE-like polyesters produced from butanediol (C4) and C11–C14 diacids did not show biodegradation during BOD-test with aerobic microorganisms from soil suspension in aqueous medium at 25 °C (Figure 24).^282^ On the other hand, the used monomers, butanediol and different dicarboxylic acids, were 56–75% biodegraded under same conditions after 45 days, giving positive indication that the polyesters could still be degraded if molecular weight can be significantly reduced by abiotic hydrolysis.

Finally, PE2,18, with HDPE-like solid-state structure and tensile properties, was mineralized to >95% during two months at 58 °C under simulated industrial composting conditions according to ISO 14855-1.^283^ This was again explained by the short diol in combination with the higher temperature and more favorable conditions compared to those in the soil burial test. As a comparison, PE18,18 only mineralized to 30% during the same period of two months. As already explained above, the introduction of the shorter diol was proposed to lead to a higher ester group concentration in the more accessible amorphous regions, which could explain the significant increase of the degradation rate. This work also evaluated the enzymatic hydrolysis of PE2,18 by two cutinase, *Humicola insolens* (HiC) and *Aspergillus oryzae* (AoC), and one lipase, *Thermomyces lanuginosus* (TlL). Both cutinases showed significant activity toward PE2,18. HiC could completely hydrolyze the materials within a few days at 37 °C, while >60% was depolymerized after 7 days at 25 °C. In comparison, PE18,18 showed almost no hydrolysis after 7 days with HiC. The other cutinase AoC depolymerized 55% of PE2,18 within 7 days at 37 °C, while TIL demonstrated almost no activity. Through modeling of the active site surface area of the enzymes, the results were explained by different accessibility of the active sites. The active site accessibility may thus be a controlling factor in the ability of an enzyme to hydrolyze crystalline polyesters.

Blending with more easily hydrolyzable components can also be utilized to increase the hydrolysis rate and susceptibility of the materials toward hydrolysis. This approach was evaluated by blending PE18,18 with poly(*H*-phosphonate) (PHP) while retaining the overall HDPE-like properties.^284^ The PHP component was shown to completely hydrolyze during 4 months at 25 °C in phosphate buffer. Furthermore, clear degradation of PE18,18 as a blend component was observed, as shown by molar mass reduction after 4 months from 50 000–70 000 g mol^–1^ to 7000–11 000 g mol^–1^, depending on the blend composition. However, no significant further molar mass reduction was observed when the degradation period was expanded to 12 months. This could be explained by degradation of PHP during the first four months as well as increased crystallinity for PE18,18. The weight loss also remained low throughout the aging period of 12 months, which is connected to the still relatively high molar mass and low water solubility of oligomers and monomers. No signs of degradation or molar mass reduction was observed for plain PE18,18 material during the same time period under the same conditions.

### 7.2 Chemical Recyclability

Although PE is well suited for mechanical recycling, each use phase and reprocessing cycle will age the materials until it is no longer suitable for mechanical recycling, at least without significant downcycling. Significant efforts have been made both by industry and the scientific community to chemically recycle PE,^285,286^ however, the processes typically require high temperatures and/or long reaction times to break the C–C bonds. Chemical recycling of PE typically leads to mixtures of chemicals instead of the ethylene monomer, i.e., the attractive closed-loop enabling a truly circular polymer economy is not reached, at least not without further refining steps. In contrast, chemical recycling of condensation polymers back to original monomers is generally significantly easier due to the reversibility of the functional groups such as ester and amide bonds. These reactions typically require less energy and lead to original monomers or other well-defined compounds. One interesting approach could thus be chemical recycling of postconsumer PE materials (LDPE, HDPE) to compounds such as dicarboxylic acids or α,ω-divinyl functionalized oligomers that could be further oxidized to dicarboxylic acid or hydrated to alcohols for production of circular aliphatic polyesters, such as polyethylene-like polyesters.^287−289^ This way, traditional polyolefins could be turned to closed-loop recyclable materials designed for more easily managed end-of-life while retaining the polyethylene-like properties and processability.

Recent studies support the attractive chemical recyclability of polyethylene-like polyesters. PE18,18 synthesized from plant oil derived C18 diester (1,18-dimethyl ester of octadecanedioic acid) and C18 diol (1,18-octadecane diol) exhibited mechanical properties and thermal processability similar to HDPE.^273^ Chemical recycling by solvolysis in methanol at 150 °C led to 96% recovery of original monomers after 12 h without a catalyst. In comparison, the pyrolytic recycling of PE typically requires temperatures around 800 °C and leads to low (∼10%) recovery of ethene monomer. After evaporation of methanol, the obtained 1:1 mixture of C18 diol and C18 dimethyl ester were directly repolymerizable to high molar mass PE18,18. Similar process was utilized to chemically recycle biomass-derived polyethylene-like surface coatings for fabrics,^290^ while ester-linked polypropylene (PP), synthesized from telechelic polypropylene macromonomer, illustrated LLDPE-like thermal and mechanical properties.^291^ Heating the polymer in ethylene glycol in the presence of triazabicyclodecene (TBD) as transesterification catalyst at 190 °C for 24 h led to 93% conversion of ester groups to reform the 2-hydroxyethyl-terminated macromonomer. The recycled macromonomer could be repolymerized under the original polymerization conditions to a material closely resembling the original ester-linked PP. Furthermore, macrolactones (e.g., ω-pentadecalactone, globalide, 6-hexadecenlactone) derived polyethylene-like polyesters could be enzymatically recycled by utilizing immobilized *Candida antarctica* lipase B.^292^ The enzymatic recycling proceeded in toluene at 70 °C during 72 h and led to cyclic monomers and dimers as the main products. These macrolactones were proven to be repolymerizable.

In conclusion, polyethylene-like polyesters are attractive materials that can potentially be produced from biobased resources and circulated through mechanical and chemical recycling and biodegradation. However, biodegradability of these materials is not guaranteed and might require specific structural considerations, such as utilization of short diols in combination with long dicarboxylic acids. Biodegradation of polyethylene-like polyesters with systematic structural variations should be further investigated in different environments to more clearly define the limits of biodegradability and how it can be influenced by structural modifications.

These materials could replace PE, especially in applications where chemical recycling or biodegradation are targeted end-of-life options. However, while the chemical recycling of polyethylene-like polyesters is facile compared to conditions required for chemical recycling of PE, the production of PE is expected to require less energy. A comparative life-cycle assessment from cradle-to-cradle could further highlight the benefits of each material taking into account the whole life cycle.

## 8 Outlook

Polymer materials can in many ways contribute to the sustainable development goals. At the same time, the current production and especially how we use and dispose our materials poses a significant threat to our environment. The paradigm shift from linear to circular polymer economy is possible, but it requires overcoming several logistic, engineering, societal, and scientific challenges. This review focused on the latter one by highlighting different strategies to chemically design polymers, especially polyesters, for circularity. Polyesters have all the prerequisites to become ideal circular materials of the future, but further research is needed to optimize materials for different application-dependent end-of-life options. Here, chemistry offers almost endless possibilities, both to modify existing commercial polyesters or to design new structures from emerging biobased monomers, to tailor-make both the performance properties and end-of-life management. As an example, incorporation of groups or segments in the polyester chain that are more susceptible to hydrolysis or incorporation of functional groups that can internally catalyze bond exchange reaction, hydrolytic degradation, and chemical recycling are examples of promising chemical modifications. The rapid development in dynamic covalent chemistries opens up new avenues for modification of polyesters with other dynamic bonds to tailor the bond exchange reactions and susceptibility to external stimuli such as heat, pH, or UV light to provide circularity under mild conditions. Developments in green chemical and biological catalysts further promotes the circular processes and embedding such catalysts in the polyester matrix can help to ensure the degradation process during intended open environment degradation of products such as geotextiles, mulching films, or tree shelters. We have the necessary chemical toolbox, but ensuring high performance during service, in combination with ambient chemical recyclability or rapid biodegradability at the end-of-life is still a demanding and multidimensional optimization challenge requiring more research.

## References

[ref1] Sustainable Production and Consumption: Design for Disassembly As a Circular Economy Tool–Foresight Brief 031; United Nations Environment Programme, 2023; https://wedocs.unep.org/20.500.11822/43586 (accessed 2024-04-05).

[ref2] 2022 Annual Report; US Plastics Pact, 2022; https://usplasticspact.org/wp-content/uploads/2024/03/USPP_2022AnnualReport_to-share.pdf (accessed 2024-04-05).

[ref3] Circular Material Use Rate in Europe; European Environment Agency, 2024; https://www.eea.europa.eu/en/analysis/indicators/circular-material-use-rate-in-europe (accessed 2024-04-05).

[ref4] Payne J.; Jones M. D. The Chemical Recycling of Polyesters for a Circular Plastics Economy: Challenges and Emerging Opportunities. ChemSusChem 2021, 14, 4041–4070. 10.1002/cssc.202100400. 33826253 PMC8518041

[ref5] Haider T. P.; Völker C.; Kramm J.; Landfester K.; Wurm F. R. Plastics of the Future? The Impact of Biodegradable Polymers on the Environment and on Society. Angew. Chem., Int. Ed. 2019, 58, 50–62. 10.1002/anie.201805766. 29972726

[ref6] Lim J. Y. C.; Truong T. N. B.; Teo J. Y. Q.; Wang C. G.; Li Z. In Circularity of Plastics. Sustainability, Emerging Materials, and Valorization of Waste Plastic, Li Z.; Lim J. Y. C.; Eds.; Elsevier, 2023; pp 1–34.

[ref7] Vollmer I.; Jenks M. J. F.; Roelands M. C. P.; White R. J.; van Harmelen T.; de Wild P.; van der Laan G. P.; Meirer F.; Keurentjes J. T. F.; Weckhuysen B. M. Beyond Mechanical Recycling: Giving New Life to Plastic Waste. Angew. Chem., Int. Ed. 2020, 59, 15402–15423. 10.1002/anie.201915651. PMC7497176 32160372

[ref8] Lang W. T.; Mehta S. A.; Thomas M. M.; Openshaw D.; Westgate E.; Bagnato G. Chemical Recycling of Polyethylene Terephthalate, an Industrial and Sustainable Opportunity for Nortwest of England. J. Environ. Chem. Eng. 2023, 11, 110585 10.1016/j.jece.2023.110585.

[ref9] Tournier V.; Topham C. M.; Gilles A.; David B.; Folgoas C.; Moya-Leclair E.; Kamionka E.; Desrousseaux M.-L.; Texier H.; Gavalda S.; et al. An Engineered PET Depolymerase to Break Down and Recycle Plastic Bottles. Nature 2020, 580, 216–219. 10.1038/s41586-020-2149-4. 32269349

[ref10] Jia Z.; Gao L.; Qin L.; Yin J. Chemical Recycling of PET to Value-Added Products. RSC Sustain. 2023, 1, 2135–2147. 10.1039/D3SU00311F.

[ref11] Uekert T.; Singh A.; DesVeaux J. S.; Ghosh T.; Bhatt A.; Yadav G.; Afzal S.; Walzberg J.; Knauer K. M.; Nicholson S. R.; et al. Technical, Economic, and Environmental Comparison of Closed-Loop Recycling Technologies for Common Plastics. ACS Sustainable. Chem. Eng. 2023, 11, 965–978. 10.1021/acssuschemeng.2c05497.

[ref12] Plummer C. M.; Li L.; Chen Y. Ring-Opening Polymerization for the Goal of Chemically Recyclable Polymers. Macromolecules 2023, 56, 731–750. 10.1021/acs.macromol.2c01694. 36818576 PMC9933900

[ref13] Olsén P.; Undin J.; Odelius K.; Keul H.; Albertsson A.-C. Switching from Controlled Ring-Opening Polymerization (cROP) to Controlled Ring-Closing Depolymerization (cRCDP) by Adjusting the Reaction Parameters that Determine the Ceiling Temperature. Biomacromolecules 2016, 17, 3995–4002. 10.1021/acs.biomac.6b01375. 27783494 PMC5155308

[ref14] Hong M.; Chen E. Y.-X. Completely Recyclable Biopolymers with Linear and Cyclic Topologies via Ring-Opening Polymerization of γ-Butyrolactone. Nat. Chem. 2016, 8, 42–49. 10.1038/nchem.2391. 26673263

[ref15] MacDonald J. P.; Shaver M. P. An Aromatic/Aliphatic Polyester Prepared via Ring-Opening Polymerization and its Remarkably Selective and Cyclable Depolymerization to Monomer. Polym. Chem. 2016, 7, 553–559. 10.1039/C5PY01606A.

[ref16] Zhou L.; Zhang Z.; Shi C.; Scoti M.; Barange D. K.; Gowda R. R.; Chen E. Y.-X. Chemically Circular, Mechanically Tough, and Melt-Processable Polyhydroxyalkanoates. Science 2023, 380, 64–69. 10.1126/science.adg4520. 37023198

[ref17] Shi C.; Li Z. C.; Caporaso L.; Cavallo L.; Falivene L.; Chen E. Y.-X. Hybrid Monomer Design for Unifying Conflicting Polymerizability, Recyclability, and Performance Properties. Chem. 2021, 7, 670–685. 10.1016/j.chempr.2021.02.003.

[ref18] Cederholm L.; Olsén P.; Hakkarainen M.; Wohlert J.; Odelius K. ”Like Recycles Like”: Selective Ring-Closing Depolymerization of Poly(L-lactic acid) to L-Lactide. Angew. Chem., Int. Ed. 2022, 61, e20220453 10.1002/anie.202204531. PMC9541399 35582840

[ref19] Cederholm L.; Olsén P.; Hakkarainen M.; Odelius K. Design for Recycling: Polyester and Polycarbonate Based A–B–A Block Copolymers and Their Recyclability Back to Monomer. Macromolecules 2023, 56, 3641–3649. 10.1021/acs.macromol.3c00274.

[ref20] Dong B.; Xu G.; Yang R.; Guo X.; Wang Q. Preparation of Block Copolymers by a Sequential Transesterification Strategy: A Feasible Route for Upcycling End-of-Life Polyester Plastics to Elastomers. Macromolecules 2023, 56, 10143–10152. 10.1021/acs.macromol.3c01773.

[ref21] Hong M.; Chen E. Y.-X. Chemically Recyclable Polymers: A Circular Economy Approach to Sustainability. Green Chem. 2017, 19, 3692 10.1039/C7GC01496A.

[ref22] Coates G. W.; Getzler D. Y. L. Chemical Recycling to Monomer for an Ideal, Circular Polymer Economy. Nat. Rev. Mater. 2020, 5, 501–516. 10.1038/s41578-020-0190-4.

[ref23] Biodegradability of Plastics in the Open Environment Science Advice for Policy by European Academies (SAPEA); Scientific Advice Mechanism, 2020; https://scientificadvice.eu/advice/biodegradability-of-plastics-in-the-open-environment/ (accessed 2024-04-05).

[ref24] Bauchmüller V.; Carus M.; Chinthapalli R.; Dammer L.; Hark N.; Partanen A.; Ruiz P.; Lajewski S. BioSinn: Products for which Biodegradation Makes Sense Nova Institute; Nova Institute: Huerth, Germany, 2021; https://renewable-carbon.eu/publications/product/biosinn-products-for-which-biodegradation-makes-sense-pdf/ (accessed 2024-04-05).

[ref25] Law K. L.; Narayan R. Reducing Environmental Plastic Pollution by Designing Polymer Materials for Managed End-of-Life. Nat. Rev. Mater. 2022, 7, 104–116. 10.1038/s41578-021-00382-0.

[ref26] Bioplastics Market Development Update 2023; European Bioplastics eV: Berlin, 2024; https://www.european-bioplastics.org/bioplastics-market-development-update-2023-2/ (accessed 2024-04-05).

[ref27] Ghosh K.; Jones B. H. Roadmap to Biodegradable Plastics - Current State and Research Needs. ACS Sustainable. Chem. Eng. 2021, 9, 6170–6187. 10.1021/acssuschemeng.1c00801.

[ref28] Wang G.-X.; Huang D.; Ji J.-H.; Völker C.; Wurm F. R. Seawater-Degradable Polymers-Fighting the Marine Plastic Pollution. Adv. Sci. 2021, 8, 2001121 10.1002/advs.202001121. PMC7788598 33437568

[ref29] Chamas A.; Moon H.; Zheng J.; Qiu Y.; Tabassum T.; Jang J. H.; Abu-Omar M.; Scott S. L.; Suh S. Degradation Rates of Plastics in the Environment. ACS Sustainable. Chem. Eng. 2020, 8, 3494–3511. 10.1021/acssuschemeng.9b06635.

[ref30] Egan J.; Salmon S. Strategies and Progress in Synthetic Textile Fiber Biodegradability. SN. Appl. Sci. 2022, 4, 22 10.1007/s42452-021-04851-7.

[ref31] Hakkarainen M. Aliphatic Polyesters: Abiotic and Biotic Degradation and Degradation Products. Adv. Polym. Sci. 2002, 157, 113–138. 10.1007/3-540-45734-8_4.

[ref32] Narancic T.; Verstichel S.; Reddy Chaganti S.; Morales-Gamez L.; Kenny S. T.; De Wilde B.; Babu Padamati R.; O'Connor K. E. Biodegradable Plastic Blends Create New Possibilities for End-of-Life Management of Plastics but They Are Not a Panacea for Plastic Pollution. Environ. Sci. Technol. 2018, 52, 10441–10452. 10.1021/acs.est.8b02963. 30156110

[ref33] Mochizuki M.; Hirami M. Structural Effects on the Biodegradation of Aliphatic Polyesters. Polym. Adv. Technol. 1997, 8, 203–209. 10.1002/(SICI)1099-1581(199704)8:4<203::AID-PAT627>3.0.CO;2-3.

[ref34] Kim M. S.; Chang H.; Zheng L.; Yan Q.; Pfleger B. F.; Klier J.; Nelson K.; Majumder E. L.-W.; Huber G. W. A Review of Biodegradable Plastics: Chemistry, Applications, Properties, and Future Research Needs. Chem. Rev. 2023, 123, 9915–9939. 10.1021/acs.chemrev.2c00876. 37470246

[ref35] Larramaga A.; Lizundia E. A Review on the Thermomechanical Properties and Biodegradation Behavior of Polyesters. Eur. Polym. J. 2019, 121, 109296 10.1016/j.eurpolymj.2019.109296.

[ref36] Albertsson A.-C.; Hakkarainen M. Designed to Degrade-Suitably Designed Degradable Polymers Can Play a Role in Reducing Plastic Waste. Science 2017, 358, 872–873. 10.1126/science.aap8115. 29146799

[ref37] Zumstein M. T.; Narayan R.; Kohler H.-P. E.; McNeill K.; Sander M. Dos and Do Nots When Assessing the Biodegradation of Plastics. Environ. Sci. Technol. 2019, 53, 9967–9969. 10.1021/acs.est.9b04513. 31418543

[ref38] Doi Y.; Abe H. Structural Effects on Biodegradation of Aliphatic Polyesters. Macromol. Symp. 1997, 118, 725–731. 10.1002/masy.19971180193.

[ref39] Biodegradable Polymers in Various Environments According to Established Standards and Certification Schemes Nova-Institute.; Nova-Institute: Huerth, 2021; https://renewable-carbon.eu/publications/product/biodegradable-polymers-in-various-environments-according-to-established-standards-and-certification-schemes-graphic-png/ (accessed 2024-04-05).

[ref40] Höglund A.; Odelius K.; Albertsson A.-C. Crucial Differences in the Hydrolytic Degradation between Industrial Polylactide and Laboratory-Scale Poly(L-lactide). ACS Appl. Mater. Interfaces 2012, 4, 2788–2793. 10.1021/am300438k. 22563747 PMC3359772

[ref41] Hakkarainen M.; Höglund A.; Odelius K.; Albertsson A.-C. Tuning the Release Rate of Acidic Degradation Products through Macromolecular Design of Caprolactone-Based Copolymers. J. Am. Chem. Soc. 2007, 129, 6308–6312. 10.1021/ja0702871. 17439125

[ref42] Bonartsev A. P.; Boskhomodgiev A. P.; Iordanskii A. L.; et al. Hydrolytic Degradation of Poly(3-hydroxybutyrate), Polylactide and their Derivatives: Kinetics, Crystallinity, and Surface Morphology. Mol. Cryst. Liq. Cryst. 2012, 556, 288–300. 10.1080/15421406.2012.635982.

[ref43] Lyyra I.; Sandberg N.; Parihar V. S.; Hannula M.; Huhtala H.; Hyttinen J.; Massera J.; Kellomäki M. Hydrolytic Degradation of Polylactide/Polybutylene Succinate Blends with Bioactive Glass. Mater. Today Commun. 2023, 37, 107242 10.1016/j.mtcomm.2023.107242.

[ref44] Hu H.; Tian Y.; Wang J.; Zhang R.; Zhu J. Enhanced Degradation and Gas Barrier of PBAT through Composition Design of Aliphatic Units. Polym. Degrad. Stab. 2022, 195, 109795 10.1016/j.polymdegradstab.2021.109795.

[ref45] Tsuji H.; Suzuyoshi K. Environmental Degradation of Biodegradable Polyesters 1. Poly(ε-caprolactone), Poly[(R)-3-hydroxybutyrate], and Poly(L-lactide) Films in Controlled Static Seawater. Polym. Degrad. Stab. 2002, 75, 347–355. 10.1016/S0141-3910(01)00240-3.

[ref46] Kasuya K.; Takagi K.; Ishiwatari S.; Yoshida Y.; Doi Y. Biodegradabilities of Various Aliphatic Polyesters in Natural Waters. Polym. Degrad. Stab. 1998, 59 (1–3), 327–332. 10.1016/S0141-3910(97)00155-9.

[ref47] Palsikowski P. A.; Kuchnier C. N.; Pinheiro I. F.; Morales A. R. Biodegradation in Soil of PLA/PBAT Blends Compatibilized with Chain Extender. J. Polym. Environ. 2018, 26, 330–341. 10.1007/s10924-017-0951-3.

[ref48] Mariani P. D. S. C.; Vinagre Neto A. P.; da Silva J. P. Jr.; Cardoso E. J. B. N.; Esposito E.; Innocentini-Mei L. H. Mineralization of Poly(ε-caprolactone)/Adipate Modified Starch Blend in Agricultural Soil. J. Polym. Environ. 2007, 15, 19–24. 10.1007/s10924-006-0044-1.

[ref49] Nelson T. F.; Baumgartner R.; Jaggi M.; Bernasconi S. M.; Battagliarin G.; Sinkel C.; Künkel A.; Kohler H.-P. E.; McNeill K.; Sander M. Biodegradation of Poly(butylene succinate) in Soil Laboratory Incubations Assessed by Stable Carbon Isotope Labelling. Nat. Commun. 2022, 13, 5691 10.1038/s41467-022-33064-8. 36171185 PMC9519748

[ref50] Briassoulis D.; Mistriotis A.; Mortier N.; Tosin M. A Horizontal Test Method for Biodegradation in Soil of Bio-Based and Conventional Plastics and Lubricants. J. Cleaner Prod. 2020, 242, 118392 10.1016/j.jclepro.2019.118392.

[ref51] Kalita N. K.; Bhasney S. M.; Mudenur C.; Kalamdhad A.; Katiyar V. End-of-Life Evaluation and Biodegradation of Poly(lactic acid) (PLA)/Polycaprolactone (PCL)/Microcrystalline Cellulose (MCC) Polyblends under Composting Conditions. Chemosphere 2020, 247, 125875 10.1016/j.chemosphere.2020.125875. 32069712

[ref52] Weng Y.-X.; Wang Y.; Wang X.-L.; Wang Y.-Z. Biodegradation behavior of PHBV Films in a Pilot-Scale Composting Conditions. Polym. Test. 2010, 29, 579–587. 10.1016/j.polymertesting.2010.04.002.

[ref53] Intaraksa P.; Rudeekit Y.; Siriyota P.; Chaiwutthinan P.; Tajan M.; Leejarkpai T. The Ultimate Biodegradation of the Starch Based Biodegradable Plastics. Adv. Mater. Res. 2012, 506, 327–330. 10.4028/www.scientific.net/AMR.506.327.

[ref54] Xu J.; Feng K.; Li Y.; Xie J.; Wang Y.; Zhang Z.; Hu Q. Enhanced Biodegradation Rate of Poly(butylene adipate-co-terephthalate) Composites Using Reed Fiber. Polymers 2024, 16, 411 10.3390/polym16030411. 38337300 PMC10857307

[ref55] Sander M.; Weber M.; Lott C.; Zumstein M.; Künkel A.; Battagliarin G. Polymer Biodegradability 2.0: A Holistic View on Polymer Biodegradation in Natural and Engineered Environments. Adv. Polym. Sci. 2023, 293, 65–110. 10.1007/12_2023_163.

[ref56] Sekiguchi T.; Saika A.; Nomura K.; Watanabe T.; Watanabe T.; Fujimoto Y.; Enoki M.; Sato T.; Kato C.; Kanehiro H. Biodegradation of Aliphatic Polyesters Soaked in Deep Seawaters and Isolation of Poly(ε-caprolactone)-Degrading Bacteria. Polym. Degrad. Stab. 2011, 96, 1397–1403. 10.1016/j.polymdegradstab.2011.03.004.

[ref57] Nakayama A.; Yamano N.; Kawasaki N. Biodegradation in Seawater of Aliphatic Polyesters. Polym. Degrad. Stab. 2019, 166, 290–299. 10.1016/j.polymdegradstab.2019.06.006.

[ref58] Woodard L. N.; Grunlan M. A. Hydrolytic Degradation and Erosion of Polyester Biomaterials. ACS Macro Lett. 2018, 7, 976–982. 10.1021/acsmacrolett.8b00424. 30705783 PMC6350899

[ref59] Abe H.; Doi Y. Structural Effects on Enzymatic Degradabilities for Poly[(R)-3-hydroxybutyric acid] and Its Copolymers. Int. J. Biol. Macromol. 1999, 25, 185–192. 10.1016/S0141-8130(99)00033-1. 10416666

[ref60] Erdal N. B.; Albara Lando G.; Yadav A.; Srivastava R. K.; Hakkarainen M. Hydrolytic Degradation of Porous Crosslinked Poly(ε-caprolactone) Synthesized by High Internal Phase Emulsion Templating. Polymers 2020, 12, 1849 10.3390/polym12081849. 32824691 PMC7464575

[ref61] Höglund A.; Odelius K.; Hakkarainen M.; Albertsson A.-C. Controllable degradation product migration from cross-linked biomedical polyester-ethers through predetermined alterations in copolymer composition. Biomacromolecules 2007, 8, 2025–2032. 10.1021/bm070292x. 17521165

[ref62] Agarwal S.; Speyerer C. Degradable Blends of Semi-Crystalline and Amorphous Branched Poly(caprolactone): Effect of Microstructure on Blend Properties. Polymer 2010, 51, 1024–1032. 10.1016/j.polymer.2010.01.020.

[ref63] Rychter P.; Biczak R.; Herman B.; Smylla A.; Kurcok P.; Adamus G.; Kowalczuk M. Environmental Degradation of Polyester Blends Containing Atactic Poly(3-hydroxybutyrate). Biodegradation in Soil and Ecotoxicological Impact. Biomacromolecules 2006, 7, 3125–3131. 10.1021/bm060708r. 17096541

[ref64] Andersson S. R.; Hakkarainen M.; Albertsson A.-C. Tuning the Polylactide Hydrolysis Rate by Plasticizer Architecture and Hydrophilicity without Introducing New Migrants. Biomacromolecules 2010, 11, 3617–3623. 10.1021/bm101075p. 21080699

[ref65] Höglund A.; Hakkarainen M.; Edlund U.; Albertsson A.-C. Surface Modification Changes the Degradation Process and Degradation Product Pattern of Polylactide. Langmuir 2010, 26, 378–383. 10.1021/la902166j. 20038176

[ref66] Li S. M. Hydrolytic Degradation Characteristics of Aliphatic Polyesters Derived from Lactic and Glycolic Acids. J. Biomed. Mater. Res. 1999, 48, 342–353. 10.1002/(SICI)1097-4636(1999)48:3<342::AID-JBM20>3.0.CO;2-7. 10398040

[ref67] Li S. M.; Garreau H.; Vert M. Structure-Property Relationships in the Case of Massive Aliphatic Poly(α-hydroxyacids) in Aqueous Media. Part 2: Degradation of Lactide/Glycolide Copolymers: PLA37.5GA25 and PLA75GC25. J. Mater. Sci.: Mater. Med. 1990, 1, 131–139. 10.1007/BF00700872.

[ref68] Rahaman Md. H.; Tsuji H. Hydrolytic Degradation Behavior of Stereo Multiblock and Diblock Poly(lactic acid)s: Effects of Block Lengths. Polym. Degrad. Stab. 2013, 98, 709–719. 10.1016/j.polymdegradstab.2012.12.025.

[ref69] Tsuji H. Poly(lactide) Stereocomplexes: Formation, Structure, Properties, Degradation, and Applications. Macromol. Biosci. 2005, 5, 569–597. 10.1002/mabi.200500062. 15997437

[ref70] Andersson S. R.; Hakkarainen M.; Inkinen S.; Södergård A.; Albertsson A.-C. Polylactide Stereocomplexation Leads to Higher Hydrolytic Stability but More Acidic Hydrolysis Product Pattern. Biomacromolecules 2010, 11, 1067–1073. 10.1021/bm100029t. 20201493

[ref71] Tsuji H.; Tsuruno T. Water Vapor Permeability of Poly(L-lactide)/Poly(D-lactide) Stereocomplexes. Macromol. Mater. Eng. 2010, 295, 709–715. 10.1002/mame.201000071.

[ref72] Hakkarainen M.; Adamus G.; Höglund A.; Kowalczuk M.; Albertsson A.-C. ESI-MS Reveals the Influence of Hydrophilicity and Architecture on the Water-Soluble Degradation Product Patterns of Biodegradable Homo- and Copolyesters of 1,5-Dioxepan-2-one and ε-Caprolactone. Macromolecules 2008, 41, 3547–3554. 10.1021/ma800365m.

[ref73] Chen X.; Wu X.; Fan Z.; Zhao Q.; Liu Q. Biodegradable Poly(trimethylene carbonate-*b*-(L-lactide-*ran*-glycolide)) Terpolymers with Tailored Molecular Structure and Advanced Performance. Polym. Adv. Technol. 2018, 29, 1684–1696. 10.1002/pat.4272.

[ref74] Lefèvre C.; Mathieu C.; Tidjani A.; Dupret I.; Vander Wauven C.; De Winter W.; David C. Comparative Degradation by Microorganisms of Terephthalic Acid, 2,6-Naphthalene Dicarboxylic Acid, Their Esters and Polyesters. Polym. Degrad. Stab. 1999, 64, 9–16. 10.1016/S0141-3910(98)00164-5.

[ref75] Mahata D.; Karthikeyan S.; Godse R.; Gupta V. K. Poly(butylene adipate-co-terephthalate) Polyester Synthesis Process and Product Development. Polym. Sci. Ser. C 2021, 63, 102–111. 10.1134/S1811238221010045.

[ref76] Witt U.; Yamamoto M.; Seeliger U.; Müller R.-J.; Warzelhan V. Biodegradable Polymeric Materials – Not the Origin but the Chemical Structure Determines Biodegradability. Angew. Chem., Int. Ed. 1999, 38, 1438–1442. 10.1002/(SICI)1521-3773(19990517)38:10<1438::AID-ANIE1438>3.0.CO;2-U. 29711570

[ref77] Witt U.; Müller R.-J.; Deckwer W.-D. Biodegradation Behavior and Material Properties of Aliphatic/Aromatic Polyesters of Commercial Importance. J. Environ. Polym. Degrad. 1997, 5, 81–89. 10.1007/BF02763591.

[ref78] Witt U.; Einig T.; Yamamoto M.; Kleeberg L.; Deckwer W.-D.; Müller R.-J. Biodegradation of Aliphatic-Aromatic Copolyesters: Evaluation of the Final Biodegradability and Ecotoxicological Impacts of Degradation Intermediates. Chemosphere 2001, 44, 289–299. 10.1016/S0045-6535(00)00162-4. 11444312

[ref79] Zumstein M. T.; Rechsteiner D.; Roduner N.; Perz V.; Ribitsch D.; Guebitz G. M.; Kohler H.-P. E.; McNeill K.; Sander M. Enzymatic Hydrolysis of Polyester Thin Films at the Nanoscale: Effects of Polyester Structure and Enzyme Active-Site Accessibility. Env. Sci. Eng. 2017, 51, 7476–7485. 10.1021/acs.est.7b01330. 28538100

[ref80] Jiang B.; Wang Y.; Peng Z.; Lim K. H.; Wang Q.; Shi S.; Zheng J.; Yang X.; Liu P.; Wang W.-J. Synthesis of Poly(butylene adipate-co-terephthalate-co-poly(glycolic acid) with Enhanced Degradability in Water. Macromolecules 2023, 56, 9207–9217. 10.1021/acs.macromol.3c01403.

[ref81] Stegmann P.; Gerritse T.; Shen L.; Londo M.; Puente Ã.; Junginger M. The Global Warming Potential and the Material Utility of PET and Bio-Based PEF Bottles over Multiple Recycling Trips. J. Clean. Prod. 2023, 395, 136426 10.1016/j.jclepro.2023.136426.

[ref82] Fei X.; Wang J.; Zhu J.; Wang X.; Liu X. Biobased Poly(ethylene 2,5-furancoate): No Longer an Alternative, but an Irreplaceable Polyester in the Polymer Industry. ACS Sustainable. Chem. Eng. 2020, 8, 8471–8485. 10.1021/acssuschemeng.0c01862.

[ref83] De Jong E.; Visser H. A.; Sousa Dias A.; Harvey C.; Gruter G.-J. M. The Road to Bring FDCA and PEF to the Market. Polymers 2022, 14, 943 10.3390/polym14050943. 35267764 PMC8912366

[ref84] Papadopoulos L.; Magaziotis A.; Nerantzaki M.; Terzopoulou Z.; Papageorgiou G. Z.; Bikiaris D. N. Synthesis and Characterization of Novel Poly(ethylene furanoate-co-adipate) Random Copolyesters with Enhanced Biodegradability. Polym. Degrad. Stab. 2018, 156, 32–42. 10.1016/j.polymdegradstab.2018.08.002.

[ref85] Terzopoulou Z.; Tsanaktsis V.; Bikiaris D. N.; Exarhopoulos S.; Papageorgiou D. G.; Papageorgiou G. Z. Biobased Poly(ethylene furanoate-co-ethylene succinate) Copolyesters: Solid State Structure, Melting Point Depression and Biodegradability. RSC Adv. 2016, 6, 84003–84015. 10.1039/C6RA15994J.

[ref86] Kim H. J.; Reddi Y.; Cramer C. J.; Hillmyer M. A.; Ellison C. J. Readily Degradable Aromatic Polyesters from Salicylic Acid. ACS Macro Lett. 2020, 9, 96–102. 10.1021/acsmacrolett.9b00890. 35638662

[ref87] Li M.-Q.; Luo Z.-X.; Yu X.-Y.; Tian G.-Q.; Wu G.; Chen S.-C.; Wang Y.-Z. Ring-Opening Polymerization of a Seven-Membered Lactone Toward a Biocompatible, Degradable, and Recyclable Semi-Aromatic Polyester. Macromolecules 2023, 56, 2465–2475. 10.1021/acs.macromol.2c02172.

[ref88] Kim H. J.; Hillmyer M. A.; Ellison C. J. Enhanced Polyester Degradation through Transesterification with Salicylates. J. Am. Chem. Soc. 2021, 143, 15784–15790. 10.1021/jacs.1c07229. 34529416

[ref89] Kolahchi A. R.; Ajji A.; Carreau P. J. Enhancing Hydrophilicity of Polyethylene Terephthalate Surface through Melt Blending. Polym. Eng. Sci. 2015, 55, 349–358. 10.1002/pen.23910.

[ref90] Hong M.; Chen J.; Chen E. Y.-X. Polymerization of Polar Monomers Mediated by Main-Group Lewis Acid-Base Pairs. Chem. Rev. 2018, 118, 10551–10616. 10.1021/acs.chemrev.8b00352. 30350583

[ref91] Wanasinghe S. V.; Dodo O. J.; Konkolewicz D. Dynamic Bonds: Adaptable Time Scales for Responsive Materials. Angew. Chem., Int. Ed. 2022, 61, e2022069 10.1002/anie.202206938. PMC10092857 36167937

[ref92] Hakkarainen M.; Karlsson S.; Albertsson A.-C. Influence of Low Molecular Weight Lactic Acid Derivatives on Degradability of Polylactide. J. Appl. Polym. Sci. 2000, 76, 228–239. 10.1002/(SICI)1097-4628(20000411)76:2<228::AID-APP12>3.0.CO;2-B.

[ref93] Li S. M.; Garreau H.; Vert M. Structure-Property Relationships in the Case of the Degradation of Massive Aliphatic Poly-(α-hydroxyacids) in Aqueous Media. Part 1: Poly(DL-lactic acid). J. Mater. Sci.: Mater. Med. 1990, 1, 123–130. 10.1007/BF00700871.

[ref94] De Jong S. J.; Arias E. R.; Rijkers D. T. S.; van Nostrum C. F.; Kettenes-van den Bosch J. J.; Hennink W. E. New Insights into the Hydrolytic Degradation of Poly(lactic acid): Participation of the Alcohol Terminus. Polymer 2001, 42, 2795–2802. 10.1016/S0032-3861(00)00646-7.

[ref95] Benedict C. V.; Cook W. J.; Jarrett P.; Cameron J. A.; Huang S. J.; Bell J. P. Fungal Degradation of Polycaprolactones. J. Appl. Polym. Sci. 1983, 28, 327–334. 10.1002/app.1983.070280128.

[ref96] van Nostrum C. F.; Veldhuis T. F. J.; Bos G. W.; Hennink W. E. Hydrolytic Degradation of Oligo(lactic acid): A Kinetic and Mechanistic Study. Polymer 2004, 45, 6779–6787. 10.1016/j.polymer.2004.08.001.

[ref97] Therin M.; Christel P.; Li S. M.; Garreau H.; Vert M. *In Vivo* Degradation of Massive Poly(α-hydroxyacids): Validation of *In Vitro* Findings. Biomaterials 1992, 13, 594–600. 10.1016/0142-9612(92)90027-L. 1391406

[ref98] Van Lijsebetten F.; Holloway J. O.; Winne J. M.; Du Prez F. E. Internal Catalysis for Dynamic Covalent Chemistry Applications and Polymer Science. Chem. Soc. Rev. 2020, 49, 8425 10.1039/D0CS00452A. 33112323

[ref99] Qiu J.; Ma S.; Wang S.; Tang Z.; Li Q.; Tian A.; Xu X.; Wang B.; Lu N.; Zhu J. Upcycling of Polyethylene Terephthalate to Continuously Reprocessable Vitrimers through Reactive Extrusion. Macromolecules 2021, 54, 703–712. 10.1021/acs.macromol.0c02359.

[ref100] Altuna F. I.; Hoppe C. E.; Williams R. J. J. Epoxy Vitrimers with Covalently Bonded Tertiary Amine as Catalyst of the Transesterification Reaction. Eur. Polym. J. 2019, 113, 297–304. 10.1016/j.eurpolymj.2019.01.045.

[ref101] Hayashi M.; Inaba T. Achievement of a Highly Rapid Bond Exchange for Self-Catalyzed Polyester Vitrimers by Incorporating Tertiary Amino Groups on the Network Strands. ACS Appl. Polym. Mater. 2021, 3, 4424–4429. 10.1021/acsapm.1c00724.

[ref102] Delahaye M.; Tanini F.; Holloway J. O.; Winne J. M.; Du Prez F. E. Double Neighboring Group Participation for Ultrafast Exchange in Phthalate Monoester Networks. Polym. Chem. 2020, 11, 5207–5215. 10.1039/D0PY00681E.

[ref103] Hao C.; Liu T.; Zhang S.; Liu W.; Shan Y.; Zhang J. Triethanolamine-Mediated Covalent Adaptable Epoxy Network: Excellent Mechanical Properties, Fast Repairing, and Easy Recycling. Macromolecules 2020, 53, 3110–3118. 10.1021/acs.macromol.9b02243.

[ref104] Werber M. M.; Shalitin Y. The Reaction of Tertiary Amino Alcohols with Active Esters I. Acylation and Deacylation Steps. Bioorg. Chem. 1973, 2, 202–220. 10.1016/0045-2068(73)90024-2.

[ref105] Valverde C.; Lligadas G.; Ronda J. C.; Galià M.; Cádiz V. Hydrolytic and Enzymatic Degradation Studies of Aliphatic 10-Undecenioc Acid-Based Polyesters. Polym. Degrad. Stab. 2018, 155, 84–94. 10.1016/j.polymdegradstab.2018.07.012.

[ref106] Yang Z.; Li D.; Song C.; Shao P.; Wang S.; Wang Z.; Lv Y.; Wei Z. Synthesis of High Molecular Weight Hydroxy Functional Copolymers by Green and Selective Polycondensation Methods. RSC Adv. 2020, 10, 6414–6422. 10.1039/D0RA00120A. 35495984 PMC9049703

[ref107] Bender M. L.; Chloupek F.; Neveu M. C. Intramolecular Catalysis of Hydrolytic Reactions. III. Intramolecular Catalysis by Carboxylate Ion in the Hydrolysis of Methyl Hydrogen Phthalate. J. Am. Chem. Soc. 1958, 80, 5384–5387. 10.1021/ja01553a016.

[ref108] Thanassi J. W.; Bruice T. C. Neighboring Carboxylic Group Participation in the Hydrolysis of Monoesters of Phthalic Acid. The Dependence of Mechanisms on Leaving Group Tendencies. J. Am. Chem. Soc. 1966, 88, 747–752. 10.1021/ja00956a026. 5902559

[ref109] Delahaye M.; Winne J. M.; Du Prez F. E. Internal Catalysis in Covalent Adaptable Networks: Phthalate Monoester Transesterification as a Versatile Dynamic Cross-Linking Chemistry. J. Am. Soc. 2019, 141, 15277–15287. 10.1021/jacs.9b07269. 31469270

[ref110] Zhang H.; Majumdar S.; van Benthem R. A.T. M.; Sijbesma R. P.; Heuts J. P. A. Intramolecularly Catalyzed Dynamic Polyester Networks Using Neighboring Carboxylic and Sulfonic Acid Groups. ACS Macro Lett. 2020, 9, 272–277. 10.1021/acsmacrolett.9b01023. 35638690

[ref111] Fahnhorst G. W.; Hoye T. R. A Carbomethoxylated Polyvalerolactone from Malic Acid: Synthesis and Divergent Chemical Recycling. ACS Macro Lett. 2018, 7, 143–147. 10.1021/acsmacrolett.7b00889. 35610909 PMC12277036

[ref112] Fahnhorst G. W.; De Hoe G. X.; HIllmyer M. A.; Hoye T. R. 4-Carboalkoxylated Polyvalerolactones from Malic Acid: Tough and Degradable Polyesters. Macromolecules 2020, 53, 3194–3201. 10.1021/acs.macromol.0c00212. 34334815 PMC8323767

[ref113] Han S.-I.; Yoo Y.; Kim D. K.; Im S. S. Biodegradable Aliphatic Polyester Ionomers. Macromol. Biosci. 2004, 4, 199–207. 10.1002/mabi.200300095. 15468209

[ref114] Saumer A.; Mecking S. Recyclable and Degradable Ionic-Substituted Long-Chain Polyesters. ACS Sustainable. Chem. Eng. 2023, 11, 12414–12422. 10.1021/acssuschemeng.3c03141. 37621695 PMC10445281

[ref115] Chen X.; Dam M. A.; Ono K.; Mal A.; Shen H.; Nutt S. R.; Sheran K.; Wudl F. Thermally Re-Mendable Crosslinked Polymeric Material. Science 2002, 295, 1698–1702. 10.1126/science.1065879. 11872836

[ref116] Kloxin C. J.; Scott T. F.; Adzima B. J.; Bowman C. N. Covalent Adaptable Networks (CANs): A Unique Paradigm in Cross-Linked Polymers. Macromolecules 2010, 43, 2643–2653. 10.1021/ma902596s. 20305795 PMC2841325

[ref117] Montarnal D.; Capelot M.; Tournilhac F.; Leibler L. Silica-Like Malleable Materials from Permanent Organic Networks. Science 2011, 334, 965–968. 10.1126/science.1212648. 22096195

[ref118] Denissen W.; Winne J. M.; Du Prez F. E. Vitrimers: Permanent Organic Networks with Glass-Like Fluidity. Chem. Sci. 2016, 7, 30–38. 10.1039/C5SC02223A. 28757995 PMC5508697

[ref119] Kloxin C. J.; Bowman C. N. Covalent Adaptable Networks: Smart, Reconfigurable and Responsive Network Systems. Chem. Soc. Rev. 2013, 42, 7161–7173. 10.1039/C3CS60046G. 23579959

[ref120] Hammer L.; van Zee N. J.; Nicolaÿ R. Dually Crosslinked Polymer Networks Incorporating Dynamic Covalent Bonds. Polymers 2021, 13, 396 10.3390/polym13030396. 33513741 PMC7865237

[ref121] Yang R.; Xu G.; Dong B.; Hou H.; Wang Q. A “Polymer to Polymer” Chemical Recycling of PLA Plastics by the “DE-RE Polymerization. Strategy. Macromolecules 2022, 55, 1726–1735. 10.1021/acs.macromol.1c02085.

[ref122] Liguori A.; Hakkarainen M. Designed from Biobased Materials for Recycling: Imine-Based Covalent Adaptable Networks. Macromol. Rapid Commun. 2022, 43, 2100816 10.1002/marc.202100816. 35080074

[ref123] Huang S.; Kong X.; Xiong Y.; Zhang X.; Chen H.; Jiang W.; Niu Y.; Xu W.; Ren C. An Overview of Dynamic Covalent Bonds in Polymer Material and Their Applications. Eur. Polym. J. 2020, 141, 110094 10.1016/j.eurpolymj.2020.110094.

[ref124] Zheng N.; Xu Y.; Zhao Q.; Xie T. Dynamic Covalent Polymer Networks: A Molecular Platform for Designing Functions Beyond Chemical Recycling and Self-Healing. Chem. Rev. 2021, 121, 1716–1745. 10.1021/acs.chemrev.0c00938. 33393759

[ref125] Gong X.; Cheng Z.; Gao S.; Zhang D.; Ma Y.; Wang J.; Wang C.; Chu F. Ethyl Cellulose Based Self-Healing Adhesives Synthesized via RAFT and Aromatic Schiff-Base Chemistry. Carbohydr. Polym. 2020, 250, 116846 10.1016/j.carbpol.2020.116846. 33049809

[ref126] Gao S.; Cheng Z.; Zhou X.; Liu Y.; Wang J.; Wang C.; Chu F.; Xu F.; Zhang D. Fabrication of Lignin Based Renewable Dynamic Networks and Its Applications as Self-Healing, Antifungal and Conductive Additives. Chem. Eng. J. 2020, 394, 124896 10.1016/j.cej.2020.124896.

[ref127] Temizkan K.; Kaya I. Heat Resisting and Water-Soluble Chocolate Polyesters Containing Azomethine Group. Mater. Sci.-Polym. 2017, 35, 303–312. 10.1515/msp-2017-0018.

[ref128] Senol D.; Kaya I. Synthesis and characterization of azomethine polymers containing ether and ester. J. Saudi Chem. Soc. 2017, 21, 505–516. 10.1016/j.jscs.2015.05.006.

[ref129] Ravikumar L.; Prasad M. B.; Vasanthi B. J.; Gopalakrishnan K.; Rajeshkumar J.; Sengodan V. Synthesis, Characterization and Electrical Conductivity of New Poly(azomethine ester)s from Hydroxyacids. Mater. Chem. Phys. 2009, 115, 632–636. 10.1016/j.matchemphys.2009.01.030.

[ref130] da Cruz M. G. A.; Gueret R.; Chen J.; Piatek J.; Beele B.; Sipponen M. H.; Frauscher M.; Budnyk S.; Rodrigues B. V. M.; Slabon A. Electrochemical Depolymerization of Lignin in a Biomass-Based Solvent. ChemSusChem 2022, 15, e2022007 10.1002/cssc.202200718. 35856736

[ref131] Climent M. J.; Corma A.; Iborra S. Conversion of Biomass Platform Molecules into Fuel Additives and Liquid Hydrocarbon Fuels. Green Chem. 2014, 16, 516–547. 10.1039/c3gc41492b.

[ref132] Huang K.; Ma S.; Wang S.; Li Q.; Wu Z.; Liu J.; Liu R.; Zhu J. Sustainable Valorization of Lignin with Levulinic Acid and Its Application in Polyimine Thermosets. Green Chem. 2019, 21, 4964–4970. 10.1039/C9GC02384D.

[ref133] Liu Y.; Yu Z.; Lu G.; Chen W.; Ye Z.; He Y.; Tang Z.; Zhu J. Versatile Levulinic Acid-Derived Dynamic Covalent Thermosets Enabled by In Situ Generated Imine and Multiple Hydrogen Bonds. Chem. Eng. J. 2023, 451, 139053 10.1016/j.cej.2022.139053.

[ref134] Skene W. G.; Lehn J.-M. P. Dynamers: Polyacylhydrazone Reversible Covalent Polymers, Component Exchange, and Constitutional Diversity. Proc. Natl. Acad. Sci. U.S.A. 2004, 101, 8270–8275. 10.1073/pnas.0401885101. 15150411 PMC420383

[ref135] Yu Z.; Ma S.; Tang Z.; Liu Y.; Xu X.; Li Q.; Zhang K.; Wang B.; Wang S.; Zhu J. Amino Acids as Latent Curing Agents and Their Application in Fully Bio-Based Epoxy Resins. Green Chem. 2021, 23, 6566–6575. 10.1039/D1GC02126E.

[ref136] Li Y.; Liu T.; Zhang S.; Shao L.; Fei M.; Yu H.; Zhang J. Catalyst-Free Vitrimer Elastomer Based on Dimer Acid: Robust Mechanical Performance, Adaptivity and Hydrothermal Recyclability. Green Chem. 2020, 22, 870–881. 10.1039/C9GC04080C.

[ref137] Xu Y.; Odelius K.; Hakkarainen M. Photocurable, Thermally Reprocessable, and Chemically Recyclable Vanillin-Based Imine Thermosets. ACS Sustainable Chem. Eng. 2020, 8, 17272–1727. 10.1021/acssuschemeng.0c06248.

[ref138] Cortés-Guzmán K. P.; Parikh A. R.; Sparacin M. L.; Remy A. K.; Adegoke L.; Chitrakar C.; Ecker M.; Voit W. E.; Smaldone R. A. Recyclable, Biobased Photoresins for 3D Printing through Dynamic Imine Exchange. ACS Sustainable Chem. Eng. 2022, 10, 13091–13099. 10.1021/acssuschemeng.2c03541.

[ref139] Liguori A.; Subramaniyan S.; Yao J. G.; Hakkarainen M. Photocurable Extended Vanillin-Based Resin for Mechanically and Chemically Recyclable, Self-Healable and Digital Light Processing 3D Printable Thermosets. Eur. Polym. J. 2022, 178, 111489 10.1016/j.eurpolymj.2022.111489.

[ref140] Liguori A.; Garfias Gonzalez K. I.; Hakkarainen M. Unexpected Self-Assembly of Carbon Dots during Digital Light Processing 3D Printing of Vanillin Schiff-Base Resin. Polymer 2023, 283, 126252 10.1016/j.polymer.2023.126252.

[ref141] Snyder R. L.; Lidston C. A. L.; De Hoe G. X.; Parvulescu M. J. S.; Hillmyer M. A.; Coates G. W. Mechanically Robust and Reprocessable Imine Exchange Networks from Modular Polyester Pre-Polymers. Polym. Chem. 2020, 11, 5346 10.1039/C9PY01957J.

[ref142] Liguori A.; Oliva E.; Sangermano M.; Hakkarainen M. Digital Light Processing 3D Printing of Isosorbide-and Vanillin-Based Ester and Ester-Imine Thermosets: Structure-Property-Recyclability Relationships. ACS Sustainable Chem. Eng. 2023, 11, 14601–14613. 10.1021/acssuschemeng.3c04362. 37799818 PMC10548585

[ref143] Fukuda K.; Shimoda M.; Sukegawa M.; Nobori T.; Lehn J.-M. Doubly Degradable Dynamers: Dynamic Covalent Polymers Based on Reversible Imine Connections and Biodegradable Polyester Units. Green Chem. 2012, 14, 2907–2911. 10.1039/c2gc35875a.

[ref144] Collins J.; Xiao Z.; Connal L. A. Tunable Degradation of Polyethylene Glycol-Like Polymers Based on Imine and Oxime Bonds. J. Polym. Sci., Part A: Polym. Chem. 2017, 55, 3826–3831. 10.1002/pola.28856.

[ref145] Subramaniyan S.; Najjarzadeh N.; Vanga S. R.; Liguori A.; Syrén P.-O.; Hakkarainen M. Designed for Circularity: Chemically Recyclable and Enzymatically Degradable Biorenewable Schiff Base Polyester-Imines. ACS Sustainable Chem. Eng. 2023, 11, 3451–3465. 10.1021/acssuschemeng.2c06935.

[ref146] Tachibana Y.; Baba T.; Kasuya K. Environmental Biodegradation Control of Polymers by Cleavage of Disulfide Bonds. Polym. Degrad. Stab. 2017, 137, 67–74. 10.1016/j.polymdegradstab.2017.01.003.

[ref147] Lee J.; Nanthananon P.; Kim A.; Kwon Y. K. Malleable and Recyclable Thermoset Network with Reversible β-Hydroxyl Esters and Disulfide Bonds. J. Appl. Polym. Sci. 2023, 140, e53369 10.1002/app.53369.

[ref148] Di Mauro C.; Malburet S.; Genua A.; Graillot A.; Mija A. Sustainable Series of New Epoxidized Vegetable Oil-Based Thermosets with Chemical Recycling Properties. Biomacromolecules 2020, 21, 3923–3935. 10.1021/acs.biomac.0c01059. 32790997

[ref149] Ruiz de Luzuriaga A.; Martin R.; Markaide N.; Rekondo A.; Cabanero G.; Rodrıguez J.; Odriozola I. Epoxy Resin with Exchangeable Disulfide Crosslinks to Obtain Reprocessable, Repairable and Recyclable Fiber-Reinforced Thermoset Composites. Mater. Horiz. 2016, 3, 241–247. 10.1039/C6MH00029K.

[ref150] Ma Z.; Wang Y.; Zhu J.; Yu J.; Hu Z. Bio-Based Epoxy Vitrimers: Reprocessibility, Controllable Shape Memory, and Degradability. J. Polym. Sci., Part A. Polym. Chem. 2017, 55, 1790–1799. 10.1002/pola.28544.

[ref151] Yan P.; Zhao W.; Tonkin S. J.; Chalker J. M.; Schiller T. L.; Hasell T. Stretchable and Durable Inverse Vulcanized Polymers with Chemical and Thermal Recycling. Chem. Mater. 2022, 34, 1167–1178. 10.1021/acs.chemmater.1c03662.

[ref152] Tonkin S. J.; Gibson C. T.; Campbell J. A.; Lewis D. A.; Karton A.; Hasell T.; Chalker J. M. Chemically Induced Repair, Adhesion, and Recycling of Polymers Made by Inverse Vulcanization. Chem. Sci. 2020, 11, 5537–5546. 10.1039/D0SC00855A. 32874497 PMC7441575

[ref153] Li Y.; Zhang Y.; Rios O.; Keum J. K.; Kessler M. R. Photo-Responsive Liquid Crystalline Epoxy Networks with Exchangeable Disulfide Bonds. RSC Adv. 2017, 7, 37248–37254. 10.1039/C7RA06343A. 28650493

[ref154] Takahashi A.; Ohishi T.; Goseki R.; Otsuka H. Degradable Epoxy Resins Prepared from Diepoxide Monomer with Dynamic Covalent Disulfide Linkage. Polymer 2016, 82, 319–326. 10.1016/j.polymer.2015.11.057.

[ref155] Zhang Y.; Yuan L.; Liang G.; Gu A. Developing Reversible Self-Healing and Malleable Epoxy Resins with High Performance and Fast Recycling through Building Cross-Linked Network with Disulfide-Containing Hardener. Ind. Eng. Chem. Res. 2018, 57, 12397–12406. 10.1021/acs.iecr.8b02572.

[ref156] Fu S.; Rempson C. M.; Puche V.; Zhao B.; Zhang F. Construction of Disulfide Containing Redox-Responsive Polymeric Nanomedicine. Methods 2022, 199, 67–79. 10.1016/j.ymeth.2021.12.011. 34971759

[ref157] Ou M.; Wang X.-L.; Xu R.; Chang C.-W.; Bull D. A.; Kim S. W. Novel Biodegradable Poly(disulfide amine)s for Gene Delivery with High Efficiency and Low Cytotoxicity. Bioconjugate Chem. 2008, 19, 626–633. 10.1021/bc700397x. PMC2754712 18314939

[ref158] Li D.; Chen F.; Cheng C.; Li H.; Wei X. Biodegradable Materials with Disulfide-Bridged Framework Confine Photosensitizers for Enhanced Photo-Immunotherapy. Int. J. Nanomedicine. 2021, 16, 8323–8334. 10.2147/IJN.S344679. 34992368 PMC8714971

[ref159] Wan L.; Lu L.; Liang X.; Liu Z.; Huang X.; Du R.; Luo Q.; Xu Q.; Zhang Q.; Jia X. Citrate-Based Polyester Elastomer with Artificially Regulatable Degradation Rate on Demand. Biomacromolecules 2023, 24, 4123–4137. 10.1021/acs.biomac.3c00479. 37584644

[ref160] Elkassih S. A.; Kos P.; Xiong H.; Siegwart D. J. Degradable Redox-Responsive Disulfide-Based Nanogel Drug Carriers *via* Dithiol Oxidation Polymerization. Biomater. Sci. 2019, 7, 607–617. 10.1039/C8BM01120F. 30462102 PMC7031860

[ref161] Morgan B.; Sobotta M. C.; Dick T. B. Measuring E(GSH) and H_2_O_2_ with roGFP2-Based Redox Probes. Free Radical Biol. Med. 2011, 51, 1943–1951. 10.1016/j.freeradbiomed.2011.08.035. 21964034

[ref162] Østergaard H.; Tachibana C.; Winther J. R. Monitoring Disulfide Bond Formation in the Eukaryotic Cytosol. J. Cell. Biol. 2004, 166, 337–345. 10.1083/jcb.200402120. 15277542 PMC2172265

[ref163] Tsutsuba T.; Tachibana Y.; Shimizu M.; Kasuya K.-i. Marine Biodegradation of Poly(butylene succinate) Incorporating Disulfide Bonds Triggered by a Switch Function in Response to Reductive Stimuli. ACS Appl. Polym. Mater. 2023, 5, 2964–2970. 10.1021/acsapm.3c00147.

[ref164] Hu H.; Luan Q.; Li J.; Lin C.; Ouyang X.; Wei D.-Q.; Wang J.; Zhu J. High-Molecular-Weight and Light-Colored Disulfide-Bond-Embedded Polyesters: Accelerated Hydrolysis Triggered by Redox Responsiveness. Biomacromolecules 2023, 24, 5722–5736. 10.1021/acs.biomac.3c00691. 37946491

[ref165] Yamamoto T.; Goto I.; Kawaguchi O.; Minagawa K.; Ariyoshi E.; Matsuda O. Phytoremediation of Shallow Organically Enriched Marine Sediments using Benthic Microalgae. Mar. Pollut. Bull. 2008, 57, 108–115. 10.1016/j.marpolbul.2007.10.006. 18048063

[ref166] Pawar V.; Matsuda O.; Yamamoto T.; Hashimoto T.; Rajendran N. Spatial and Temporal Variations of Sediment Quality in and around Fish Cage Farms: A Case Study of Aquaculture in the Seto Inland Sea, Japan. Fish. Sci. 2001, 67, 619–627. 10.1046/j.1444-2906.2001.00298.x.

[ref167] Otsuka H.; Nagano S.; Kobashi Y.; Maeda T.; Takahara A. A Dynamic Covalent Polymer Driven by Disulfide Metathesis under Photoirradiation. Chem. Commun. 2010, 46, 1150–1152. 10.1039/B916128G. 20126743

[ref168] Kuang X.; Mu Q.; Roach D. J.; Qi H. J. Shape-Programmable and Healable Materials and Devices using Thermo- and Photo-Responsive Vitrimer. Multifunct. Mater. 2020, 3, 045001 10.1088/2399-7532/abbdc1.

[ref169] Zhu L.; Xu L.; Jie S.; Li B. Polybutadiene Vitrimers with Tunable Epoxy Ratios: Preparation and Properties. Polymers 2021, 13, 4157 10.3390/polym13234157. 34883660 PMC8659766

[ref170] Tratnik N.; Tanguy N. R.; Yan N. Recyclable, Self-Strengthening Starch-Based Epoxy Vitrimer Facilitated by Exchangeable Disulfide Bonds. Chem. Eng. J. 2023, 451, 138610 10.1016/j.cej.2022.138610.

[ref171] Lei Z. Q.; Xiang H. P.; Yuan Y. J.; Rong M. Z.; Zhang M. Q. Room-Temperature Self-Healable and Remoldable Cross-Linked Polymer Based on the Dynamic Exchange of Disulfide Bonds. Chem. Mater. 2014, 26, 2038–2046. 10.1021/cm4040616.

[ref172] Rekondo A.; Martin R.; Ruiz de Luzuriaga A.; Cabanero G.; Grande H. J.; Odriozola I. Catalyst-Free Room-Temperature Self-Healing Elastomers Based on Aromatic Disulfide Metathesis. Mater. Horiz. 2014, 1, 237–240. 10.1039/C3MH00061C.

[ref173] Wiita A. P.; Ainavarapu S. R. K.; Huang H. H.; Fernandez J. M. Force-Dependent Chemical Kinetics of Disulfide Bond Reduction Observed with Single-Molecule Techniques. Proc. Natl. Acad. Sci. U.S.A. 2006, 103, 7222–7227. 10.1073/pnas.0511035103. 16645035 PMC1464324

[ref174] Martinez-Diaz D.; Cortés A.; Jiménez-Suárez A.; Prolongo S. G. Hardener Isomerism and Content of Dynamic Disulfide Bond Effect on Chemical Recycling of Epoxy Networks. ACS Appl. Polym. Mater. 2022, 4, 5068–5076. 10.1021/acsapm.2c00598.

[ref175] Li X.; Yu R.; He Y.; Zhang Y.; Yang X.; Zhao X.; Huang W. Self-Healing Polyurethane Elastomers Based on a Disulfide Bond by Digital Light Processing 3D Printing. ACS Macro Lett. 2019, 8, 1511–1516. 10.1021/acsmacrolett.9b00766. 35651184

[ref176] Pronoitis C.; Hakkarainen M.; Odelius K. Structurally Diverse and Recyclable iIsocyanate-Free Polyurethane Networks from CO_2_-Derived Cyclic Carbonates. ACS Sustainable Chem. Eng. 2022, 10, 2522–2531. 10.1021/acssuschemeng.1c08530.

[ref177] Yang Y.; Xu Y.; Ji Y.; Wei Y. Functional Epoxy Vitrimers and Composites. Prog. Mater. Sci. 2021, 120, 100710 10.1016/j.pmatsci.2020.100710.

[ref178] Ussama W.; Shibata M. Self-Healing Polyester Networks Prepared from Poly(butylene succinate-co-butylene itaconate) and Thiol-Terminated Polyether Containing Disulfide Linkages. Polymer 2022, 244, 124668 10.1016/j.polymer.2022.124668.

[ref179] Ussama W.; Shibata M. Self-Healing Disulfide-Containing Polyester-Urethane Networks Composed of 6-Armed Star-Shaped Oligolactide and Oligocaprolactone Segments. J. Polym. Res. 2021, 28, 5 10.1007/s10965-020-02360-6.

[ref180] Borska K.; Bednarek M.; Pawlak A. Reprocessable Polylactide-Based Network Containing Urethane and Disulfide Linkages. Eur. Polym. J. 2021, 156, 110636 10.1016/j.eurpolymj.2021.110636.

[ref181] Chen M.; Zhou L.; Wu Y.; Zhao X.; Zhang Y. Rapid Stress Relaxation and Moderate Temperature of Malleability Enabled by the Synergy of Disulfide Metathesis and Carboxylate Transesterification in Epoxy Vitrimers. ACS Macro Lett. 2019, 8, 255–260. 10.1021/acsmacrolett.9b00015. 35650825

[ref182] Yuan W.; Liu G.; Huang C.; Li Y.; Zeng J. Highly Stretchable, Recyclable, and Fast Room Temperature Self-Healable Biobased Elastomers using Polycondensation. Macromolecules 2020, 53, 9847–9858. 10.1021/acs.macromol.0c01665.

[ref183] Wang D.; Tang Z.; Wang Z.; Zhang L.; Guo B. A Bio-Based, Robust and Recyclable Thermoset Polyester Elastomer by Using in Inverse Vulcanized Polysulfide as a Crosslinker. Polym. Chem. 2022, 13, 485–491. 10.1039/D1PY01287H.

[ref184] Li J.; Wang Y.; Wang X.; Wu D. Crystalline Characteristics, Mechanical Properties, Thermal Degradation Kinetics and Hydration Behavior of Biodegradable Fibers Melt-Spun from Polyoxymethylene/Poly(l-lactic acid) Blends. Polymers 2019, 11, 1753 10.3390/polym11111753. 31731470 PMC6918227

[ref185] Heffernan M. J.; Murthy N. Polyketal Nanoparticles: A New pH-Sensitive Biodegradable Drug Delivery Vehicle. Bioconjugate Chem. 2005, 16, 1340–1342. 10.1021/bc050176w. 16287226

[ref186] Nomura M.; Shuto S.; Matsuda A. Synthesis of the Cyclic and Acyclic Acetal Derivatives of 1-(3-C-Ethynyl-D-ribo-pentofuranosyl)cytosine, a Potent Antitumor Nucleoside. Design of Prodrugs to be Selectively Activated in Tumor Tissues via the Bio-Reduction-Hydrolysis Mechanism. Bioorg. Med. Chem. 2003, 11, 2453–2461. 10.1016/S0968-0896(03)00104-4. 12735992

[ref187] Abel B. A.; Snyder R. L.; Coates G. W. Chemically Recyclable Thermoplastics from Reversible-Deactivation of Cyclic Acetals. Science 2021, 373, 783–789. 10.1126/science.abh0626. 34385394

[ref188] Hufendiek A.; Lingier S.; Du Prez F. E. Thermoplastic Polyacetals: Chemistry from the Past for a Sustainable Future?. Polym. Chem. 2019, 10, 9–33. 10.1039/C8PY01219A.

[ref189] Li Q.; Ma S.; Wang S.; Yuan W.; Xu X.; Wang B.; Huang K.; Zhu J. Facile Catalyst-Free Synthesis, Exchanging, and Hydrolysis of an Acetal Motif for Dynamic Covalent Networks. J. Mater. Chem. A 2019, 7, 18039–18049. 10.1039/C9TA04073K.

[ref190] Ma S.; Wei J.; Jia Z.; Yu T.; Yuan W.; Li Q.; Wang S.; You S.; Liu R.; Zhu J. Readily Recyclable, High-Performance Thermosetting Materials Based on a Lignin-Derived Spiro Diacetal Trigger. J. Mater. Chem. A 2019, 7, 1233–1243. 10.1039/C8TA07140C.

[ref191] Hayashi S.; Tachibana Y.; Tabata N.; Kasuya K. -i. Chemically Recyclable Bio-Based Polyester Composed of Bifuran and Glycerol Acetal. Eur. Polym. J. 2021, 145, 110242 10.1016/j.eurpolymj.2020.110242.

[ref192] Zhang X.; Guo W.; Zhang C.; Zhang X. A Recyclable Polyester Library from Reversible Alternating Copolymerization of Aldehyde and Cyclic Anhydride. Nat. Commun. 2023, 14, 5423 10.1038/s41467-023-41136-6. 37669954 PMC10480228

[ref193] Mankar S. V.; Wahlberg J.; Warlin N.; Valsange N. G.; Rehnberg N.; Lundmark S.; Jannasch P.; Zhang B. Short-Loop Chemical Recycling via Telechelic Polymers for Biobased Polyesters with Spiroacetal Units. ACS Sustainable. Chem. Eng. 2023, 11, 5135–6146. 10.1021/acssuschemeng.2c07176.

[ref194] Hu K.; Wang B.; Xu X.; Su Y.; Zhang W.; Zhou S.; Zhang C.; Zhu J.; Ma S. Dual-Dynamic Chemistries-Based Fast-Reprocessing and High-Performance Covalent Adaptable Networks. Macromol. Rapid Commun. 2023, 44, 2200726 10.1002/marc.202200726. 36250433

[ref195] Mankar S. V.; Garcia Gonzalez M. N.; Warlin N.; Valsange N. G.; Rehnberg N.; Lundmark S.; Jannasch P.; Zhang B. Synthesis, Life Cycle Assessment, and Polymerization of a Vanillin-Based Spirocyclic Diol toward Polyesters with Increased Glass-Transition Temperature. ACS Sustainable Chem. Eng. 2019, 7, 19090–19103. 10.1021/acssuschemeng.9b04930.

[ref196] Trifoi A. R.; Agachi P. S.; Pap T. Glycerol Acetals and Ketals as Possible Diesel Additives. A Review of Their Synthesis Protocols. Renewable Sustainable Energy Rev. 2016, 62, 804–814. 10.1016/j.rser.2016.05.013.

[ref197] Li K.; Sun X.; Yin H.; Yang K.; Dai P.; Han L.; Zhang L.; Xu R. Vanillin- and Isovanillin-Based Phthalonitrile Resins Containing Spirocycle Acetal Structures. ACS Appl. Polym. Mater. 2023, 5, 7878–7886. 10.1021/acsapm.3c01138.

[ref198] Kirchhecker S.; Dell’Acqua A.; Angenvoort A.; Spannenberg A.; Ito K.; Tin S.; Taden A.; de Vries J. G. HMF–Glycerol Acetals as Additives for the Debonding of Polyurethane Adhesives. Green Chem. 2021, 23, 957–965. 10.1039/D0GC04093B.

[ref199] Moreno A.; Morsali M.; Sipponen M. H. Catalyst-Free Synthesis of Lignin Vitrimers with Tunable Mechanical Properties: Circular Polymers and Recoverable Adhesives. ACS Appl. Mater. Interfaces 2021, 13, 57952–57961. 10.1021/acsami.1c17412. 34813290 PMC8662642

[ref200] Hu H.; Tian Y.; Kong Z.; Ying W. B.; Chen C.; Li F.; Zhang R.; Zhu J. A High Performance Copolyester with “Locked” Biodegradability: Solid Stability and Controlled Degradation Enabled by Acid-Labile Acetal. ACS Sustainable Chem. Eng. 2021, 9, 2280–2290. 10.1021/acssuschemeng.0c08274.

[ref201] Tian Y.; Li J.; Hu H.; Chen C.; Li F.; Ying W. B.; Zheng L.; Zhao Y.-L.; Wang J.; Zhang R.; et al. Acid-Triggered, Degradable and High Strength-Toughness Copolyesters: Comprehensive Experimental and Theoretical Study. J. Hazard. Mater. 2022, 430, 128392 10.1016/j.jhazmat.2022.128392. 35152100

[ref202] Neitzel A. E.; Barreda L.; Trotta J. T.; Fahnhorst G. W.; Haversang T. J.; Hoye T. R.; Fors B. P.; Hillmyer M. A. Hydrolytically-Degradable Homo- and Copolymers of a Strained Exocyclic Hemiacetal Ester. Polym. Chem. 2019, 10, 4573–4583. 10.1039/C9PY00797K.

[ref203] Neitzel A. E.; Petersen M. A.; Kokkoli E.; Hillmyer M. A. Divergent Mechanistic Avenues to an Aliphatic Polyesteracetal or Polyester from a Single Cyclic Esteracetal. ACS Macro Lett. 2014, 3, 1156–1160. 10.1021/mz5005794. 35610815

[ref204] Lavilla C.; Alla A.; Martinez de Ilarduya A.; Benito E.; Garcia-Martin M. G.; Galbis J. A.; Munoz-Guerra S. Carbohydrate-Based Polyesters Made from Bicyclic Acetalized Galactaric Acid. Biomacromolecules 2011, 12, 2642–2652. 10.1021/bm200445w. 21563782

[ref205] Lavilla C.; Munoz-Guerra S. Biodegradation and Hydrolytic Degradation of Poly(butylene terephthalate) Copolyesters Containing Cyclic Sugar Units. Polym. Degrad. Stab. 2012, 97, 1762–1771. 10.1016/j.polymdegradstab.2012.06.008.

[ref206] Martin R. T.; Camargo L. P.; Miller S. A. Marine-Degradable Polylactic Acid. Green Chem. 2014, 16, 1768–1773. 10.1039/C3GC42604A.

[ref207] Hakkarainen M.; Albertsson A.-C.; Karlsson S. Weight Losses and Molecular Weight Changes Correlated with the Evolution of Hydroxyacids in Simulated *In Vivo* Degradation of Homo-and Copolymers of PLA and PGA. Polym. Degrad. Stab. 1996, 52, 283–291. 10.1016/0141-3910(96)00009-2.

[ref208] Kim J. S.; Jo S. D.; Seah G. L.; Kim I.; Nam Y. S. ROS-Induced Biodegradable Polythioketal Nanoparticles for Intracellular Delivery of Anti-Cancer Therapeutic. J. Ind. Eng. Chem. 2015, 21, 1137–1142. 10.1016/j.jiec.2014.05.026.

[ref209] Martin J. R.; Gupta M. K.; Page J. M.; Yu F.; Davidson J. M.; Guelcher S. A.; Duvall C. L. A Porous Tissue Engineering Scaffold Selectively Degraded by Cell-Generated Reactive Oxygen Species. Biomaterials 2014, 35, 3766 10.1016/j.biomaterials.2014.01.026. 24491510 PMC3975079

[ref210] Wilson D. S.; Dalmasso G.; Wang L.; Sitaraman S. V.; Merlin D.; Murthy N. Orally Delivered Thioketal Nanoparticles Loaded with TNF-α–siRNA Target Inflammation and Inhibit Gene Expression in the Intestines. Nature 2010, 9, 923–928. 10.1038/nmat2859. PMC3142359 20935658

[ref211] Xu L.; Zhao M.; Zhang H.; Gao W.; Guo Z.; Zhang X.; Zhang J.; Cao J.; Pu Y.; He B. Cinnamaldehyde-Based Poly(ester-thioacetal) to Generate Reactive Oxygen Species for Fabricating Reactive Oxygen Species-Responsive Nanoparticles. Biomacromolecules 2018, 19, 4658–4667. 10.1021/acs.biomac.8b01423. 30418756

[ref212] Abdelrahim M.; Zhang J.; Gao Q.; Zordok W. A.; Liu J.; Geng J. Oxidative Degradation of Thermosets Based on Thioketal Cleavable Linkages in Aqueous Environment. ACS Appl. Polym. Mater. 2022, 4, 7812–7822. 10.1021/acsapm.2c01357.

[ref213] De Schryver F. C.; Anand L.; Smets G.; Switten J. Photopolymerization. IV. Photopolymerization of Bisanthracenes. J. Polym. Sci., Part B: Polym. Lett. 1971, 9, 777–780. 10.1002/pol.1971.110091014.

[ref214] Kaur G.; Johnston P.; Saito K. Photo-Reversible Dimerisation Reactions and Their Applications in Polymeric Systems. Polym. Chem. 2014, 5, 2171–2186. 10.1039/C3PY01234D.

[ref215] Takada K. Synthesis of Biobased Functional Materials Using Photoactive Cinnamate Derivatives. Polym. J. 2023, 55, 1023–1033. 10.1038/s41428-023-00804-6.

[ref216] Cazin I.; Rossegger E.; Guedes de la Cruz G.; Griesser T.; Schlögl S. Recent Advances in Functional Polymers Containing Coumarin chromophores. Polymers 2021, 13, 56 10.3390/polym13010056. PMC7794725 33375724

[ref217] Wolff T.; Görner H. Photocleavage of Dimers of Coumarin and 6-Alkylcoumarins. J. Photochem, Photobiol., A 2010, 209, 219–223. 10.1016/j.jphotochem.2009.11.018.

[ref218] Van Damme J.; Du Prez F. Anthracene-Containing Polymers Toward High-End Applications. Prog. Polym. Sci. 2018, 82, 92–119. 10.1016/j.progpolymsci.2018.02.002.

[ref219] Bouas-Laurent H.; Castellan A.; Desvergne J.-P.; Lapouyade R. Photodimerization of Anthracenes in Fluid Solutions: (Part 2) Mechanistic Aspects of the Photocycloaddition and of the Photochemical and Thermal Cleavage. Chem. Soc. Rev. 2001, 30, 248–263. 10.1039/b006013p.

[ref220] Tong R.; Hemmati H. D.; Langer R.; Kohane D. S Photoswitchable Nanoparticles for Triggered Tissue Penetration and Drug Delivery. J. Am. Chem. Soc. 2012, 134, 8848–8855. 10.1021/ja211888a. 22385538 PMC3363369

[ref221] Cacciarini M.; Skov A. B.; Jevric M.; Hansen A. S.; Elm J.; Kjaergaard H. G.; Mikkelsen K. V.; Bro̷ndsted Nielsen M. Towards Solar Energy Storage in the Photochromic Dihydroazulene-Vinylheptafulvene System. Chem. - Eur. J. 2015, 21, 7454 10.1002/chem.201500100. 25847100

[ref222] Lendlein A.; Jiang H.; Jünger O.; Langer R. Light-Induced Shape-Memory Polymers. Nature 2005, 434, 879–882. 10.1038/nature03496. 15829960

[ref223] Hughes T.; Simon G. P.; Saito K. Chemistries and Capabilities of Photo-Formable and Photoreversible Crosslinked Polymer Networks. Mater. Horiz. 2019, 6, 1762–1773. 10.1039/C9MH00217K.

[ref224] Seoane Rivero R.; Bilbao Solaguren P.; Gondra Zubieta K.; Gonzalez-Jimenez A.; Valentin J. L.; Marcos-Fernandez A. Synthesis and Characterization of a Photo-Crosslinkable Polyurethane Based on a Coumarin-Containing Polycaprolactone Diol. Eur. Polym. J. 2016, 76, 245–255. 10.1016/j.eurpolymj.2016.01.047.

[ref225] Navarro R.; Seoane-Rivero R.; Cuevas J. M.; Marcos-Fernandez Á. A Novel Strategy to Polyurethanes with Improved Mechanical Properties by Photoactivation of Amidocoumarin Moieties. RSC Adv. 2020, 10, 29935–29944. 10.1039/D0RA06372J. 35518219 PMC9056287

[ref226] Seoane Rivero R.; Bilbao Solaguren P.; Gondra Zubieta K.; Peponi L.; Marcos-Fernández A. Synthesis, Kinetics of Photo-Dimerization/Photo-Cleavage and Physical Properties of Coumarin-Containing Branched Polyurethanes Based on Polycaprolactones. Express Polym. Lett. 2016, 10, 84–95. 10.3144/expresspolymlett.2016.10.

[ref227] Kaiser S.; Radl S. V.; Manhart J.; Ayalur-Karunakaran S.; Griesser T.; Moser A.; Ganser C.; Teichert C.; Kern W.; Schlögl S. Switching “On” and “Off” the Adhesion in Stimuli-Responsive Elastomers. Soft Matt. 2018, 14, 2547–2559. 10.1039/C8SM00284C. 29541729

[ref228] Feng Y.; Han X.; Ji Y.; Li S.; Dong K.; Liang S.; Ma Y.; Yang Y.; Liu F. The Reversible Modification of Acrylate Adhesive System by Introducing Anthracene Groups. J. Adhes. 2024, 100, 200–218. 10.1080/00218464.2023.2211930.

[ref229] Inada M.; Horii T.; Fujie T.; Nakanishi T.; Asahi T.; Saito K. Debonding-on-Demand Adhesives Based on Photo-Reversible Cycloaddition Reactions. Mater. Adv. 2023, 4, 1289–1296. 10.1039/D2MA01048H.

[ref230] Boga K.; Patti A. F.; Warner J. C.; Simon G. P.; Saito K. Eco-Friendly Photoreversible Adhesives Derived from Coumarin-Functionalized Epoxy Soybean Oil. ACS Appl. Polym. Mater. 2023, 5, 4644–4653. 10.1021/acsapm.3c00091.

[ref231] Shen L.; Cheng J.; Zhang J. Reworkable Adhesives: Healable and Fast Response at Ambient Environment Based on Anthracene-Based Thiol-Ene Networks. Eur. Polym. J. 2020, 137, 109927 10.1016/j.eurpolymj.2020.109927.

[ref232] Liu Z.; Cheng J.; Zhang J. An Efficiently Reworkable Thermosetting Adhesive Based on Photoreversible [4 + 4] Cycloaddition Reaction of Epoxy-Based Prepolymer with Four Anthracene End Groups. Macromol. Chem. Phys. 2021, 222, 2000298 10.1002/macp.202000298.

[ref233] Liu Z.; Wang G.; Cheng J.; Zhang J. Crystalline Segments in a Photo-Detachable Adhesive. Eur. Polym. J. 2021, 152, 110472 10.1016/j.eurpolymj.2021.110472.

[ref234] Maddipatla M. V. S. N.; Wehrung D.; Tang C.; Fan W.; Oyewumi M. O.; Miyoshi T.; Joy A. Photoresponsive Coumarin Polyesters that Exhibit Cross-Linking and Chain Scission Properties. Macromolecules 2013, 46, 5133–5140. 10.1021/ma400584y.

[ref235] Chamsaz E. A.; Sun S.; Maddipatla M. V. S. N.; Joy A. Photoresponsive Polyesters by Incorporation of Alkoxyphenacyl or Coumarin Chromophores Along the Backbone. Photochem. Photobiol. Sci. 2014, 13, 412–421. 10.1039/c3pp50311a. 24407420

[ref236] Nagata M.; Yamamoto Y. Photocurable Shape-Memory Copolymers of ε- Caprolactone and L-Lactide. Macromol. Chem. Phys. 2010, 211, 1826–1835. 10.1002/macp.201000106.

[ref237] Defize T.; Thomassin J.-M.; Ottevaere H.; Malherbe C.; Eppe G.; Jellali R.; Alexandre M.; Jérôme C.; Riva R. Photo-Cross-Linkable Coumarin-Based Poly(ε-Caprolactone) for Light-Controlled Design and Reconfiguration of Shape-Memory Polymer Networks. Macromolecules 2019, 52, 444–456. 10.1021/acs.macromol.8b02188.

[ref238] Xie H.; He M-j; Deng X.-Y.; Du L.; Fan C.-J.; Yang K.-K.; Wang Y.-Z. Design of Poly(L-Lactide)-Poly(Ethylene Glycol) Copolymer with Light-Induced Shape-Memory Effect Triggered by Pendant Anthracene Groups. ACS Appl. Mater. Interfaces 2016, 8, 9431–9439. 10.1021/acsami.6b00704. 27031590

[ref239] Rochette J. M.; Ashby V. S. Photoresponsive Polyesters for Tailorable Shape Memory Biomaterials. Macromolecules 2013, 46, 2134–2140. 10.1021/ma302354a.

[ref240] Chen Y.; Hong R.-T. Synthesis of Polyesters Containing Coumarin Dimer Components by Photopolymerization of 7,7’-Coumarinyl Polymethylene Dicarboxylates. J. Polym. Res. 1994, 1, 285–293. 10.1007/BF01374553.

[ref241] Chen Y.; Jean C.-S. Polyethers Containing Coumarin Dimer Components in the Main Chain. II. Reversible Photocleavage and Photopolymerization. J. Appl. Polym. Sci. 1997, 64, 1759–1768. 10.1002/(SICI)1097-4628(19970531)64:9<1759::AID-APP12>3.0.CO;2-T.

[ref242] Xue Y.; Jiang S.; Zhong H.; Chen Z.; Wang F. Photo-Induced Polymer Cyclization via Supramolecular Confinement of Cyanostilbenes. Angew. Chem., Int. Ed. 2022, 61, e202110766 10.1002/anie.202110766. 34714571

[ref243] Yamamoto T.; Yagyu S.; Tezuka Y. Light- and Heat-Triggered Reversible Linear–Cyclic Topological Conversion of Telechelic Polymers with Anthryl End Groups. J. Am. Chem. Soc. 2016, 138, 3904–3911. 10.1021/jacs.6b00800. 26916947

[ref244] Frisch H.; Mundsinger K.; Poad B. L. J.; Blanksby S. J.; Barner-Kowollik C. Wavelength-Gated Photoreversible Polymerization and Topology Control. Chem. Sci. 2020, 11, 2834–2842. 10.1039/C9SC05381F. 32206267 PMC7069517

[ref245] Chen L.; Wang X.; Hou R.; Lu H.; He Z.; Zhou X.; Zhang W.; Wang X. Efficient Preparation of Cyclic Polymers via Pre-Stacking of Photo-Cycloaddition Capable End Groups and a Continuous-Flow Technique. Polym. Chem. 2023, 14, 4659–4670. 10.1039/D3PY00935A.

[ref246] Wang H.; Zhang L.; Liu B.; Han B.; Duan Z.; Qi C.; Park D.-W.; Kim I. Synthesis of High Molecular Weight Cyclic Poly(ε-caprolactone)s of Variable Ring Size Based on a Light-Induced Ring-Closure Approach. Macromol. Rapid Commun. 2015, 36, 1646–1650. 10.1002/marc.201500171. 26174587

[ref247] Du Z.; Shan Y.; Luo J.; Sun N.; Ren B. Linear Alternating Associative Polymer with Ultrahigh Molecular Weight: Facile Preparation by Self-Assembly Assisted Dimerization of Anthracene and Rheology in Aqueous Solution. ACS Macro Lett. 2019, 8, 279–284. 10.1021/acsmacrolett.9b00028. 35650829

[ref248] Du Z.; Yan X.; Dong R.; Ke K.; Ren B.; Tong Z. Unusual Transient Network and Rheology of a Photoresponsive Telechelic Associative Model Polymer in Aqueous Solution Induced by Dimerization of Coumarin End Groups. Macromolecules 2018, 51, 1518–1528. 10.1021/acs.macromol.7b01514.

[ref249] Baysak E.; Durmaz H.; Tunca U.; Hizal G. Synthesis of Activated Ester Functional Polyesters through Light-Induced [4 + 4] Cycloaddition Polymerization. Macromol. Chem. Phys. 2017, 218, 1600572 10.1002/macp.201600572.

[ref250] Van Damme J.; Vlaminck L.; Van Assche G.; Van Mele B.; van den Berg O.; Du Prez F. Synthesis and Evaluation of 9-Substituted Anthracenes with Potential in Reversible Polymer Systems. Tetrahedron 2016, 72, 4303–4311. 10.1016/j.tet.2016.05.077.

[ref251] Van Damme J.; van den Berg O.; Brancart J.; Van Assche G.; Du Prez F. A Novel Donor-p-Acceptor Anthracene Monomer: Towards Faster and Milder Reversible Dimerization. Tetrahedron 2019, 75, 912–920. 10.1016/j.tet.2019.01.007.

[ref252] Inacker S.; Kahler P.; Hampp N. Enhancing the photochemical reversibility of coumarin-containing polymers by molecular orientation control. Polym. Chem. 2022, 13, 6238–6245. 10.1039/D2PY01230H.

[ref253] Momeni S.; Craplewe K.; Safder M.; Luz S.; Sauvageau D.; Elias A. Accelerating the Biodegradation of Poly(lactic acid) through the Inclusion of Plant Fibers: A Review of Recent Advances. ACS Sustainable. Chem. Eng. 2023, 11, 15146–15170. 10.1021/acssuschemeng.3c04240. 37886036 PMC10599323

[ref254] Miyauchi M.; Li Y.; Shimizu H. Enhanced Degradation in Nanocomposites of TiO_2_ and Biodegradable Polymer. Environ. Sci. Technol. 2008, 42, 4551–4554. 10.1021/es800097n. 18605585

[ref255] Yew S.-P.; Tang H.-Y.; Sudesh K. Photocatalytic Activity and Biodegradation of Polyhydroxybutyrate Films Containing Titanium Dioxide. Polym. Degrad. Stab. 2006, 91, 1800–1807. 10.1016/j.polymdegradstab.2005.11.011.

[ref256] Tateiwa J.; Kimura S.; Kasuya K. -i.; Iwata T. Multilayer Biodegradable Films with a Degradation Initiation Function Triggered by Weakly Alkaline Water. Polym. Degrad. Stab. 2022, 200, 109942 10.1016/j.polymdegradstab.2022.109942.

[ref257] Tomifuji R.; Maeda K.; Takahashi T.; Kurahashi T.; Matsubara S. FeCl_3_ as an Ion-Pairing Lewis Acid Catalyst. Formation of Highly Lewis Acidic FeCl_2_^+^ and Thermodynamically Stable FeCl_4_^–^ to Catalyze the Aza-Diels-Alder Reaction with High Turnover Frequency. Org. Lett. 2018, 20, 7474–7477. 10.1021/acs.orglett.8b03249. 30427692

[ref258] Li L.; Gong S.; Yang L.; Zhang F.; Xie L. J.; Luo Z.; Xia X. X.; Wang J. Study on the Degradation Behavior and Mechanism of Poly(lactic acid) Modification by Ferric Chloride. Polymer 2020, 188, 121991 10.1016/j.polymer.2019.121991.

[ref259] Li X.; Gong S.; Yang L.; Xia X.; Linghu C.; Wang J.; Luo Z. Transesterification Catalyzed via Ferric Chloride for Fabricating Poly(lactic acid)/Poly(butylene adipate-co-terephthalate) Blends with Ultra-Fast Degradation and High Toughness. Polymer 2021, 228, 123927 10.1016/j.polymer.2021.123927.

[ref260] Ganesh M.; Dave R. N.; L’Amoreaux W.; Gross R. A. Embedded Enzymatic Biomaterial Degradation. Macromolecules 2009, 42, 6836–6839. 10.1021/ma901481h.

[ref261] DelRe C.; Jiang Y.; Kang P.; Kwon J.; Hall A.; Jayapurna I.; Ruan Z.; Ma L.; Zolkin K.; Li T.; et al. Near-Complete Depolymerization of Polyesters with Nano-Dispersed Enzymes. Nature 2021, 592, 558–563. 10.1038/s41586-021-03408-3. 33883730

[ref262] Chu C. C. Hydrolytic Degradation of Polyglycolic Acid: Tensile Strength and Crystallinity Study. J. Appl. Polym. Sci. 1981, 26, 1727–1734. 10.1002/app.1981.070260527.

[ref263] Penas M. I.; Criado-Gonzalez M.; Martinez de Ilarduya A.; Flores A.; Raquez J.-M.; Mincheva R.; Müller A. J.; Hernández R. Tunable Enzymatic Biodegradation of Poly(butylene succinate): Biobased Coatings and Self-Degradable Films. Polym. Degrad. Stab. 2023, 211, 110341 10.1016/j.polymdegradstab.2023.110341.

[ref264] Huang Q. Y.; Hiyama M.; Kabe T.; Kimura S.; Iwata T. Enzymatic Self-Biodegradation of Poly(L-lactic acid) Films by Embedded Heat-Treated and Immobilized Proteinase K. Biomacromolecules 2020, 21, 3301–3307. 10.1021/acs.biomac.0c00759. 32678613

[ref265] Liu M.; Zhang T.; Long L.; Zhang R.; Ding S. Efficient Enzymatic Degradation of Poly(ε-caprolactone) by an Engineered Bifunctional Lipase-Cutinase. Polym. Degrad. Stab. 2019, 160, 120–125. 10.1016/j.polymdegradstab.2018.12.020.

[ref266] Greene A. F.; Vaidya A.; Collet C.; Wade K. R.; Patel M.; Gaugler M.; West M.; Petcu M.; Parker K. 3D-Printed Enzyme-Embedded Plastics. Biomacromolecules 2021, 22, 1999–2009. 10.1021/acs.biomac.1c00105. 33870685

[ref267] Huang Q. Y.; Kimura S.; Iwata T. Development of Self-Degradable Aliphatic Polyesters by Embedding Lipases via Melt Extrusion. Polym. Degrad. Stab. 2021, 190, 109647 10.1016/j.polymdegradstab.2021.109647.

[ref268] Huang Q.; Kimura S.; Iwata T. Thermal Embedding of *Humicola insolens* Cutinase: A Strategy for Improving Polyester Biodegradation in Seawater. Biomacromolecules 2023, 24, 5836–5846. 10.1021/acs.biomac.3c00835. 37940601

[ref269] Kalita N. K.; Hakkarainen M. Triggering Degradation of Cellulose Acetate by Embedded Enzymes: Accelerated Enzymatic Degradation and Biodegradation Under Simulated composting conditions. Biomacromolecules 2023, 24, 3290–3303. 10.1021/acs.biomac.3c00337. 37347240 PMC10336969

[ref270] Guo B.; Lopez-Lorenzo X.; Fang Y.; Bäckström E.; Capezza A. J.; Reddy Vanda S.; Furó I.; Hakkarainen M.; Syrén P.-O. Fast Depolymerization of PET Bottle Mediated by Microwave Pre-Treatment and an Engineered PETase. ChemSusChem 2023, 16, e202300742 10.1002/cssc.202300742. 37384425

[ref271] Lu H.; Diaz D. J.; Czarnecki N. J.; Zhu C.; Kim W.; Shroff R.; Acosta D. J.; Alexander B. R.; Cole H. O.; Zhang Y.; et al. Machine Learning-Aided Engineering of Hydrolases for PET Depolymerization. Nature 2022, 604, 662–667. 10.1038/s41586-022-04599-z. 35478237

[ref272] DelRe C.; Chang B.; Jayapurna I.; Hall A.; Wang A.; Zolkin K.; Xu T. Synergistic Enzyme Mixtures to Realize Near-Complete Depolymerization in Biodegradable Polymer/Additive Blends. Adv. Mater. 2021, 33, 2105707 10.1002/adma.202105707. 34623716

[ref273] Häussler M.; Eck M.; Rothauer D.; Mecking S. Closed-Loop Recycling of Polyethylene-Like Materials. Nature 2021, 590, 423–427. 10.1038/s41586-020-03149-9. 33597754

[ref274] Stempfle F.; Quinzler D.; Heckler I.; Mecking S. Long-Chain Linear C19 and C23 Monomers and Polycondensates from Unsaturated Fatty Acid Esters. Macromolecules 2011, 44, 4159–4166. 10.1021/ma200627e.

[ref275] Myers D.; Witt T.; Cyriac A.; Bown M.; Mecking S.; Williams C. K. Ring Opening Polymerization of Macrolactones: High Conversions and Activities Using an Yttrium Catalyst. Polym. Chem. 2017, 8, 5780–5785. 10.1039/C7PY00985B.

[ref276] Van der Meulen I.; Gubbels E.; Huijser S.; Sablong R.; Koning C. E.; Heise A.; Duchateau R. Catalytic Ring-Opening Polymerization of Renewable Macrolactones to High Molecular Weight Polyethylene-Like Polymers. Macromolecules 2011, 44, 4301–4305. 10.1021/ma200685u.

[ref277] Roy P. K.; Hakkarainen M.; Varma I. K.; Albertsson A.-C. Albertsson, Degradable Polyethylene: Fantasy or Reality. A.-C. Environ. Sci. Technol. 2011, 45, 4217–4227. 10.1021/es104042f. 21495645

[ref278] Bailey W. J.; Gapud B. Synthesis of Biodegradable Polyethylene. ACS Symp. Ser. 1985, 280, 423 10.1021/bk-1985-0280.ch029.

[ref279] Focarete M. L.; Scandola M.; Kumar A.; Gross R. A. Physical Characterization of Poly(ω-Pentadecalactone) Synthesized by Lipase-Catalyzed Ring-Opening Polymerization. J. Polym. Sci., Part B: Polym. Phys. 2001, 39, 1721 10.1002/polb.1145.

[ref280] van der Meulen I.; de Geus M.; Antheunis H.; Deumens R.; Joosten E. A. J.; Koning C. E.; Heise A. Polymers from Functional Macrolactones as Potential Biomaterials: Enzymatic Ring Opening Polymerization, Biodegradation, and Biocompatibility. Biomacromolecules 2008, 9, 3404–3410. 10.1021/bm800898c. 18975906

[ref281] Zhou L.; Qin P.; Wu L.; Li B.-G.; Dubois P. Potentially Biodegradable “Short-Long” Type Diol-Diacid Polyesters with Superior Crystallizability, Tensile Modulus, and Water Vapor Barrier. ACS Sustainable. Chem. Eng. 2021, 9, 17362–17370. 10.1021/acssuschemeng.1c06752.

[ref282] Tachibana Y.; Tsutsuba T.; Kageyama K.; Kasua K. -i. Biodegradability of Poly(butylene n-alkylenedionate)s Composed of Long-Methylene Chains as alternative Polymers to Polyethylene. Polym. Degrad. Stab. 2021, 190, 109650 10.1016/j.polymdegradstab.2021.109650.

[ref283] Eck M.; Schwab S. T.; Nelson T. F.; Wurst K.; Iberl S.; Schleheck D.; Link C.; Battagliarin G.; Mecking S. Biodegradable High-Density Polyethylene-Like Material. Angew. Chem., Int. Ed. 2023, 62, e202213438 10.1002/anie.202213438. PMC10107712 36480133

[ref284] Eck M.; Bernabeu L.; Mecking S. Polyethylene-Like Blends Ameable to Abiotic Hydrolytic Degradation. ACS Sustainable. Chem. Eng. 2023, 11, 4523–4530. 10.1021/acssuschemeng.2c07537. 37008182 PMC10052336

[ref285] Zhang F.; Zeng M.; Yappert R. D.; Sun J.; Lee Y.-H.; LaPointe A. M.; Peters B.; Abu-Omar M. M.; Scott S. L. Polyethylene Upcycling to Long-Chain Alkylaromatics by Tandem Hydrogenolysis/Aromatization. Science 2020, 370, 437–441. 10.1126/science.abc5441. 33093105

[ref286] Jehanno C.; Alty J. W.; Roosen M.; De Meester S.; Dove A. P.; Chen E. Y.-X.; Leibfarth F. A.; Sardon H. Critical Advances and Future Opportunities in Upcycling Commodity Plastics. Nature 2022, 603, 803–814. 10.1038/s41586-021-04350-0. 35354997

[ref287] Arroyave A.; Cui S.; Lopez J. C.; Kocen A. L.; LaPointe A. M.; Delferro M.; Coates G. W. Catalytic Chemical Recycling of Post-Consumer Polyethylene. J. Am. Chem. Soc. 2022, 144, 23280–23285. 10.1021/jacs.2c11949. 36524740

[ref288] Bäckström E.; Odelius K.; Hakkarainen M. Trash to Treasure: Microwave-Assisted Conversion of Polyethylene to Functional Chemicals. Ind. Eng. Chem. Res. 2017, 56, 14814–14821. 10.1021/acs.iecr.7b04091.

[ref289] Zeng M.; Lee Y.-H.; Strong G.; LaPointe A. M.; Kocen A. L.; Qu Z.; Coates G. W.; Scott S. L.; Abu-Omar M. M. Chemical Upcycling to Value-Added α,ω-Divinyl-Functionalized Oligomers. ACS Sustainable Chem. Eng. 2021, 9, 13926 10.1021/acssuschemeng.1c05272.

[ref290] Wu Q.; Qin K.-X.; Gan M.-X.; Xu J.; Li Z.-L.; Li Z.-C. Recyclable Biomass-Derived Polyethylene-Like Materials as Functional Coatings for Commercial Fabrics: Toward Upcycling of Waste Textiles. ACS Sustainable. Chem. Eng. 2022, 10, 17187–17197. 10.1021/acssuschemeng.2c05080.

[ref291] Kocen A. L.; Cui S.; Lin T.-W.; LaPointe A. M.; Coates G. W. Chemically Recyclable Ester-Linked Polypropylene. J. Am. Chem. Soc. 2022, 144, 12613–12618. 10.1021/jacs.2c04499. 35793702

[ref292] Martinez-Cutillas A.; Léon S.; Oh S.; Martinez de Ilarduya A. Enzymatic Recycling of Polymacrolactones. Polym. Chem. 2022, 13, 1586 10.1039/D1PY01721G.

